# Supplementary material for: Whole-Genome DNA Methylation Analysis in Hydrogen Peroxide Overproducing Transgenic Tobacco Resistant to Biotic and Abiotic Stresses
Source: Plants (Basel). 2021 Jan 19;10(1):178. doi: 10.3390/plants10010178 (PMC7835756; doi:10.3390/plants10010178)
Supplement: Supplementary file 1 [file plants-10-00178-s001.pdf]

Table S1. Statistics Results of data production. In the table it is showed the data statistic result proportioned by BGI. This data is produced after treated the raw reads of each sequencing library by filtering, which includes removing adaptor sequences, contamination, and low-quality reads from raw reads. The score Q20 (%) represents the number of nucleotide with quality higher than 20/nucleotide. The GC (%) corresponds to GC number / nucleotide.

Sample Name	Clean Reads	Clean bases	Read length (bp)	Q20 (%)	CG (%)
L8 a	466 865 970	70 029 895 500	150	98.06%	27.36%
L8 b	467 240 236	70 086 035 400	150	97.96%	27.08%
L8 c	432 492 428	64 873 864 200	150	97.86%	27.98%
L1 a	466 685 804	70 002 870 600	150	97.92%	26.15%
L1 b	466 931 508	70 039 726 200	150	97.89%	26.30%
L1 c	467 018 992	70 052 848 800	150	97.89%	26.54%

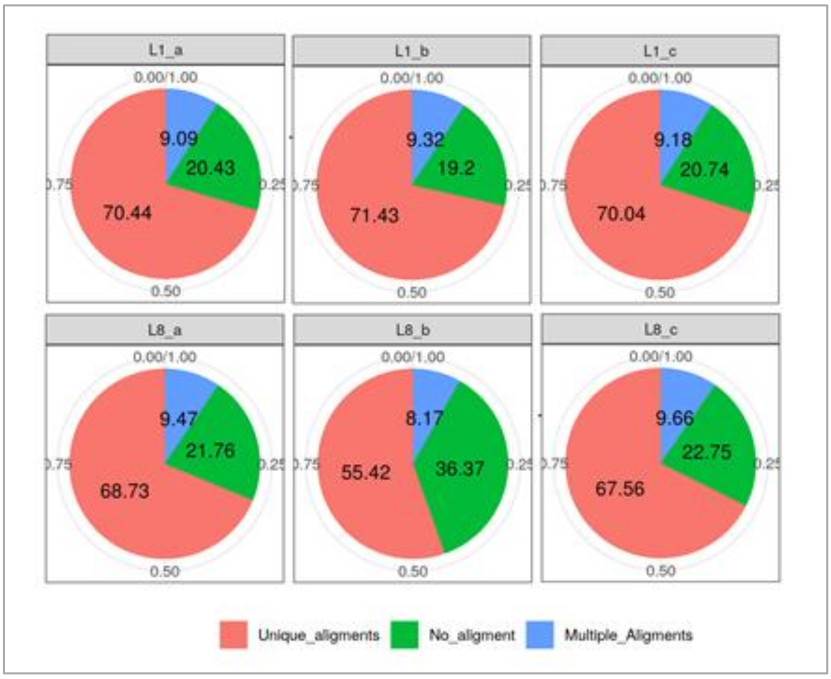

Figure S1. Alignment with the reference genome. Each panel showed the percentage of alignment with the reference genome in each sample.

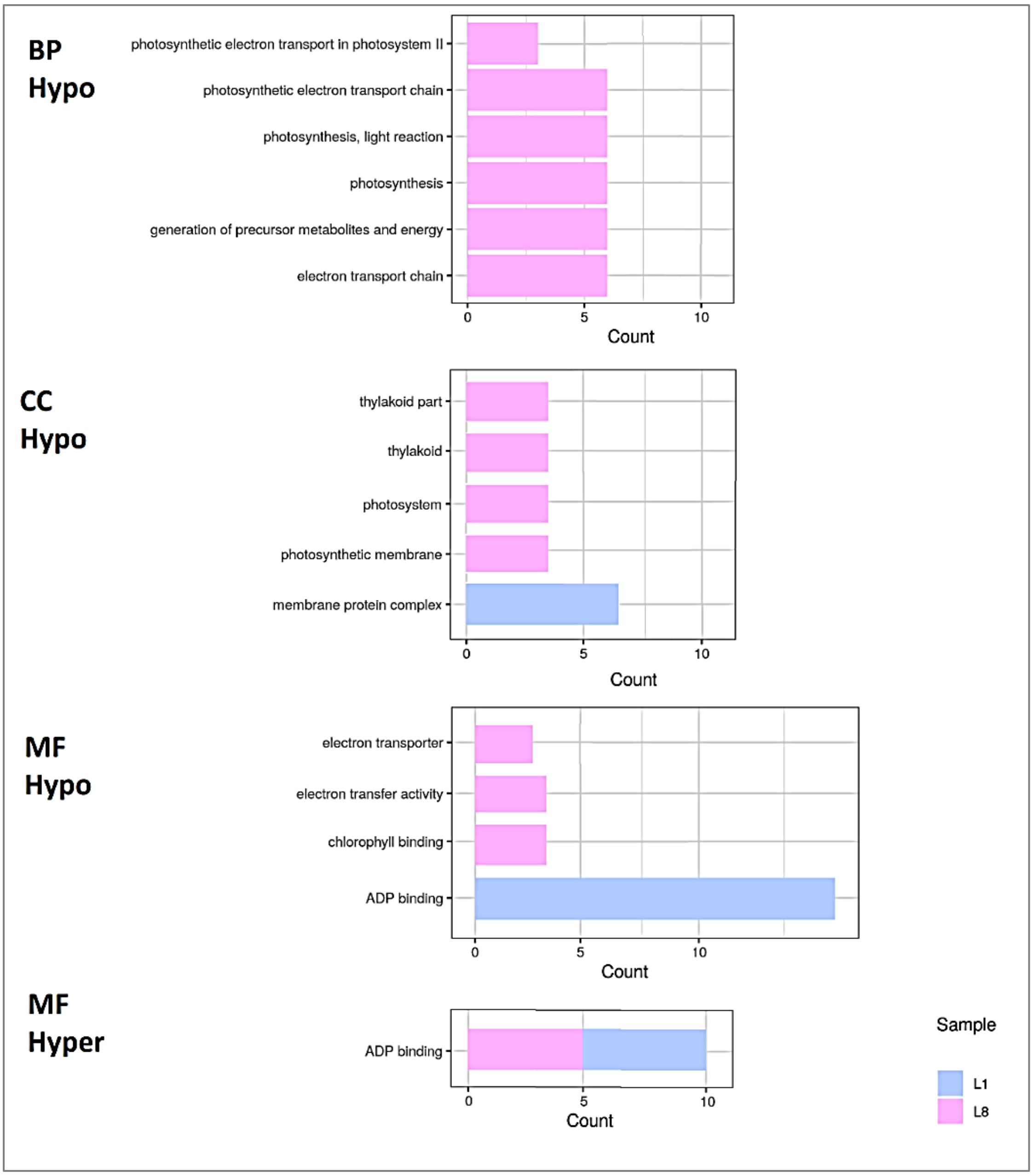

Figure S2. Enrichment analysis of gene ontology terms of the biological process, cellular components and molecular function categories of the annotated genes based on the DmC in CG context.

Table S2. Regions in exons hypermethylated in L8 transgenic line.

CONTEXT CG				
**ID**	**meth.diff**	***p* value**	**q value**	**Note**
Nitab4.5_0001211g0230.1	96	2.44E-14	1.20E-10	Xyloglucan fucosyltransferase
Nitab4.5_0002427g0050.1	91.11111111	2.67E-16	2.39E-12	Unknown
Nitab4.5_0000072g0010.1	88.96969697	8.14E-14	3.31E-10	Unknown
Nitab4.5_0010569g0010.1	88.2962963	2.21E-12	5.46E-09	Unknown
Nitab4.5_0002942g0020.1	87.31884058	7.04E-13	2.06E-09	Transposase, MuDR, plant
Nitab4.5_0009613g0030.1	82.05882353	6.03E-11	9.13E-08	Nucleic acid-binding, OB-fold
Nitab4.5_0003497g0020.1	81.66214995	7.76E-13	2.24E-09	Ribosomal protein L18e/L15P, Ribosomal protein L18e
Nitab4.5_0000284g0020.1	73.92344498	2.63E-11	4.44E-08	Unknown
Nitab4.5_0002302g0060.1	71.8426501	1.63E-07	7.47E-05	Pentatricopeptide repeat, Glycoside hydrolase, family 5, conserved site, Tetratricopeptide-like helical
Nitab4.5_0002214g0140.1	70.66365008	2.12E-10	2.71E-07	Armadillo-type fold, Armadillo-like helical
Nitab4.5_0000719g0050.1	69.72624799	7.62E-08	3.97E-05	Small GTPase superfamily, P-loop containing nucleoside triphosphate hydrolase
Nitab4.5_0001863g0050.1	69.12878788	2.19E-12	5.42E-09	F-box domain, 4Fe-4S ferredoxin, iron-sulphur binding, conserved site
Nitab4.5_0007168g0010.1	68.76923077	2.35E-07	0.000100769	Zinc finger, C2H2
Nitab4.5_0000927g0070.1	67.85714286	1.10E-06	0.000357505	Unknown
Nitab4.5_0000444g0060.1	66.95652174	1.17E-06	0.000377069	Ribonuclease H domain, Ribonuclease H-like domain
Nitab4.5_0004402g0010.1	65.95238095	2.54E-07	0.000107576	Ribonuclease H-like domain

Nitab4.5_0001290g0080.1	65.814393 94	7.12E-09	5.55E-06	Kri1-like, C-terminal, KRR1 interacting protein 1
Nitab4.5_0000503g0010.1	65.272727 27	2.48E-06	0.000689841	Terpenoid synthase, Terpene synthase, N-terminal domain, Terpenoid cyclases/protein prenyltransferase alpha-alpha toroid
Nitab4.5_0000794g0120.1	64.478114 48	3.73E-07	0.00014791	Peptidase S8, subtilisin-related, Peptidase S8/S53 domain, Proteinase inhibitor I9, Peptidase S8, subtilisin, Ser-active site
Nitab4.5_0002120g0040.1	64.467766 12	1.25E-06	0.000398713	Unknown
Nitab4.5_0003691g0010.1	63.636363 64	7.99E-11	1.17E-07	Unknown
Nitab4.5_0009369g0010.1	63.461538 46	7.25E-09	5.63E-06	Unknown
Nitab4.5_0006691g0020.1	63.214285 71	3.00E-10	3.64E-07	Pentatricopeptide repeat, Tetratricopeptide-like helical
Nitab4.5_0001333g0010.1	62.962962 96	7.29E-08	3.83E-05	Pentatricopeptide repeat
Nitab4.5_0010324g0020.1	61.904761 9	4.22E-07	0.000163285	Unknown
Nitab4.5_0006953g0010.1	61.666666 67	1.02E-05	0.00216261	Unknown
Nitab4.5_0000271g0290.1	59.340659 34	1.50E-06	0.000461952	Unknown
Nitab4.5_0001521g0030.1	59.150579 15	2.75E-08	1.69E-05	Unknown
Nitab4.5_0004395g0030.1	59.047619 05	1.56E-05	0.003063646	Pentatricopeptide repeat, Tetratricopeptide-like helical
Nitab4.5_0004533g0020.1	58.816425 12	4.68E-08	2.65E-05	Unknown
Nitab4.5_0005722g0010.1	57.575757 58	4.55E-05	0.007221479	UDP-glucuronosyl/UDP-glucosyltransferase
Nitab4.5_0002634g0010.1	57.481481 48	1.84E-05	0.00349808	Unknown
Nitab4.5_0000271g0290.1	57.400257 4	3.45E-06	0.00090776	Unknown
Nitab4.5_0011637g0010.1	57.105263	3.98E-08	2.31E-05	Pentatricopeptide repeat, Tetratricopeptide-like helical

	16			
Nitab4.5_0000692g0100.1	56.66666667	1.72E-08	1.15E-05	ABC transporter, conserved site, ABC transporter type 1, transmembrane domain, AAA+ ATPase domain, P-loop containing nucleoside triphosphate hydrolase, Domain of unknown function DUF4283, ABC transporter-like
Nitab4.5_0000261g0260.1	56.52680653	8.77E-06	0.001922411	EGF-like calcium-binding domain, Wall-associated receptor kinase galacturonan-binding domain, Serine/threonine- / dual specificity protein kinase, catalytic domain, Protein kinase domain, Protein kinase-like domain, Epidermal growth factor-like domain, Protein kinase, ATP binding site, EGF-like calcium-binding, conserved site, Concanavalin A-like lectin/glucanase, subgroup, Serine/threonine-protein kinase, active site, EGF-type aspartate/asparagine hydroxylation site
Nitab4.5_0009728g0020.1	56.4893617	9.54E-09	7.13E-06	Unknown
Nitab4.5_0000965g0060.1	56.26566416	1.10E-05	0.002295958	Bifunctional inhibitor/plant lipid transfer protein/seed storage helical domain
Nitab4.5_0010096g0010.1	56.01415094	4.52E-10	5.18E-07	Armadillo, Leucine-rich repeat, Armadillo-type fold, Leucine-rich repeat, typical subtype, Acyl transferase/acyl hydrolase/lysophospholipase, Patatin/Phospholipase A2-related, Armadillo-like helical
Nitab4.5_0000159g0040.1	55.55555556	4.75E-07	0.000179571	Multi antimicrobial extrusion protein
Nitab4.5_0001333g0010.1	54.628331	1.15E-06	0.000372228	Pentatricopeptide repeat
Nitab4.5_0000692g0180.1	54.4099976	2.68E-13	9.14E-10	Pentatricopeptide repeat, Tetratricopeptide-like helical
Nitab4.5_0000188g0180.1	54.38596491	1.79E-07	8.05E-05	RNA-binding protein Lupus La, Winged helix-turn-helix DNA-binding domain
Nitab4.5_0001232g0030.1	54.33715221	2.49E-06	0.000693138	Unknown
Nitab4.5_0006854g0010.1	53.97897898	1.28E-09	1.28E-06	DNA photolyase, N-terminal, Rossmann-like alpha/beta/alpha sandwich fold, DNA photolyase, FAD-binding/Cryptochrome, C-terminal
Nitab4.5_0005831g0030.1	53.8277512	2.67E-06	0.000735462	Unknown
Nitab4.5_0000902g0110.1	51.9047619	5.03E-05	0.007803969	Unknown
Nitab4.5_0001106g0120.1	51.70454545	1.59E-07	7.32E-05	Kelch repeat type 1, Galactose oxidase, beta-propeller, Development/cell death domain
Nitab4.5_0000783g0080.1	50	5.14E-11	7.91E-08	Major facilitator superfamily domain, General substrate transporter, Sugar transporter,

				conserved site, Major facilitator superfamily domain, general substrate transporter, Sugar/inositol transporter
Nitab4.5_0000883g0160.1	50	1.96E-07	8.68E-05	Cytochrome P450, Cytochrome P450, E-class, group I, Cytochrome P450, conserved site
Nitab4.5_0004068g0030.1	48.8372093	2.45E-07	0.000104342	Terpenoid synthase, Terpenoid cyclases/protein prenyltransferase alpha-alpha toroid, Terpene synthase, metal-binding domain, Terpene synthase, N-terminal domain
Nitab4.5_0000188g0180.1	48.28375286	8.91E-06	0.001948287	RNA-binding protein Lupus La, Winged helix-turn-helix DNA-binding domain
Nitab4.5_0000015g0390.1	48.27586207	1.73E-05	0.003331775	Unknown
Nitab4.5_0000503g0010.1	47.82608696	3.62E-05	0.006032882	Terpenoid synthase, Terpene synthase, N-terminal domain, Terpenoid cyclases/protein prenyltransferase alpha-alpha toroid
Nitab4.5_0001124g0030.1	47.78554779	2.23E-05	0.004082804	Unknown
Nitab4.5_0003726g0020.1	46.51162791	2.35E-06	0.000661278	SGNH hydrolase-type esterase domain, Lipase, GDSL
Nitab4.5_0005453g0010.1	46.51162791	1.13E-11	2.16E-08	Membrane attack complex component/perforin (MACPF) domain
Nitab4.5_0002413g0020.1	44.96969697	2.05E-05	0.003823565	Unknown
Nitab4.5_0001232g0030.1	44.7826087	2.09E-05	0.003895144	Unknown
Nitab4.5_0000692g0180.1	44.11764706	8.41E-13	2.41E-09	Pentatricopeptide repeat, Tetratricopeptide-like helical
Nitab4.5_0000120g0020.1	44.1025641	3.54E-06	0.000929705	Unknown
Nitab4.5_0000902g0080.1	43.97233202	1.26E-05	0.002573681	Unknown
Nitab4.5_0016571g0010.1	43.48484848	6.65E-09	5.25E-06	Helicase, superfamily 1/2, ATP-binding domain, DNA/RNA helicase, DEAD/DEAH box type, N-terminal, RNA helicase, DEAD-box type, Q motif, RNA helicase, ATP-dependent, DEAD-box, conserved site, P-loop containing nucleoside triphosphate hydrolase
Nitab4.5_0001296g0110.1	42.96296296	1.12E-05	0.00233957	Armadillo-like helical, Armadillo-type fold
Nitab4.5_0007406g0020.1	42.85714286	2.67E-06	0.000735462	Cytochrome P450, Cytochrome P450, E-class, group I
Nitab4.5_0002418g0070.1	42.736842	2.84E-06	0.000772055	NADH:ubiquinone oxidoreductase, subunit 1/F420H2 oxidoreductase subunit H

	11			
Nitab4.5_0001875g0010.1	42	1.82E-09	1.76E-06	P-loop containing nucleoside triphosphate hydrolase, NB-ARC, Disease resistance protein
Nitab4.5_0008990g0010.1	41.93548387	2.96E-05	0.005135734	Pentatricopeptide repeat, Tetratricopeptide-like helical
Nitab4.5_0000957g0050.1	41.81818182	5.46E-10	6.13E-07	Pentatricopeptide repeat
Nitab4.5_0007380g0010.1	41.70820871	3.83E-08	2.23E-05	Tetraspanin, Tetraspanin, conserved site, Tetraspanin/Peripherin
Nitab4.5_0001935g0010.1	41.66666667	7.94E-06	0.001776113	Clathrin adaptor, phosphoinositide-binding, GAT-like, ENTH/VHS, Epsin-like, N-terminal, AP180 N-terminal homology (ANTH) domain
Nitab4.5_0002793g0050.1	41.66666667	6.16E-05	0.009197096	RWP-RK domain, Phox/Bem1p
Nitab4.5_0010353g0010.1	41.57754011	5.04E-05	0.007816458	Nucleic acid-binding, OB-fold, Replication factor A, C-terminal
Nitab4.5_0000441g0160.1	40.90909091	3.07E-05	0.005300123	Nuclear protein DGCR14
Nitab4.5_0007174g0010.1	39.9259651	1.41E-07	6.65E-05	Unknown
Nitab4.5_0005485g0050.1	39.65744401	3.73E-08	2.18E-05	Unknown
Nitab4.5_0004506g0010.1	38.4717608	4.84E-05	0.007576555	Transcription factor MYC/MYB N-terminal, Myc-type, basic helix-loop-helix (bHLH) domain
Nitab4.5_0000628g0110.1	38.0952381	1.59E-05	0.003105714	UDP-glucuronosyl/UDP-glucosyltransferase
Nitab4.5_0001497g0130.1	37.88156288	1.71E-06	0.000512656	Thiolase-like, FAE1/Type III polyketide synthase-like protein, Thiolase-like, subgroup, Very-long-chain 3-ketoacyl-CoA synthase, 3-Oxoacyl-[acyl-carrier-protein (ACP)] synthase III C-terminal
Nitab4.5_0003953g0030.1	37.5	4.16E-05	0.006713427	Unknown
Nitab4.5_0013414g0010.1	35.58208955	3.85E-05	0.006326298	Serine/threonine- / dual specificity protein kinase, catalytic domain, Protein kinase domain, Serine/threonine-protein kinase, active site, Protein kinase-like domain, Protein kinase, ATP binding site
Nitab4.5_0000945g0010.1	35.29411765	3.77E-05	0.006232129	Protein kinase domain, DNA repair metallo-beta-lactamase, Concanavalin A-like lectin/glucanase, subgroup, Beta-lactamase-like, Bulb-type lectin domain, Apple-like, Protein kinase, ATP binding site, S-locus glycoprotein, Protein kinase-like domain, PAN-2 domain, Serine/threonine- / dual specificity protein kinase, catalytic domain,

				Serine/threonine-protein kinase, active site
Nitab4.5_0007794g0010.1	35.294117 65	4.37E-06	0.001102869	Unknown
Nitab4.5_0009433g0020.1	35.273318 72	2.58E-09	2.34E-06	Mannose-binding lectin, P-loop containing nucleoside triphosphate hydrolase, NB-ARC
Nitab4.5_0007732g0010.1	34.615384 62	2.43E-05	0.004359186	H/ACA ribonucleoprotein complex, subunit Gar1/Naf1, Translation protein, beta-barrel domain
Nitab4.5_0000548g0080.1	34.482758 62	1.15E-05	0.002379481	Ribonuclease H-like domain
Nitab4.5_0005485g0020.1	34.212641 95	1.21E-08	8.57E-06	Mannose-binding lectin, Disease resistance protein, P-loop containing nucleoside triphosphate hydrolase, NB-ARC
Nitab4.5_0000194g0090.1	33.981964 88	1.61E-05	0.003141351	Zinc finger, RING/FYVE/PHD-type, Zinc finger, RING-type
Nitab4.5_0000194g0090.1	33.097657 98	1.43E-05	0.002853137	Zinc finger, RING/FYVE/PHD-type, Zinc finger, RING-type
Nitab4.5_0001107g0060.1	32.786885 25	6.01E-05	0.009029503	Epsin domain, N-terminal, ENTH/VHS, Epsin-like, N-terminal
Nitab4.5_0003180g0070.1	31.578947 37	5.04E-15	3.10E-11	Mannose-binding lectin, NB-ARC, P-loop containing nucleoside triphosphate hydrolase, Disease resistance protein
Nitab4.5_0022438g0010.1	31.087811 27	4.70E-08	2.66E-05	ABC transporter, transmembrane domain, ABC transporter type 1, transmembrane domain
Nitab4.5_0000194g0100.1	30.959752 32	2.59E-06	0.000717083	Unknown
Nitab4.5_0000194g0090.1	30.640589 57	1.47E-05	0.002928855	Zinc finger, RING/FYVE/PHD-type, Zinc finger, RING-type
Nitab4.5_0002784g0030.1	30.029476 79	1.73E-05	0.00332749	Tetratricopeptide-like helical, Sel1-like
Nitab4.5_0000692g0030.1	30	1.28E-06	0.000406221	Glutaredoxin, Thioredoxin-like fold
Nitab4.5_0001088g0040.1	29.744346 12	6.07E-05	0.009100036	Armadillo-like helical, Armadillo-type fold, HEAT, type 2
Nitab4.5_0007057g0010.1	28.579350 12	1.88E-06	0.000553405	HIP116, Rad5p N-terminal, VRR-NUC domain, Zinc finger, Rad18-type putative
Nitab4.5_0001927g0060.1	28.571428 57	1.04E-05	0.002192043	Protein of unknown function DUF866, eukaryotic
Nitab4.5_0005485g0050.1	27.832756	1.79E-06	0.000530638	Unknown

	06			
Nitab4.5_0005149g0070.1	27.777777 78	3.89E-05	0.00638368	Unknown
Nitab4.5_0005485g0020.1	27.461139 9	1.96E-14	9.87E-11	Mannose-binding lectin, Disease resistance protein, P-loop containing nucleoside triphosphate hydrolase, NB-ARC
Nitab4.5_0002983g0030.1	27.362110 31	4.65E-05	0.0073371	Unknown
Nitab4.5_0009433g0010.1	26.811829 7	6.58E-08	3.52E-05	NB-ARC, Mannose-binding lectin, Disease resistance protein, P-loop containing nucleoside triphosphate hydrolase
Nitab4.5_0009433g0010.1	26.681812 44	1.02E-07	5.07E-05	NB-ARC, Mannose-binding lectin, Disease resistance protein, P-loop containing nucleoside triphosphate hydrolase
Nitab4.5_0012631g0010.1	25.529388 48	1.82E-05	0.00347979	COBRA, plant
Nitab4.5_0003180g0070.1	25.220070 42	1.73E-05	0.003334976	Mannose-binding lectin, NB-ARC, P-loop containing nucleoside triphosphate hydrolase, Disease resistance protein
**CONTEXT CHG**				
**ID**	**meth.diff**	***p* value**	**q value**	**Note**
Nitab4.5_0008833g0010	100	2.40662E-17	3.00526E-12	Protein-tyrosine phosphatase, active site, Dual specificity phosphatase, subgroup, catalytic domain, Dual specificity phosphatase, catalytic domain, Dual specificity phosphatase, Villin/Gelsolin, Protein-tyrosine/Dual specificity phosphatase
Nitab4.5_0001341g0060	95.83	1.26672E-14	5.84582E-10	Helicase, C-terminal, P-loop containing nucleoside triphosphate hydrolase, GUCT, RNA helicase, DEAD-box type, Q motif, Helicase, superfamily 1/2, ATP-binding domain, DNA/RNA helicase, DEAD/DEAH box type, N-terminal
Nitab4.5_0008752g0030	92	1.67569E-12	3.30649E-08	mRNA splicing factor, Cwf21
Nitab4.5_0009887g0010	91.304347 83	5.01271E-13	1.28209E-08	Armadillo-type fold, Zinc finger, N-recognin, Zinc finger, N-recognin, metazoa, E3 ubiquitin ligase, UBR4
Nitab4.5_0011681g0040	90.740740 74	5.57915E-16	4.22993E-11	Unknown
Nitab4.5_0006489g0010	89.130434 78	2.69485E-19	6.73037E-14	Glycosyl transferase, family 2
Nitab4.5_0001408g0030	88.428571 43	1.23211E-12	2.6965E-08	DNA/RNA-binding domain, Est1-type, Telomerase activating protein Est1, Tetratricopeptide-like helical
Nitab4.5_0007645g0010	88	7.56871E-13	1.79524E-08	NLI interacting factor, HAD-like domain

Nitab4.5_0004829g0060	87.5	5.99834E-11	6.70195E-07	Double-stranded RNA-binding domain, Ribonuclease III domain
Nitab4.5_0002171g0130	87.5	3.12319E-11	3.89554E-07	DNA-directed RNA polymerase, phage-type, DNA-directed RNA polymerase, helix hairpin domain
Nitab4.5_0000687g0010	87.5	5.81669E-16	4.33266E-11	Parallel beta-helix repeat, Pectin lyase fold/virulence factor, Glycoside hydrolase, family 28, Pectin lyase fold
Nitab4.5_0005586g0040	86.95652174	4.08305E-15	2.22251E-10	Calponin homology domain
Nitab4.5_0002393g0030	86.77419355	1.63814E-15	1.02281E-10	Unknown
Nitab4.5_0005627g0040	86.66666667	8.32732E-14	2.806E-09	Tetratricopeptide, MLP1/MLP2-like
Nitab4.5_0002262g0080	86.36363636	2.13197E-11	2.76813E-07	Zinc finger, RING-type, Zinc finger, RING/FYVE/PHD-type
Nitab4.5_0001769g0090	86.20689655	1.15526E-11	1.64045E-07	Glycosyl transferase, family 14
Nitab4.5_0002072g0020	85.71428571	5.34024E-12	8.78809E-08	Unknown
Nitab4.5_0001522g0090	85.71428571	2.81597E-12	5.0876E-08	ELM2 domain, ARID/BRIGHT DNA-binding domain, Homeodomain-like, Myb-like domain
Nitab4.5_0006577g0010	85.71428571	1.46034E-13	4.52572E-09	Mitochodrial transcription termination factor-related
Nitab4.5_0004530g0080	85.71428571	1.00448E-13	3.23088E-09	WEB family
Nitab4.5_0000743g0190	84.61538462	5.75179E-12	9.29142E-08	Pentatricopeptide repeat, Tetratricopeptide-like helical
Nitab4.5_0005627g0040	84.61538462	1.09474E-12	2.45925E-08	Tetratricopeptide, MLP1/MLP2-like
Nitab4.5_0004582g0060	83.57963875	2.21729E-10	1.98609E-06	Stomatin, Band 7 protein
Nitab4.5_0000575g0010	82.75862069	1.34239E-10	1.32545E-06	Signal transduction response regulator, receiver domain, CCT domain, CheY-like superfamily
Nitab4.5_0000502g0190	82.60869565	4.40965E-10	3.46708E-06	NAD(P)-binding domain
Nitab4.5_0001295g0200	82.551319	1.28836E-10	1.28405E-	RNA recognition motif domain, Nucleotide-binding, alpha-beta plait

	65		06	
Nitab4.5_0000687g0090	82.213438 74	2.73098E-09	1.58836E-05	Phosphoesterase domain, Serine/threonine-specific protein phosphatase/bis(5-nucleosyl)-tetraphosphatase
Nitab4.5_0009327g0010	81.962864 72	5.95358E-11	6.66949E-07	2-oxoglutarate dehydrogenase, E1 component, Transketolase-like, pyrimidine-binding domain, Dehydrogenase, E1 component
Nitab4.5_0011089g0020	81.547619 05	1.74783E-10	1.64636E-06	Anaphase-promoting complex subunit 1
Nitab4.5_0000096g0200	80.952380 95	1.77429E-12	3.45559E-08	Protein of unknown function DUF1997
Nitab4.5_0000962g0120	80.769230 77	2.30186E-11	2.96154E-07	Signal transduction histidine kinase, phosphotransfer (Hpt) domain
Nitab4.5_0000220g0110	80	3.11281E-19	7.13606E-14	Pentatricopeptide repeat, Tetratricopeptide-like helical
Nitab4.5_0005086g0010	79.467084 64	1.16712E-09	7.86552E-06	BSD
Nitab4.5_0002930g0010	79.166666 67	2.70063E-10	2.32109E-06	Unknown
Nitab4.5_0008080g0010	79.160419 79	3.56153E-10	2.91918E-06	Glycoside hydrolase, family 1, Glycoside hydrolase, catalytic domain, Glycoside hydrolase, superfamily, Glycoside hydrolase, family 1, active site
Nitab4.5_0002923g0020	78.571428 57	7.76472E-15	3.83337E-10	Domain of unknown function DUF2428, death-receptor-like, Armadillo-type fold
Nitab4.5_0000757g0100	78.476190 48	1.44369E-08	6.19145E-05	Protein kinase domain, Protein kinase-like domain
Nitab4.5_0000499g0030	78.363636 36	8.49254E-09	4.04228E-05	Transcription factor IIS, N-terminal, Bromo adjacent homology (BAH) domain, Transcription elongation factor, TFIIS/CRSP70, N-terminal, sub-type
Nitab4.5_0000202g0170	78.260869 57	2.11998E-10	1.91917E-06	Proteasome component (PCI) domain, Eukaryotic translation initiation factor 3 subunit A
Nitab4.5_0005354g0070	78.125	4.53585E-11	5.3199E-07	RNA recognition motif domain, Nucleotide-binding, alpha-beta plait
Nitab4.5_0004886g0010	77.823920 27	3.35933E-12	5.93173E-08	Zinc finger, CCCH-type, K Homology domain, type 1, K Homology domain
Nitab4.5_0000003g0130	77.5	2.33494E-11	2.986E-07	Exportin-1/Importin-beta-like, Importin-beta, N-terminal domain, Armadillo-like helical, Armadillo-type fold
Nitab4.5_0004792g0040	77.419354 84	9.58991E-10	6.69675E-06	Protein of unknown function DUF1997

Nitab4.5_0000101g0340	76.92307692	6.4665E-10	4.81668E-06	SANT/Myb domain, Homeodomain-like, Myb domain
Nitab4.5_0001168g0100	76.92307692	2.6241E-13	7.28186E-09	Ubinuclein/Yemanuclein, Hpc2-related domain
Nitab4.5_0000008g0430	76.48648649	4.10598E-09	2.23213E-05	Probable transposase, Ptta/En/Spm, plant
Nitab4.5_0006489g0010	76.47849462	1.00957E-09	6.9697E-06	Glycosyl transferase, family 2
Nitab4.5_0000360g0130	76	2.11176E-09	1.27721E-05	Helicase, C-terminal, P-loop containing nucleoside triphosphate hydrolase, RNA helicase, DEAD-box type, Q motif, Helicase, superfamily 1/2, ATP-binding domain, DNA/RNA helicase, DEAD/DEAH box type, N-terminal
Nitab4.5_0000101g0340	76	7.50835E-10	5.43075E-06	SANT/Myb domain, Homeodomain-like, Myb domain
Nitab4.5_0018824g0010	75.67567568	5.20328E-11	5.92707E-07	Endoplasmic reticulum vesicle transporter, C-terminal
Nitab4.5_0005029g0050	75	3.45839E-08	0.000127129	ENTH/VHS, Epsin domain, N-terminal, Epsin-like, N-terminal
Nitab4.5_0004184g0030	74.07407407	4.59745E-11	5.37729E-07	Protein of unknown function DUF4370
Nitab4.5_0000582g0120	73.91304348	3.11531E-09	1.76673E-05	MT-A70-like
Nitab4.5_0000033g0340	73.88888889	1.00512E-07	0.000304821	DhaL domain, Dak kinase
Nitab4.5_0006698g0010	73.78516624	4.30592E-09	2.30831E-05	NADH pyrophosphatase-like, N-terminal, NUDIX hydrolase, conserved site, NUDIX hydrolase domain-like, NUDIX hydrolase domain, Zinc ribbon, NADH pyrophosphatase, NUDIX hydrolase
Nitab4.5_0015167g0010	73.52941176	1.65974E-11	2.22297E-07	AP2/ERF domain, DNA-binding domain
Nitab4.5_0004839g0020	73.36182336	1.00536E-08	4.63464E-05	DNA-directed RNA polymerase III largest subunit, RNA polymerase Rpb1, domain 3, RNA polymerase, N-terminal, RNA polymerase Rpb1, domain 4, RNA polymerase Rpb1, domain 5, RNA polymerase Rpb1, domain 1, RNA polymerase, alpha subunit
Nitab4.5_0001299g0010	72.95238095	1.22428E-07	0.000356514	Zinc finger, CCCH-type, Zinc finger, RING-type, Zinc finger, RING-type, conserved site, Zinc finger, RING/FYVE/PHD-type, Putative E3 ubiquitin-protein ligase, makorin-related
Nitab4.5_0002855g0030	72.77419355	1.44484E-09	9.40861E-06	HCaRG

Nitab4.5_0000508g0210	72.72727273	2.7925E-09	1.61529E-05	Monooxygenase, FAD-binding, Aromatic-ring hydroxylase-like
Nitab4.5_0002385g0020	72.61904762	1.96071E-07	0.000523564	WD40 repeat, WD40/YVTN repeat-like-containing domain, WD40-repeat-containing domain
Nitab4.5_0000052g0390	72.57525084	2.43852E-09	1.446E-05	Unknown
Nitab4.5_0013539g0010	72	3.6723E-09	2.04079E-05	Unknown
Nitab4.5_0009617g0010	72	1.46854E-09	9.50219E-06	Pentatricopeptide repeat, Mitochondrial carrier domain, Methionine synthase, vitamin-B12 independent, Tetratricopeptide-like helical
Nitab4.5_0000375g0110	71.42857143	1.42061E-08	6.1314E-05	Tetratricopeptide-like helical, NSF attachment protein, Tetratricopeptide repeat, Malate dehydrogenase, active site, Tetratricopeptide repeat-containing domain
Nitab4.5_0008768g0060	71.21710526	3.83425E-10	3.09491E-06	Unknown
Nitab4.5_0000971g0140	70.96774194	8.2941E-10	5.92838E-06	von Willebrand factor, type A, Zinc finger, Sec23/Sec24-type, Sec23/Sec24, helical domain, Gelsolin domain, Sec23/Sec24, trunk domain
Nitab4.5_0000008g0570	70.83333333	7.40944E-09	3.61177E-05	Mitochondrial brown fat uncoupling protein, Mitochondrial carrier domain, Mitochondrial substrate/solute carrier
Nitab4.5_0003725g0080	70.83333333	2.02461E-09	1.23505E-05	P-loop containing nucleoside triphosphate hydrolase, Uncharacterised protein family, ATP binding
Nitab4.5_0000021g0400	70.47619048	1.61327E-07	0.000445352	Unknown
Nitab4.5_0002641g0070	70.37037037	1.86717E-08	7.65942E-05	Pentatricopeptide repeat, Tetratricopeptide-like helical
Nitab4.5_0003449g0060	70.37037037	4.39877E-10	3.46708E-06	Zinc finger, FYVE/PHD-type, Bromo adjacent homology (BAH) domain, Zinc finger, PHD-finger, Zinc finger, RING/FYVE/PHD-type, Zinc finger, PHD-type, conserved site, Zinc finger, PHD-type
Nitab4.5_0001198g0240	70	2.84888E-09	1.63675E-05	Alternative oxidase
Nitab4.5_0000430g0150	69.76744186	1.84718E-10	1.71237E-06	Ankyrin repeat, Potassium channel, voltage-dependent, EAG/ELK/ERG, Ion transport domain, Ankyrin repeat-containing domain, Potassium channel, plant-type
Nitab4.5_0008327g0020	69.56521739	2.36005E-08	9.28652E-05	Zinc finger, PHD-finger, Zinc finger, FYVE/PHD-type, Zinc finger, RING-type, Zinc finger, RING/FYVE/PHD-type, Zinc finger, PHD-type
Nitab4.5_0000861g0060	69.56521739	1.52855E-08	6.49631E-05	Zinc finger, RanBP2-type

Nitab4.5_0000065g0080	69.43164363	1.02021E-07	0.000308515	FMN-binding split barrel
Nitab4.5_0005579g0020	69.41391941	5.55033E-07	0.001181809	Reverse transcriptase zinc-binding domain
Nitab4.5_0004928g0060	69.15113872	5.46799E-07	0.001171326	Signal transduction histidine kinase, hybrid-type, ethylene sensor, GAF domain, Signal transduction histidine kinase, homodimeric domain, Signal transduction response regulator, receiver domain, Histidine kinase-like ATPase, ATP-binding domain, CheY-like superfamily, Signal transduction histidine kinase, core
Nitab4.5_0001242g0120	68.96551724	1.9243E-11	2.5452E-07	DALR anticodon binding, Arginine-tRNA ligase, class Ia, Rossmann-like alpha/beta/alpha sandwich fold, Aminoacyl-tRNA synthetase, class 1a, anticodon-binding, Arginyl-tRNA synthetase, class Ia, core
Nitab4.5_0000012g0040	68.95238095	8.73698E-07	0.001683074	Protein of unknown function DUF810, Mammalian uncoordinated homology 13, domain 2, Munc13 homology 1
Nitab4.5_0000348g0160	68.77470356	1.24318E-06	0.002228036	DHR-1 domain, Dedicator of cytokinesis
Nitab4.5_0000738g0020	68.67636868	2.59671E-10	2.25921E-06	START domain, Domain of unknown function DUF1336, Pleckstrin homology-like domain, Pleckstrin homology domain, START-like domain
Nitab4.5_0000687g0090	68.57142857	1.43499E-09	9.3732E-06	Phosphoesterase domain, Serine/threonine-specific protein phosphatase/bis(5-nucleosyl)-tetraphosphatase
Nitab4.5_0005166g0010	68.18181818	1.15407E-07	0.000339796	AWS, Post-SET domain, SET domain, Zinc finger, CW-type
Nitab4.5_0005914g0010	68	4.13987E-07	0.000951823	Protein of unknown function DUF962
Nitab4.5_0002626g0030	68	3.95926E-08	0.000142828	Protein phosphatase 2A, regulatory B subunit, B56, Armadillo-type fold
Nitab4.5_0000031g0060	67.85714286	1.12241E-07	0.000333481	Anticodon-binding domain
Nitab4.5_0007329g0050	67.74193548	2.36801E-08	9.30923E-05	WD40-repeat-containing domain, WD40/YVTN repeat-like-containing domain, WD40 repeat, conserved site, WD40 repeat
Nitab4.5_0000179g0020	67.56756757	2.5529E-10	2.22565E-06	Probable transposase, Ptta/En/Spm, plant
Nitab4.5_0001564g0060	67.26190476	5.35244E-07	0.001152387	Helicase/SANT-associated, DNA binding, HAS subgroup, Homeodomain-like, Myb-like domain, SANT/Myb domain
Nitab4.5_0000771g0050	67.07452725	3.96129E-08	0.000142828	Mitochondrial inner membrane translocase subunit Tim17/Tim22/Tim23/peroxisomal protein PMP24, Mitochondrial inner membrane translocase complex, subunit Tim17

Nitab4.5_0004309g0010	67	4.81789E-07	0.0010653 91	RNA recognition motif domain, Ataxin-2, C-terminal, Nucleotide-binding, alpha-beta plait
Nitab4.5_0001299g0010	66.771159 87	5.54039E-07	0.0011818 09	Zinc finger, CCCH-type, Zinc finger, RING-type, Zinc finger, RING-type, conserved site, Zinc finger, RING/FYVE/PHD-type, Putative E3 ubiquitin-protein ligase, makorin-related
Nitab4.5_0012929g0020	66.666666 67	3.04393E-07	0.0007474 7	HECT
Nitab4.5_0001010g0020	66.666666 67	1.0338E-07	0.0003109 83	DNA primase, large subunit, eukaryotic, DNA primase large subunit, eukaryotic/archaeal
Nitab4.5_0006967g0030	66.666666 67	6.99822E-08	0.0002278 58	Nucleotide-sugar transporter
Nitab4.5_0000179g0020	66.666666 67	6.73913E-08	0.0002211 17	Probable transposase, Ptta/En/Spm, plant
Nitab4.5_0000667g0050	66.666666 67	4.5528E-09	2.41625E-05	Longin-like domain, Coatomer delta subunit, Clathrin adaptor, mu subunit, C-terminal
Nitab4.5_0000575g0050	66.666666 67	1.52101E-09	9.78454E-06	Rab-GTPase-TBC domain
Nitab4.5_0000507g0040	66.666666 67	1.58672E-11	2.13189E-07	Unknown
Nitab4.5_0003127g0010	66.505636 07	5.17562E-09	2.67653E-05	DhaL domain, Dak kinase
Nitab4.5_0001854g0020	66.485507 25	4.76697E-07	0.0010596 5	Unknown
Nitab4.5_0002930g0010	65.909090 91	1.66746E-09	1.05508E-05	Unknown
Nitab4.5_0001160g0070	65.878378 38	1.41832E-07	0.0004011 88	Lipid-binding serum glycoprotein, N-terminal, Lipid-binding serum glycoprotein, C-terminal, Bactericidal permeability-increasing protein, alpha/beta domain
Nitab4.5_0001913g0040	65.833333 33	4.31883E-07	0.0009821 45	Unknown
Nitab4.5_0000179g0100	65.517241 38	1.08401E-08	4.90139E-05	Basic-leucine zipper domain, G-box binding, MFMR
Nitab4.5_0010794g0010	65.517241 38	4.53356E-09	2.41207E-05	Nucleic acid-binding, OB-fold, Translation elongation factor P, Elongation factor P, C-terminal, Translation protein SH3-like domain, Ribosomal protein L2 domain 2, Translation elongation factor, KOW-like, Translation elongation factor P/YeiP, conserved site, Translation elongation factor P/YeiP, Translation elongation factor P/YeiP, central
Nitab4.5_0000914g0020	65.517241	2.84994E-10	2.42974E-	EF-hand domain, EF-hand domain pair, EF-Hand 1, calcium-binding site

	38		06	
Nitab4.5_0000101g0340	65.517241 38	1.98035E-10	1.8043E-0 6	SANT/Myb domain, Homeodomain-like, Myb domain
Nitab4.5_0000019g0280	65.384615 38	4.99091E-08	0.0001705 51	WD40 repeat, WD40-repeat-containing domain, WD40/YVTN repeat-like-containing domain, WD40 repeat, conserved site, LisH dimerisation motif, CTLH, C-terminal LisH motif
Nitab4.5_0005927g0020	65.384615 38	4.99091E-08	0.0001705 51	YjeF C-terminal domain, carbohydrate kinase-related, ADP/ATP-dependent (S)-NAD(P)H-hydrate dehydratase
Nitab4.5_0010831g0060	65.333333 33	2.47733E-07	0.0006283 22	Unknown
Nitab4.5_0003616g0010	65.217391 3	4.00564E-06	0.0054474 43	Malic oxidoreductase, Malic enzyme, NAD-binding, NAD(P)-binding domain
Nitab4.5_0000002g0030	65.217391 3	3.49331E-06	0.0049209 27	N-acetyltransferase B complex, non-catalytic subunit, Tetratricopeptide-like helical
Nitab4.5_0001198g0240	65	2.06177E-08	8.34484E-05	Alternative oxidase
Nitab4.5_0000818g0090	64.789915 97	1.0272E-09	7.07991E-06	Alpha 1,4-glycosyltransferase domain, Glycosyltransferase, DXD sugar-binding motif
Nitab4.5_0001416g0010	64.335664 34	5.42494E-07	0.0011626 89	Myb domain, plants, SANT/Myb domain, MYB-CC type transcription factor, LHEQLE-containing domain, Homeodomain-like, Myb domain
Nitab4.5_0003328g0060	64.285714 29	7.15925E-09	3.49785E-05	Uncharacterised protein family UPF0118
Nitab4.5_0000409g0140	64.137214 14	1.43904E-07	0.0004048 91	Unknown
Nitab4.5_0000240g0180	64.102564 1	5.61277E-08	0.0001892 8	Urease, alpha subunit, Urease, gamma/gamma-beta subunit, Metal-dependent hydrolase, composite domain, Urease, alpha subunit, C-terminal, Urease, beta subunit, Amidohydrolase 1, Urease alpha-subunit, N-terminal, Urease, alpha subunit, conserved site
Nitab4.5_0000496g0180	64.010989 01	1.53362E-07	0.0004278 14	Protein kinase C-like, phorbol ester/diacylglycerol binding, Diacylglycerol kinase, accessory domain, ATP-NAD kinase-like domain, Diacylglycerol kinase, catalytic domain
Nitab4.5_0002782g0090	63.965517 24	1.56978E-06	0.0026702 22	Protein of unknown function DUF295
Nitab4.5_0000309g0320	63.763066 2	5.88305E-08	0.0001974 54	Tetratricopeptide repeat, Sel1-like, Tetratricopeptide-like helical, Tetratricopeptide TPR1, Tetratricopeptide repeat-containing domain
Nitab4.5_0006878g0030	63.636363 64	3.07497E-07	0.0007529 14	Zinc finger, PHD-finger, Zinc finger, FYVE/PHD-type, Zinc finger, PHD-type, DDT domain, Zinc finger, RING/FYVE/PHD-type, DDT domain superfamily, DDT domain, subgroup,

				Zinc finger, PHD-type, conserved site
Nitab4.5_0000143g0130	63.63636364	9.81787E-08	0.000298811	Armadillo-type fold, Mo25-like, Armadillo-like helical
Nitab4.5_0001589g0050	63.63636364	6.84545E-08	0.000223914	Zinc finger, GRF-type
Nitab4.5_0000879g0090	63.63636364	2.81173E-08	0.000107162	Alpha-amylase, C-terminal beta-sheet, Glycoside hydrolase, family 13, Glycosyl hydrolase, family 13, subfamily, catalytic domain, Glycoside hydrolase, superfamily, Glycosyl hydrolase, family 13, all-beta, Glycoside hydrolase, catalytic domain, Glycosyl hydrolase, family 13, catalytic domain
Nitab4.5_0007918g0010	63.54166667	5.1791E-07	0.00112304	Bromodomain transcription factor, Transcription factor TFIID, subunit 8, C-terminal, Histone-fold
Nitab4.5_0002944g0010	63.05263158	1.76645E-07	0.000478615	Membrane insertase YidC/Oxa1, C-terminal, Membrane insertase OXA1/ALB3/YidC
Nitab4.5_0000152g0130	62.96296296	3.56132E-07	0.000847557	Armadillo-type fold, Protein of unknown function DUF3437, Armadillo-like helical
Nitab4.5_0003502g0120	62.96296296	9.32489E-08	0.000287726	GDP-fucose protein O-fucosyltransferase, O-fucosyltransferase, plant
Nitab4.5_0003566g0030	62.5	5.33495E-06	0.006753372	Cyclophilin-like peptidyl-prolyl cis-trans isomerase domain
Nitab4.5_0002180g0050	62.11981567	4.10859E-08	0.000146712	Unknown
Nitab4.5_0000126g0070	62.06896552	8.95645E-08	0.00027838	Unknown
Nitab4.5_0005625g0030	61.9047619	1.26993E-07	0.000365126	Peptidase M41, RNA recognition motif domain, Peptidase, FtsH, ATPase, AAA-type, core, AAA+ ATPase domain, Nucleotide-binding, alpha-beta plait, P-loop containing nucleoside triphosphate hydrolase, ATPase, AAA-type, conserved site
Nitab4.5_0002441g0070	61.75	2.98951E-07	0.000737517	Protein kinase domain, Protein kinase, ATP binding site, Protein kinase-like domain
Nitab4.5_0000287g0280	61.66666667	2.4683E-07	0.000626405	AGC-kinase, C-terminal, Protein kinase domain, Serine/threonine-protein kinase, active site, Protein kinase, C-terminal, Protein kinase, ATP binding site, Protein kinase-like domain, Serine/threonine- / dual specificity protein kinase, catalytic domain
Nitab4.5_0001159g0020	61.53846154	4.11987E-07	0.0009491	Alpha-ketoglutarate-dependent dioxygenase AlkB-like, Nucleotide-binding, alpha-beta plait, Oxoglutarate/iron-dependent dioxygenase, RNA recognition motif domain
Nitab4.5_0002087g0070	61.29032258	5.65728E-07	0.001201567	Diphthamide synthesis DHP2, eukaryotic, Diphthamide synthesis, DPH1/DPH2

Nitab4.5_0000335g0130	60.975609 76	7.36593E-10	5.34595E-06	HAD-like domain, Double-stranded RNA-binding domain, NLI interacting factor
Nitab4.5_0004222g0040	60.869565 22	3.89591E-07	0.0009093 47	Transcription factor, MADS-box, Transcription factor, K-box
Nitab4.5_0002760g0060	60.857142 86	1.3046E-06	0.0023128 47	START-like domain, Streptomyces cyclase/dehydrase
Nitab4.5_0007329g0050	60.839160 84	2.9753E-06	0.0043499 87	WD40-repeat-containing domain, WD40/YVTN repeat-like-containing domain, WD40 repeat, conserved site, WD40 repeat
Nitab4.5_0000738g0020	60.714285 71	5.14918E-07	0.0011199 68	START domain, Domain of unknown function DUF1336, Pleckstrin homology-like domain, Pleckstrin homology domain, START-like domain
Nitab4.5_0008661g0010	60.714285 71	4.90849E-08	0.0001690 7	DNA primase, small subunit, DNA primase, small subunit, eukaryotic/archaeal
Nitab4.5_0001829g0050	60.714285 71	7.75573E-09	3.74617E-05	Nucleotidyl transferase domain
Nitab4.5_0001946g0020	60.606060 61	1.06462E-06	0.0019764 27	Snf7
Nitab4.5_0004679g0090	60.606060 61	1.30994E-07	0.0003750 29	Mono-/di-acylglycerol lipase, N-terminal
Nitab4.5_0000464g0090	60.056202 33	7.74597E-12	1.1997E-07	Pentatricopeptide repeat, Tetratricopeptide-like helical
Nitab4.5_0002137g0100	60	1.94206E-06	0.0031603 99	Unknown
Nitab4.5_0002426g0090	60	3.46099E-07	0.0008297 26	Zinc finger, RING/FYVE/PHD-type, Zinc finger, RING-type
Nitab4.5_0000301g0070	60	2.35932E-07	0.0006019 88	G-box binding, MFMR, Basic-leucine zipper domain
Nitab4.5_0000667g0010.1	60	7.90449E-08	0.0002517 66	Regulator of nonsense-mediated decay, UPF3, Nucleotide-binding, alpha-beta plait
Nitab4.5_0001872g0020	60	3.97753E-08	0.0001432 36	Pentatricopeptide repeat
Nitab4.5_0001290g0110	60	6.59551E-09	3.25614E-05	Pentatricopeptide repeat, Tetratricopeptide-like helical
Nitab4.5_0000046g0150	59.610389 61	1.53835E-06	0.0026325 89	Protein kinase-like domain, Serine-threonine/tyrosine-protein kinase catalytic domain, Serine/threonine- / dual specificity protein kinase, catalytic domain, Protein kinase domain, Serine/threonine-protein kinase, active site

Nitab4.5_0000179g0020	59.408867	8.39872E-08	0.000265318	Probable transposase, Ptta/En/Spm, plant
Nitab4.5_0000592g0080	59.28853755	6.90921E-06	0.008255948	Protein of unknown function DUF3550/UPF0682
Nitab4.5_0001990g0020	59.25925926	1.07709E-06	0.001994349	Peptidase M17, leucyl aminopeptidase, C-terminal, Leucine aminopeptidase/peptidase B
Nitab4.5_0008042g0040	59.25925926	2.08749E-07	0.000548884	PAP/25A-associated, D-isomer specific 2-hydroxyacid dehydrogenase, catalytic domain, NAD(P)-binding domain, D-isomer specific 2-hydroxyacid dehydrogenase, NAD-binding
Nitab4.5_0000154g0040	59.25925926	1.08762E-09	7.44797E-06	Unknown
Nitab4.5_0000459g0090	59.15750916	4.9509E-10	3.84986E-06	BEACH domain, PH-BEACH domain, Concanavalin A-like lectin/glucanase, subgroup, Armadillo-like helical, Concanavalin A-like lectin/glucanases superfamily
Nitab4.5_0002055g0150	59.09090909	2.45001E-06	0.003752564	Signal transduction response regulator, receiver domain, Myb domain, plants, Homeodomain-like, Myb domain, SANT/Myb domain, CheY-like superfamily
Nitab4.5_0000347g0180	59.09090909	1.53994E-07	0.000429297	Unknown
Nitab4.5_0004093g0010	59.09090909	1.13209E-07	0.000335419	GNAT domain, Acyl-CoA N-acyltransferase
Nitab4.5_0004093g0010.1:cds	59.09090909	1.13209E-07	0.000335419	character(0)
Nitab4.5_0000162g0030	58.86363636	3.9136E-06	0.005351404	Domain of unknown function DUF1981, Sec7 associated, Armadillo-type fold, Armadillo-like helical
Nitab4.5_0009327g0010	58.70689655	5.16468E-07	0.001121058	2-oxoglutarate dehydrogenase, E1 component, Transketolase-like, pyrimidine-binding domain, Dehydrogenase, E1 component
Nitab4.5_0003773g0070	58.66935484	1.46698E-06	0.002533155	Vacuolar protein sorting-associated protein 54
Nitab4.5_0012788g0010	58.62068966	6.46821E-07	0.001337018	Basic-leucine zipper domain, Transcription factor TGA like domain
Nitab4.5_0007204g0020	58.62068966	2.79041E-07	0.000695676	Protein kinase domain, Ankyrin repeat-containing domain, Ankyrin repeat, Serine/threonine-protein kinase CTR1/EDR1, Protein kinase-like domain, Integrin-linked protein kinase
Nitab4.5_0005154g0090	58.46702317	4.42995E-07	0.001001678	Aspartyl/glutamyl-tRNA(Asn/Gln) amidotransferase, B subunit, Asn/Gln amidotransferase, Aspartyl/glutamyl-tRNA amidotransferase subunit B-related, Aspartyl/Glutamyl-tRNA(Gln) amidotransferase, subunit B/E, catalytic, Glutamyl-tRNA(Gln) amidotransferase, subunit B, conserved site,

				Aspartyl/glutamyl-tRNA(Asn/Gln) amidotransferase, subunit B /E
Nitab4.5_0007184g0030	58.36148649	3.67181E-07	0.000866566	Unknown
Nitab4.5_0003586g0040	58.33333333	1.70344E-07	0.000465101	Unknown
Nitab4.5_0001215g0210	58.13953488	3.47816E-10	2.86745E-06	P-type ATPase, A domain, HAD-like domain, P-type ATPase, cytoplasmic domain N, Cation-transporting P-type ATPase, Cation-transporting P-type ATPase, subfamily IV, P-type ATPase, phosphorylation site
Nitab4.5_0000470g0060	58.06451613	5.98117E-07	0.001256652	SMCs flexible hinge, RecF/RecN/SMC, N-terminal, P-loop containing nucleoside triphosphate hydrolase, Structural maintenance of chromosomes protein
Nitab4.5_0000301g0070	57.97720798	3.78806E-06	0.005228578	G-box binding, MFMR, Basic-leucine zipper domain
Nitab4.5_0001678g0020	57.89473684	1.24097E-09	8.29736E-06	Ribonucleoprotein LSM domain, eukaryotic/archaea-type, Small nuclear ribonucleoprotein E, Ribonucleoprotein LSM domain, Like-Sm (LSM) domain
Nitab4.5_0003893g0060	57.69230769	2.82538E-07	0.000701922	Phosphoribosylglycinamide synthetase, conserved site, Phosphoribosylglycinamide synthetase, ATP-grasp (A) domain, Phosphoribosylglycinamide synthetase, C-domain, Rudiment single hybrid motif, ATP-grasp fold, subdomain 2
Nitab4.5_0006332g0050	57.69230769	1.9784E-07	0.00052654	Unknown
Nitab4.5_0005692g0010	57.69230769	6.32401E-09	3.15512E-05	GroEL-like apical domain, GroEL-like equatorial domain, TCP-1-like chaperonin intermediate domain, Chaperonin Cpn60/TCP-1, T-complex protein 1, delta subunit, Chaperonin TCP-1, conserved site, Chaperone tailless complex polypeptide 1 (TCP-1)
Nitab4.5_0005295g0020	57.68518519	1.48974E-06	0.002563859	SWAP/Surp, RNA recognition motif domain, SAP domain, Nucleotide-binding, alpha-beta plait, mRNA splicing factor, Cwf21
Nitab4.5_0001208g0090	57.14285714	2.41097E-06	0.00371556	Transcription elongation factor 1
Nitab4.5_0000317g0220	57.14285714	1.72285E-06	0.002875307	Zinc finger, CW-type, SET domain, Post-SET domain, AWS
Nitab4.5_0001642g0040	57.14285714	3.65094E-07	0.000863082	DNA glycosylase, Helix-turn-helix, base-excision DNA repair, C-terminal, HhH-GPD domain
Nitab4.5_0001307g0020	57.14285714	4.34656E-08	0.000153787	Unknown
Nitab4.5_0001288g0140	57.14285714	9.04398E-12	1.33792E-07	Histidine triad (HIT) protein, HIT-like domain, Histidine triad, conserved site
Nitab4.5_0000186g0030	56.923076	4.58453E-06	0.0060478	Unknown

	92		74	
Nitab4.5_0012713g0010	56.86956522	3.31355E-07	0.000800128	RNA polymerase I associated factor, A49-like
Nitab4.5_0023875g0010	56.48148148	1.82503E-09	1.14114E-05	DNA binding domain, putative, B3/B4 tRNA-binding domain
Nitab4.5_0000137g0020	56.25	6.0904E-07	0.00127569	Unknown
Nitab4.5_0003952g0040	56	5.73648E-07	0.001212324	Pentatricopeptide repeat, Tetratricopeptide-like helical
Nitab4.5_0001542g0120	55.88235294	7.19553E-08	0.000233744	RNA polymerase, beta subunit, conserved site, RNA polymerase Rpb2, domain 3, RNA polymerase I, Rpa2 specific, RNA polymerase Rpb2, domain 2, DNA-directed RNA polymerase, subunit 2, RNA polymerase Rpb2, domain 7, DNA-directed RNA polymerase, subunit 2, domain 6, RNA polymerase Rpb2, OB-fold, RNA polymerase, beta subunit, protrusion
Nitab4.5_0000757g0030	55.59772296	2.15735E-07	0.000562313	Unknown
Nitab4.5_0001296g0150	55.56756757	6.29589E-06	0.007696721	Unknown
Nitab4.5_0000902g0070	55.55555556	8.86993E-08	0.000276095	GRAM domain
Nitab4.5_0001507g0030	55.55555556	6.25988E-08	0.000207692	Pentatricopeptide repeat, Tetratricopeptide-like helical
Nitab4.5_0005056g0110	55.55555556	6.25988E-08	0.000207692	Anticodon-binding, Aminoacyl-tRNA synthetase, class II, Prolyl-tRNA synthetase, class II, Proline-tRNA ligase, class II, C-terminal, Proline-tRNA ligase, class IIa, archaeal-type, Proline-tRNA ligase, class IIa, Aminoacyl-tRNA synthetase, class II (G/ H/ P/ S), conserved domain
Nitab4.5_0000143g0130	55.45454545	2.25965E-06	0.003551581	Armadillo-type fold, Mo25-like, Armadillo-like helical
Nitab4.5_0007130g0010	55.28735632	7.51516E-07	0.001498001	Cornichon
Nitab4.5_0000794g0160	55.22340084	1.36916E-12	2.87777E-08	Winged helix-turn-helix DNA-binding domain, B-block binding subunit of TFIIIC
Nitab4.5_0002130g0070	55.09259259	7.30393E-06	0.008616443	UDP-glucuronosyl/UDP-glucosyltransferase
Nitab4.5_0000916g0140	54.974358	1.26176E-06	0.0022518	AP180 N-terminal homology (ANTH) domain, ENTH/VHS, Clathrin adaptor,

	97		36	phosphoinositide-binding, GAT-like, Epsin-like, N-terminal
Nitab4.5_0001737g0100	54.838709 68	4.8151E-07	0.0010653 29	Thioredoxin-like fold
Nitab4.5_0017161g0010	54.796511 63	7.04716E-07	0.0014268 19	Cytochrome P450, Cytochrome P450, E-class, group IV
Nitab4.5_0001678g0020	54.626532 89	3.13447E-06	0.0045250 45	Ribonucleoprotein LSM domain, eukaryotic/archaea-type, Small nuclear ribonucleoprotein E, Ribonucleoprotein LSM domain, Like-Sm (LSM) domain
Nitab4.5_0002171g0130	54.545454 55	2.11425E-06	0.0033657 79	DNA-directed RNA polymerase, phage-type, DNA-directed RNA polymerase, helix hairpin domain
Nitab4.5_0000445g0150	54.545454 55	1.14484E-06	0.0020890 74	Transcription antitermination protein NusG, N-terminal domain, Ribosomal protein L24/L26, conserved site, Transcription elongation factor Spt5, NGN domain, KOW, Transcription elongation factor Spt5, Translation protein SH3-like domain, Spt5 transcription elongation factor, N-terminal
Nitab4.5_0000143g0130	54.471544 72	9.1986E-08	0.0002846 56	Armadillo-type fold, Mo25-like, Armadillo-like helical
Nitab4.5_0005039g0030	54.457364 34	1.22523E-08	5.43575E-05	Diaminopimelate epimerase, DapF, Diaminopimelate epimerase, active site
Nitab4.5_0001571g0080	54.166666 67	1.64717E-06	0.0027729 75	Zinc finger, U1-type
Nitab4.5_0000001g0360	54.166666 67	3.50793E-07	0.0008390 86	CCR4-Not complex component, Not N-terminal domain
Nitab4.5_0004308g0070	54.166666 67	1.14833E-07	0.0003390 49	Ribosomal RNA large subunit methyltransferase RlmN/Cfr, Dual-specificity RNA methyltransferase RlmN, Radical SAM, Aldolase-type TIM barrel
Nitab4.5_0013229g0030	53.888888 89	1.87989E-07	0.0005051 59	Arsenical pump ATPase, ArsA/GET3, P-loop containing nucleoside triphosphate hydrolase, Anion-transporting ATPase-like domain
Nitab4.5_0000348g0160	53.571428 57	3.20273E-06	0.0046063 54	DHR-1 domain, Dedicator of cytokinesis
Nitab4.5_0008572g0030	53.455284 55	2.93928E-09	1.68186E-05	Histidinol dehydrogenase, Adipose-regulatory protein, Seipin, Aldehyde/histidinol dehydrogenase, Histidinol dehydrogenase, conserved site
Nitab4.5_0002972g0010	53.295932 68	3.57799E-06	0.0050020 44	Unknown
Nitab4.5_0000439g0080	53.260073 26	2.60157E-07	0.0006534 21	SNARE associated Golgi protein
Nitab4.5_0000332g0140	53.125	1.3617E-08	5.9175E-0 5	Yippee/Mis18

Nitab4.5_0001542g0050	53.10344828	2.83487E-06	0.004195236	tRNA (cytidine/uridine-2'-O-)-methyltransferase, tRNA/rRNA methyltransferase, SpoU
Nitab4.5_0006489g0010	52.8881823	4.96508E-07	0.001089992	Glycosyl transferase, family 2
Nitab4.5_0001542g0120	52.77777778	1.70453E-07	0.000465101	RNA polymerase, beta subunit, conserved site, RNA polymerase Rpb2, domain 3, RNA polymerase I, Rpa2 specific, RNA polymerase Rpb2, domain 2, DNA-directed RNA polymerase, subunit 2, RNA polymerase Rpb2, domain 7, DNA-directed RNA polymerase, subunit 2, domain 6, RNA polymerase Rpb2, OB-fold, RNA polymerase, beta subunit, protrusion
Nitab4.5_0000610g0200	52.43407708	1.32923E-06	0.002346601	Pentatricopeptide repeat, Tetratricopeptide-like helical
Nitab4.5_0003979g0020	52.38095238	2.61211E-06	0.003946742	Unknown
Nitab4.5_0005498g0020	52.38095238	1.98539E-06	0.00320877	Cytochrome P450, Cytochrome P450, E-class, group I, Cytochrome P450, conserved site
Nitab4.5_0002670g0010	52.08333333	1.09366E-07	0.00032631	Zinc finger, CCHC-type, Putative 5-3 exonuclease, 5'-3' exoribonuclease, 5'-3' exoribonuclease 2
Nitab4.5_0010612g0010	52	7.12463E-06	0.008451897	JmjC domain, ARID/BRIGHT DNA-binding domain, Transcription factor jumonji, JmjN, Zinc finger, PHD-type, conserved site, Lysine-specific demethylase-like domain, Zinc finger, FYVE/PHD-type, Zinc finger, PHD-finger, Zinc finger, RING/FYVE/PHD-type, Zinc finger, C5HC2-type, Zinc finger, PHD-type
Nitab4.5_0008002g0030	52	5.05766E-06	0.006491383	Acyl-CoA thioesterase
Nitab4.5_0004290g0010	51.85185185	2.04555E-06	0.003278578	P-loop containing nucleoside triphosphate hydrolase, Disease resistance protein, NB-ARC
Nitab4.5_0001307g0020	51.85185185	4.67224E-08	0.000163268	Unknown
Nitab4.5_0009290g0010	51.72413793	4.79273E-06	0.006241927	Aspartic peptidase, Aspartic peptidase domain
Nitab4.5_0000185g0040	51.61290323	1.32737E-06	0.002345313	Myb-like domain, SANT/Myb domain, Homeodomain-like
Nitab4.5_0000794g0160	51.51515152	3.43701E-06	0.004867463	Winged helix-turn-helix DNA-binding domain, B-block binding subunit of TFIIIC
Nitab4.5_0001296g0130	51.51515152	5.33566E-07	0.00114994	tRNA (guanine(9)-N(1))-methyltransferase TRM10, tRNA (guanine-N1-)-methyltransferase, eukaryotic, tRNA (guanine-N1-)-methyltransferase

Nitab4.5_0000794g0160	51.351351 35	1.15312E-06	0.0021012 26	Winged helix-turn-helix DNA-binding domain, B-block binding subunit of TFIIIC
Nitab4.5_0000794g0160.1:cds	51.351351 35	1.15312E-06	0.0021012 26	DNA polymerase A, Ribonuclease H-like domain, DNA-directed DNA polymerase, family A, palm domain, 3'-5' exonuclease domain
Nitab4.5_0000775g0170	50.805931 66	3.11122E-06	0.0045037 3	Unknown
Nitab4.5_0000312g0100	50	6.14E-06	0.0075474 29	DnaJ domain, DnaJ domain, conserved site, DnaJ-like protein C11, C-terminal
Nitab4.5_0002004g0080	50	6.14E-06	0.0075474 29	Zinc knuckle CX2CX4HX4C
Nitab4.5_0000381g0210	50	5.50598E-06	0.0069164 79	F-box domain
Nitab4.5_0000399g0050	50	5.33495E-06	0.0067533 72	SNARE associated Golgi protein
Nitab4.5_0002287g0060	50	4.29506E-06	0.0057616 74	Rab3 GTPase-activating protein catalytic subunit
Nitab4.5_0000301g0070	50	2.93693E-06	0.0043215 13	G-box binding, MFMR, Basic-leucine zipper domain
Nitab4.5_0001376g0070	50	2.29087E-06	0.0035904 16	Arsenical pump ATPase, ArsA/GET3, Anion-transporting ATPase-like domain, P-loop containing nucleoside triphosphate hydrolase
Nitab4.5_0003485g0040	50	1.73701E-06	0.0028921 18	Unknown
Nitab4.5_0000341g0060	50	1.20617E-06	0.0021764 02	Protein kinase-like domain, UbiB domain
Nitab4.5_0008312g0030	50	4.66064E-07	0.0010414 66	Dihydrolipoamide dehydrogenase, FAD/NAD-linked reductase, dimerisation domain, Pyridine nucleotide-disulphide oxidoreductase, dimerisation domain, Pyridine nucleotide-disulphide oxidoreductase, NAD-binding domain, FAD-dependent pyridine nucleotide-disulphide oxidoreductase, Pyridine nucleotide-disulphide oxidoreductase, class I, active site, Pyridine nucleotide-disulphide oxidoreductase, FAD/NAD(P)-binding domain
Nitab4.5_0000794g0160	50	1.21069E-07	0.0003535 26	Winged helix-turn-helix DNA-binding domain, B-block binding subunit of TFIIIC
Nitab4.5_0015019g0010	50	1.86441E-09	1.16238E-05	Ribosomal protein S23/S29, mitochondrial
Nitab4.5_0007821g0030	50	1.01525E-10	1.05908E-06	Pentatricopeptide repeat, Tetratricopeptide-like helical

Nitab4.5_0000573g0090	49.23076923	1.26034E-07	0.000363635	Dcp1-like decapping, Pleckstrin homology-like domain
Nitab4.5_0002027g0130	49.04880066	4.74445E-07	0.001056303	Protein kinase domain, Serine/threonine- / dual specificity protein kinase, catalytic domain, Serine/threonine-protein kinase, active site, Protein kinase-like domain
Nitab4.5_0000794g0130	49.02834008	1.37382E-07	0.000391401	Basic-leucine zipper domain
Nitab4.5_0000560g0050	48.97959184	1.09694E-06	0.00202492	Magnesium transporter NIPA
Nitab4.5_0000573g0120	48.85521886	5.44919E-08	0.000184791	LETM1-like
Nitab4.5_0003410g0040	48.72563718	1.54202E-06	0.002634723	RNA recognition motif domain, U2 snRNP auxilliary factor, large subunit, splicing factor, Nucleotide-binding, alpha-beta plait
Nitab4.5_0000905g0090	48.57142857	2.2554E-06	0.003546615	Putative S-adenosyl-L-methionine-dependent methyltransferase
Nitab4.5_0003300g0030	48.45528455	1.65553E-06	0.002782646	Unknown
Nitab4.5_0005664g0040	48.28934506	3.54591E-06	0.00497029	P-loop containing nucleoside triphosphate hydrolase, Probable helicase MAGATAMA 3
Nitab4.5_0003454g0010	48.14814815	2.43663E-06	0.003742866	Aldolase-type TIM barrel, N-(5'phosphoribosyl) anthranilate isomerase (PRAI) like domain, Ribulose-phosphate binding barrel
Nitab4.5_0014983g0010	48.07692308	3.75189E-08	0.000136266	Malic enzyme, NAD-binding, Malic enzyme, N-terminal, Malic enzyme, conserved site, Malic oxidoreductase, NAD(P)-binding domain
Nitab4.5_0001672g0080	48	3.42236E-06	0.004858062	PPPDE putative peptidase domain
Nitab4.5_0001807g0100	47.83910997	1.25801E-06	0.002246242	SecY subunit domain, SecY/SEC61-alpha family
Nitab4.5_0002811g0050	47.82608696	6.45988E-06	0.007858727	ELM2 domain
Nitab4.5_0003555g0090	47.72727273	4.8752E-06	0.006316391	Protein kinase domain, Serine/threonine-protein kinase, active site, Serine/threonine- / dual specificity protein kinase, catalytic domain, Protein kinase, ATP binding site, Protein kinase-like domain
Nitab4.5_0001486g0040	47.5	1.46383E-06	0.002529529	Helicase, C-terminal, P-loop containing nucleoside triphosphate hydrolase, Helicase, superfamily 1/2, ATP-binding domain, DNA/RNA helicase, DEAD/DEAH box type, N-terminal
Nitab4.5_0008718g0040	47.5	4.33659E-09	2.32182E-	F-box domain

			05	
Nitab4.5_0012058g0010	47.05882353	8.99071E-09	4.21792E-05	SET domain, PWWP domain, Zinc finger, PHD-type, Zinc finger, PHD-finger, Zinc finger, PHD-type, conserved site, Zinc finger, RING/FYVE/PHD-type, Histone-lysine N-methyltransferase ATX, Zinc finger, FYVE/PHD-type
Nitab4.5_0008209g0030	47.01710471	2.09008E-06	0.003334805	ATPase, F1/V1/A1 complex, alpha/beta subunit, nucleotide-binding domain, P-loop containing nucleoside triphosphate hydrolase, ATPase, alpha/beta subunit, nucleotide-binding domain, active site, ATPase, F1/V1/A1 complex, alpha/beta subunit, C-terminal, ATPase, F1 complex beta subunit/V1 complex, C-terminal
Nitab4.5_0000783g0080	46.81872749	8.22281E-07	0.001606623	Major facilitator superfamily domain, General substrate transporter, Sugar transporter, conserved site, Major facilitator superfamily domain, general substrate transporter, Sugar/inositol transporter
Nitab4.5_0007068g0030	46.42857143	4.49146E-07	0.001011648	Acyl-CoA N-acyltransferase
Nitab4.5_0002666g0010	46.15384615	2.43869E-06	0.003744674	Unknown
Nitab4.5_0011274g0010	46.15384615	3.00058E-08	0.000113747	ATPase, BadF/BadG/BcrA/BcrD type
Nitab4.5_0001700g0130	45.83333333	4.52433E-06	0.005987876	EF-hand domain pair, EF-Hand 1, calcium-binding site, EF-hand domain, Protein kinase, ATP binding site, Serine/threonine- / dual specificity protein kinase, catalytic domain, Protein kinase-like domain, Serine/threonine-protein kinase, active site, Protein kinase domain
Nitab4.5_0002103g0090	45.72727273	1.97851E-06	0.003203751	Unknown
Nitab4.5_0005642g0010	45.55555556	6.40066E-08	0.000211648	Heavy metal-associated domain, HMA, Heavy-metal-associated, conserved site, Cation-transporting P-type ATPase, P-type ATPase, phosphorylation site, HAD-like domain, Cation-transporting P-type ATPase, subfamily IB, P-type ATPase, A domain, P-type ATPase, cytoplasmic domain N
Nitab4.5_0009266g0060	45.45454545	1.6133E-06	0.002728945	Hydantoinase/dihydropyrimidinase, Metal-dependent hydrolase, composite domain
Nitab4.5_0006718g0010	45.28301887	4.3065E-12	7.43263E-08	Unknown
Nitab4.5_0005199g0080	45.16129032	4.36207E-07	0.000990385	Glycosyl transferase, ALG6/ALG8
Nitab4.5_0000061g0120	45.16129032	3.41432E-07	0.000821322	EF-hand domain pair, Mitochondrial carrier protein, EF-Hand 1, calcium-binding site, Mitochondrial carrier domain, Mitochondrial substrate/solute carrier, EF-hand domain

Nitab4.5_0011089g0010	45.09803922	2.95557E-06	0.004337017	Anaphase-promoting complex subunit 1
Nitab4.5_0008057g0030	45	6.50829E-07	0.001343992	Pentatricopeptide repeat, Tetratricopeptide-like helical
Nitab4.5_0001863g0210	45	2.79832E-09	1.61567E-05	GPI inositol-deacylase PGAP1-like
Nitab4.5_0010095g0010	44.82758621	1.74106E-07	0.00047264	EF-Hand 1, calcium-binding site, EPS15 homology (EH), EF-hand domain pair, EF-hand domain
Nitab4.5_0011205g0010	44.44444444	4.23339E-09	2.28385E-05	DnaJ domain, Domain of unknown function DUF4101
Nitab4.5_0020349g0030	44.20180723	6.88993E-08	0.000225022	ATPase, AAA-type, core, P-loop containing nucleoside triphosphate hydrolase
Nitab4.5_0001737g0100	44.11764706	6.84205E-06	0.008199143	Thioredoxin-like fold
Nitab4.5_0000483g0120	44.11764706	5.0509E-06	0.006486629	Peptidase C56, PfpI, ThiJ/PfpI
Nitab4.5_0003680g0090	44.11764706	1.57758E-07	0.000437776	Pentatricopeptide repeat, Tetratricopeptide-like helical
Nitab4.5_0015167g0010	44.11764706	9.87655E-08	0.000300166	AP2/ERF domain, DNA-binding domain
Nitab4.5_0000794g0160	44	3.12874E-11	3.89554E-07	Winged helix-turn-helix DNA-binding domain, B-block binding subunit of TFIIIC
Nitab4.5_0006232g0040	43.58974359	2.89624E-06	0.004273081	RNA recognition motif domain, Nucleotide-binding, alpha-beta plait
Nitab4.5_0015167g0010	43.47826087	6.76862E-06	0.008134108	AP2/ERF domain, DNA-binding domain
Nitab4.5_0003314g0020	43.24324324	2.10785E-06	0.00335919	Armadillo-type fold, Armadillo-like helical
Nitab4.5_0000573g0120	43.18181818	4.49912E-06	0.005967532	LETM1-like
Nitab4.5_0000159g0030	43.1372549	1.17148E-07	0.000343258	Phosphoesterase domain
Nitab4.5_0000033g0060	43.05555556	3.8548E-07	0.000902729	Di-haem cytochrome, transmembrane, Cytochrome b/b6, Cytochrome b/b6-like domain, Cytochrome b/b6, N-terminal
Nitab4.5_0005485g0020	42.941176	1.51048E-10	1.45422E-	Mannose-binding lectin, Disease resistance protein, P-loop containing nucleoside

	47		06	triphosphate hydrolase, NB-ARC
Nitab4.5_0002983g0050	42.940603 7	5.54368E-08	0.0001872 47	Unknown
Nitab4.5_0000902g0070	42.915642 92	8.62159E-06	0.0097172 92	GRAM domain
Nitab4.5_0001294g0020	42.857142 86	5.0831E-06	0.0065067 11	Clp, N-terminal, Double Clp-N motif
Nitab4.5_0000159g0110	42.857142 86	4.02568E-06	0.0054694 36	Peptidase C48, SUMO/Sentrin/Ubl1
Nitab4.5_0013867g0020	42.846661 78	1.02655E-06	0.0019166 44	Ubiquitin-fold modifier 1
Nitab4.5_0001587g0030	42.380952 38	3.95978E-06	0.0053981 91	Translocation protein Sec62
Nitab4.5_0004484g0010	42.307692 31	7.50402E-06	0.0088108 72	Rossmann-like alpha/beta/alpha sandwich fold, Valine-tRNA ligase, Valyl/Leucyl/Isoleucyl-tRNA synthetase, class I, anticodon-binding, Aminoacyl-tRNA synthetase, class 1a, anticodon-binding, Valyl/Leucyl/Isoleucyl-tRNA synthetase, editing domain, Aminoacyl-tRNA synthetase, class Ia, Aminoacyl-tRNA synthetase, class I, conserved site
Nitab4.5_0009771g0010	42.105263 16	8.81378E-08	0.0002747 5	ATP-NAD kinase-like domain, Diacylglycerol kinase, catalytic domain, Diacylglycerol kinase, accessory domain, Diacylglycerol kinase, plant
Nitab4.5_0011089g0010	41.981747 07	1.17775E-06	0.0021388 31	Anaphase-promoting complex subunit 1
Nitab4.5_0005257g0020	41.860465 12	7.23026E-06	0.0085533 03	Pentatricopeptide repeat, Tetratricopeptide-like helical
Nitab4.5_0000595g0050	41.666666 67	5.30715E-06	0.0067342 44	Unknown
Nitab4.5_0008572g0030	40.983606 56	8.16641E-09	3.90895E-05	Histidinol dehydrogenase, Adipose-regulatory protein, Seipin, Aldehyde/histidinol dehydrogenase, Histidinol dehydrogenase, conserved site
Nitab4.5_0000451g0180	40.625	7.85476E-06	0.0091177 39	GRIM-19
Nitab4.5_0000159g0070	40.476190 48	2.70836E-06	0.0040432 49	Protein-tyrosine phosphatase, catalytic, Protein-tyrosine phosphatase, receptor/non-receptor type, Protein-tyrosine/Dual specificity phosphatase, Protein-tyrosine phosphatase, active site
Nitab4.5_0001432g0030	40.352504 64	2.07854E-06	0.0033192 51	DNA mismatch repair protein MutS, core, DNA mismatch repair protein MutS, C-terminal, P-loop containing nucleoside triphosphate hydrolase, DNA mismatch repair protein, MSH2,

				DNA mismatch repair protein MutS, clamp, DNA mismatch repair protein MutS, connector domain, DNA mismatch repair protein MutS-like, N-terminal
Nitab4.5_0002784g0030	40	7.31225E-06	0.00861952	Tetratricopeptide-like helical, Sel1-like
Nitab4.5_0005485g0020	39.8989899	2.72895E-08	0.00010457	Mannose-binding lectin, Disease resistance protein, P-loop containing nucleoside triphosphate hydrolase, NB-ARC
Nitab4.5_0011089g0010	39.61113229	1.48269E-06	0.002556903	Anaphase-promoting complex subunit 1
Nitab4.5_0000827g0060	39.47368421	2.27523E-08	9.04497E-05	AMP-dependent synthetase/ligase, AMP-binding, conserved site
Nitab4.5_0005056g0110	39.02439024	6.25043E-06	0.007658792	Anticodon-binding, Aminoacyl-tRNA synthetase, class II, Prolyl-tRNA synthetase, class II, Proline-tRNA ligase, class II, C-terminal, Proline-tRNA ligase, class IIa, archaeal-type, Proline-tRNA ligase, class IIa, Aminoacyl-tRNA synthetase, class II (G/ H/ P/ S), conserved domain
Nitab4.5_0000051g0040	38.55799373	2.30059E-06	0.003600332	Exostosin-like
Nitab4.5_0012408g0010	38.46153846	6.00737E-06	0.007414451	Pentatricopeptide repeat, Tetratricopeptide-like helical
Nitab4.5_0013753g0010	38.34880313	3.16064E-07	0.000768131	C1-like, DC1, Zinc finger, RING/FYVE/PHD-type
Nitab4.5_0000747g0010	38.27848101	6.29103E-06	0.00769521	Photosystem I PsaA/PsaB
Nitab4.5_0001568g0150	37.93103448	6.84412E-06	0.008199311	Uncharacterised protein family UPF0061
Nitab4.5_0000998g0050	37.83783784	8.36752E-08	0.000264727	Protein phosphatase 2C (PP2C)-like domain, Protein phosphatase 2C
Nitab4.5_0002351g0020	37.5	1.88445E-06	0.003093921	Serine/threonine- / dual specificity protein kinase, catalytic domain, Protein kinase-like domain, UbiB domain
Nitab4.5_0000159g0030	37.31343284	3.72595E-09	2.0625E-05	Phosphoesterase domain
Nitab4.5_0000747g0010	36.44927536	1.54208E-06	0.002634723	Photosystem I PsaA/PsaB
Nitab4.5_0000914g0150	36.36363636	2.23459E-06	0.003519105	AATF leucine zipper-containing domain, Apoptosis-antagonizing transcription factor, C-terminal
Nitab4.5_0000688g0290	35.918367	7.64156E-06	0.0089352	SWAP/Surp, Ubiquitin domain, Ubiquitin supergroup, Pre-mRNA splicing factor

	35		96	PRP21-like protein
Nitab4.5_0019126g0010	35.731782	6.59911E-06	0.007982363	Myc-type, basic helix-loop-helix (bHLH) domain
Nitab4.5_0002496g0040	35.71428571	6.64922E-06	0.00802247	Zinc finger, RING/FYVE/PHD-type, Zinc finger, PHD-type, Zinc finger, FYVE/PHD-type, Zinc finger, PHD-type, conserved site, Zinc finger, PHD-finger
Nitab4.5_0000159g0110	35.38461538	1.26391E-06	0.00225397	Peptidase C48, SUMO/Sentrin/Ubl1
Nitab4.5_0003956g0020	34.7826087	1.11197E-06	0.002043776	Bromo adjacent homology (BAH) domain
Nitab4.5_0009771g0010	34.375	1.6077E-06	0.002723687	ATP-NAD kinase-like domain, Diacylglycerol kinase, catalytic domain, Diacylglycerol kinase, accessory domain, Diacylglycerol kinase, plant
Nitab4.5_0000827g0130	34.28571429	6.83511E-06	0.008193143	Peptidase C2, calpain, large subunit, domain III, Cysteine peptidase, cysteine active site, Concanavalin A-like lectin/glucanases superfamily, Peptidase C2, calpain, catalytic domain, Peptidase C2, calpain, domain III, Concanavalin A-like lectin/glucanase, subgroup, Peptidase C2, calpain family
Nitab4.5_0000827g0130	34.28571429	5.66747E-09	2.88867E-05	Peptidase C2, calpain, large subunit, domain III, Cysteine peptidase, cysteine active site, Concanavalin A-like lectin/glucanases superfamily, Peptidase C2, calpain, catalytic domain, Peptidase C2, calpain, domain III, Concanavalin A-like lectin/glucanase, subgroup, Peptidase C2, calpain family
Nitab4.5_0003376g0040	34.21052632	1.01575E-06	0.001903188	Tetratricopeptide-like helical, Plant specific mitochondrial import receptor subunit TOM20
Nitab4.5_0000573g0120	34.04255319	4.45564E-07	0.001005715	LETM1-like
Nitab4.5_0005485g0020	33.9834668	4.07155E-06	0.005515865	Mannose-binding lectin, Disease resistance protein, P-loop containing nucleoside triphosphate hydrolase, NB-ARC
Nitab4.5_0000573g0160	33.7037037	1.52799E-06	0.00262013	Tetratricopeptide TPR1, Tetratricopeptide repeat, Tetratricopeptide-like helical, Tetratricopeptide repeat-containing domain
Nitab4.5_0003858g0090	32.5	8.41551E-06	0.009559611	RNA recognition motif domain, Nucleotide-binding, alpha-beta plait
Nitab4.5_0005330g0040	32.30769231	3.61754E-07	0.000857094	Nonaspanin (TM9SF)
Nitab4.5_0013867g0020	32.14285714	3.60967E-09	2.01125E-05	Ubiquitin-fold modifier 1
Nitab4.5_0007168g0010	31.57894737	6.84638E-06	0.008199705	Zinc finger, C2H2

Nitab4.5_0013867g0020	31.50776053	4.4151E-08	0.000155822	Ubiquitin-fold modifier 1
Nitab4.5_0007074g0010	31.37254902	5.23694E-08	0.00017802	Protein of unknown function DUF2921
Nitab4.5_0000368g0250	30.90909091	4.98437E-06	0.006426467	Pentatricopeptide repeat, Tetratricopeptide-like helical
Nitab4.5_0013867g0020	29.62962963	2.3382E-06	0.003643072	Ubiquitin-fold modifier 1
Nitab4.5_0003555g0090	28.71809101	1.72946E-06	0.002884898	Protein kinase domain, Serine/threonine-protein kinase, active site, Serine/threonine- / dual specificity protein kinase, catalytic domain, Protein kinase, ATP binding site, Protein kinase-like domain
Nitab4.5_0013867g0020	28.54251012	3.68369E-07	0.000868887	Ubiquitin-fold modifier 1
Nitab4.5_0000573g0120	28.16901408	3.61381E-06	0.005042165	LETM1-like
Nitab4.5_0000707g0040	28.15533981	1.90366E-10	1.75705E-06	WD40 repeat, conserved site, WD40/YVTN repeat-like-containing domain, WD40 repeat, WD40-repeat-containing domain, Small-subunit processome, Utp21
Nitab4.5_0007346g0020	27.06062345	7.0802E-06	0.008420359	Zinc finger, DHHC-type, palmitoyltransferase
Nitab4.5_0002741g0140	26.72043011	6.52556E-06	0.00792048	Translocation protein Sec62
Nitab4.5_0001816g0250	26.5060241	5.4768E-07	0.00117203	Endonuclease/exonuclease/phosphatase, Reverse transcriptase zinc-binding domain
Nitab4.5_0001251g0020	26.31578947	2.43476E-06	0.003741349	Unknown
Nitab4.5_0000827g0100	25.86956522	8.15651E-06	0.009354512	Conserved hypothetical protein CHP01589, plant
Nitab4.5_0020415g0010	25.84033613	5.03785E-07	0.001104743	Ribulose bisphosphate carboxylase, large subunit, C-terminal, P-loop containing nucleoside triphosphate hydrolase, Ribulose bisphosphate carboxylase, large subunit, ferrodoxin-like N-terminal, Uncharacterised protein family Ycf1, ATPase, F1/V1/A1 complex, alpha/beta subunit, nucleotide-binding domain
Nitab4.5_0000573g0120	25.49019608	6.9798E-06	0.008323779	LETM1-like
**CONTEXT CHH**				
**ID**	**meth.diff**	***p* value**	**q value**	**Note**

Nitab4.5_0000363g0220	72.6574501	1.26E-11	6.11E-06	Pentatricopeptide repeat, Tetratricopeptide-like helical
Nitab4.5_0007548g0020	68.3229814	8.40E-08	0.00584196	ATPase, F1 complex, epsilon subunit, mitochondrial
Nitab4.5_0000070g0240	64.516129	1.92E-09	0.00041264	Heat shock protein 70, conserved site, Heat shock protein 70 family

Table S3. Regions in exons hypomethylated in L8 transgenic line.

CONTEXT CG				
**ID**	**meth.diff**	***p* value**	**q value**	**Note**
Nitab4.5_0000160g0100.1	−88.23529412	1.92E-12	4.82E-09	Unknown
Nitab4.5_0007944g0030.1	−87.87878788	1.46E-13	5.54E-10	Unknown
Nitab4.5_0002155g0070.1	−83.88746803	1.36E-11	2.51E-08	Pentatricopeptide repeat, Tetratricopeptide-like helical
Nitab4.5_0000705g0020.1	−80.55555556	1.01E-11	1.98E-08	Protein kinase domain, Serine/threonine- / dual specificity protein kinase, catalytic domain, Legume lectin domain, Protein kinase-like domain, Protein kinase, ATP binding site, Serine/threonine-protein kinase, active site, Concanavalin A-like lectin/glucanase, subgroup, Concanavalin A-like lectin/glucanases superfamily
Nitab4.5_0005150g0010.1	−78.57142857	1.18E-08	8.46E-06	Regulator of chromosome condensation 1/beta-lactamase-inhibitor protein II, Serine-threonine/tyrosine-protein kinase catalytic domain, Protein kinase-like domain, TNFR/NGFR cysteine-rich region, Serine/threonine- / dual specificity protein kinase, catalytic domain, Serine/threonine-protein kinase, active site, Protein kinase domain, Protein kinase, ATP binding site
Nitab4.5_0008224g0030.1	−75.28409091	9.70E-11	1.38E-07	AAA-type ATPase, N-terminal domain, ATPase, AAA-type, core
Nitab4.5_0005949g0020.1	−72.19047619	6.69E-08	3.58E-05	Zinc finger, C3HC4 RING-type, Zinc finger, RING-type, Zinc finger, RING/FYVE/PHD-type
Nitab4.5_0003757g0070.1	−71.91142191	2.05E-09	1.93E-06	Ribosomal protein L34Ae, Protein of unknown function DUF1666
Nitab4.5_0000159g0040.1	−71.3570634	1.21E-11	2.28E-08	Multi antimicrobial extrusion protein
Nitab4.5_0000015g0390.1	−71.30434783	1.49E-07	6.94E-05	Unknown
Nitab4.5_0010230g0010.1	−71.27192982	3.14E-09	2.78E-06	Peptidase S9A, prolyl oligopeptidase, Peptidase S9A, N-terminal domain, Peptidase S9, prolyl oligopeptidase, catalytic domain, Peptidase S9, serine active site
Nitab4.5_0002279g0110.1	−69.4235589	7.07E-08	3.74E-05	Unknown
Nitab4.5_0004679g0010.1	−67.97619048	4.20E-09	3.59E-06	Protein of unknown function DUF573
Nitab4.5_0005472g0050.1	−67.86833856	1.62E-07	7.42E-05	NAC domain
Nitab4.5_0000251g0020.1	−67.5	2.29E-08	1.46E-05	Unknown
Nitab4.5_0000902g0080.1	−67.05882353	3.60E-08	2.12E-05	Unknown
Nitab4.5_0000037g014	−65.85106383	1.63E-10	2.14E-07	EF-Hand 1, calcium-binding site,

0.1				UDP-glucuronosyl/UDP-glucosyltransferase
Nitab4.5_0000261g0170.1	−65.77777778	6.27E-07	0.000225472	Unknown
Nitab4.5_0004881g0070.1	−65.38461538	1.23E-07	5.94E-05	Nucleic acid-binding, OB-fold, Replication factor A, C-terminal
Nitab4.5_0004679g0010.1	−65.14285714	9.81E-09	7.31E-06	Protein of unknown function DUF573
Nitab4.5_0000159g0040.1	−62.74509804	1.74E-11	3.08E-08	Multi antimicrobial extrusion protein
Nitab4.5_0000573g0090.1	−61.7440695	2.30E-13	8.05E-10	Dcp1-like decapping, Pleckstrin homology-like domain
Nitab4.5_0004557g0060.1	−59.77742448	8.49E-08	4.34E-05	WD40 repeat, WD40-repeat-containing domain, WD40/YVTN repeat-like-containing domain
Nitab4.5_0007380g0040.1	−57.69230769	1.12E-08	8.10E-06	Uncharacterised domain Wax2, C-terminal, Fatty acid hydroxylase
Nitab4.5_0009395g0010.1	−57.41935484	3.59E-05	0.005998183	WD40 repeat, WD40-repeat-containing domain, WD40 repeat, conserved site, WD40/YVTN repeat-like-containing domain
Nitab4.5_0013415g0010.1	−57.36170213	4.58E-07	0.000174579	UDP-glucuronosyl/UDP-glucosyltransferase
Nitab4.5_0004864g0050.1	−57.32689211	1.64E-06	0.000496296	Nucleic acid-binding, OB-fold, Ribosomal protein S16 domain, Ribosomal Proteins L2, RNA binding domain, Ribosomal protein L2
Nitab4.5_0000595g0070.1	−56.94444444	3.28E-16	2.84E-12	Unknown
Nitab4.5_0000847g0050.1	−55.55555556	9.57E-09	7.14E-06	Pentatricopeptide repeat
Nitab4.5_0000434g0190.1	−54.54545455	4.04E-06	0.001032214	Unknown
Nitab4.5_0010096g0020.1	−54.25407925	5.44E-13	1.64E-09	Pentatricopeptide repeat, Tetratricopeptide-like helical
Nitab4.5_0000345g0010.1	−54.16666667	3.23E-06	0.000861626	Phosphatidylinositol-glycan biosynthesis class S protein
Nitab4.5_0026095g0010.1	−54.03513996	1.12E-18	1.83E-14	Unknown
Nitab4.5_0010702g0010.1	−53.24041812	5.63E-08	3.09E-05	Pentatricopeptide repeat, Peptidase S8, subtilisin-related, Peptidase S8/S53 domain, Peptidase S8, subtilisin, Ser-active site, Protease-associated domain, PA
Nitab4.5_0002279g0110.1	−53.21151717	1.77E-07	7.97E-05	Unknown
Nitab4.5_0001509g0070.1	−52.05882353	5.49E-06	0.001317357	Pentatricopeptide repeat, Tetratricopeptide-like helical
Nitab4.5_0001392g0070.1	−51.96969697	6.93E-06	0.001591465	Cation-transporting P-type ATPase, HAD-like domain, Divalent cation-transporting P-type ATPase, P-type ATPase, cytoplasmic domain N, P-type ATPase, transmembrane domain, P-type ATPase, A domain, P-type ATPase, phosphorylation site
Nitab4.5_0000159g0040.1	−51.61290323	2.54E-09	2.30E-06	Multi antimicrobial extrusion protein

Nitab4.5_0013415g001 0.1	−50.72340426	2.30E-05	0.004182312	UDP-glucuronosyl/UDP-glucosyltransfer ase
Nitab4.5_0004864g005 0.1	−50.60606061	1.96E-05	0.003685945	Nucleic acid-binding, OB-fold, Ribosomal protein S16 domain, Ribosomal Proteins L2, RNA binding domain, Ribosomal protein L2
Nitab4.5_0005375g001 0.1	−50.55555556	4.39E-05	0.007034698	Pentatricopeptide repeat, Tetratricopeptide-like helical
Nitab4.5_0000170g003 0.1	−50	5.51E-06	0.00132186	CAAX amino terminal protease
Nitab4.5_0010702g001 0.1	−50	4.78E-08	2.70E-05	Pentatricopeptide repeat, Peptidase S8, subtilisin-related, Peptidase S8/S53 domain, Peptidase S8, subtilisin, Ser-active site, Protease-associated domain, PA
Nitab4.5_0004864g005 0.1	−49.68112245	5.42E-06	0.001302954	Nucleic acid-binding, OB-fold, Ribosomal protein S16 domain, Ribosomal Proteins L2, RNA binding domain, Ribosomal protein L2
Nitab4.5_0000692g019 0.1	−48.7804878	1.14E-08	8.25E-06	FBD domain
Nitab4.5_0000020g045 0.1	−48.27586207	7.04E-14	2.95E-10	Pentatricopeptide repeat, Tetratricopeptide-like helical
Nitab4.5_0000894g007 0.1	−47.82608696	1.48E-05	0.002933929	S-adenosylmethionine decarboxylase, conserved site, S-adenosylmethionine decarboxylase, S-adenosylmethionine decarboxylase, core, S-adenosylmethionine decarboxylase subgroup
Nitab4.5_0000159g004 0.1	−47.81783681	2.58E-07	0.000108455	Multi antimicrobial extrusion protein
Nitab4.5_0000163g005 0.1	−46.94444444	5.19E-06	0.001260832	Unknown
Nitab4.5_0000037g014 0.1	−46.34146341	1.82E-09	1.76E-06	EF-Hand 1, calcium-binding site, UDP-glucuronosyl/UDP-glucosyltransfer ase
Nitab4.5_0009514g001 0.1	−45.83333333	3.56E-06	0.000932592	C1-like, DC1, Zinc finger, RING/FYVE/PHD-type
Nitab4.5_0007752g001 0.1	−45.71428571	1.50E-05	0.002961736	Disease resistance protein, NB-ARC, P-loop containing nucleoside triphosphate hydrolase
Nitab4.5_0005637g010 0.1	-44.62405522	4.36E-18	6.65E-14	NADH:ubiquinone oxidoreductase-like, 20kDa subunit, NADH:ubiquinone/plastoquinone oxidoreductase, chain 3
Nitab4.5_0006084g001 0.1	−44.23076923	2.00E-10	2.58E-07	Ribulose bisphosphate carboxylase, large subunit, ferrodoxin-like N-terminal, Ribulose bisphosphate carboxylase, large subunit, C-terminal, Ribulose bisphosphate carboxylase, large subunit, Ribulose bisphosphate carboxylase, large chain, active site, Ribulose bisphosphate carboxylase, large subunit, N-terminal
Nitab4.5_0000195g023 0.1	−43.49061265	2.64E-29	3.63E-24	Armadillo-type fold, Armadillo-like helical
Nitab4.5_0002507g001 0.1	−43.24324324	1.90E-07	8.47E-05	Zinc finger, C6HC-type, Zinc finger, RING-type, Zinc finger, C3HC4

				RING-type, Zinc finger, RING-type, conserved site, Zinc finger, RING/FYVE/PHD-type
Nitab4.5_0001875g0040.1	−42.5	3.16E-05	0.005414388	Glycosyl hydrolase, five-bladed beta-propellor domain, Glycoside hydrolase, family 32, Glycosyl hydrolase family 32, N-terminal, Concanavalin A-like lectin/glucanases superfamily, Glycosyl hydrolase family 32, C-terminal
Nitab4.5_0000929g0130.1	−41.86046512	7.23E-06	0.001648082	Unknown
Nitab4.5_0016571g0010.1	−41.81818182	3.08E-09	2.73E-06	Helicase, superfamily 1/2, ATP-binding domain, DNA/RNA helicase, DEAD/DEAH box type, N-terminal, RNA helicase, DEAD-box type, Q motif, RNA helicase, ATP-dependent, DEAD-box, conserved site, P-loop containing nucleoside triphosphate hydrolase
Nitab4.5_0012427g0010.1	−41.32653061	1.09E-07	5.35E-05	Cullin repeat-like-containing domain, Cullin, N-terminal
Nitab4.5_0006683g0010.1	−40.74074074	2.10E-05	0.003905822	F-box associated interaction domain
Nitab4.5_0002580g0020.1	−40.70972887	1.49E-06	0.000458842	Unknown
Nitab4.5_0003512g0050.1	−40.50179211	3.78E-06	0.000977784	Cytochrome P450
Nitab4.5_0005357g0010.1	−40.42553191	3.59E-06	0.000937882	CrcB homologue
Nitab4.5_0001671g0150.1	−40	6.15E-08	3.32E-05	Unknown
Nitab4.5_0004246g0030.1	−39.04371585	6.54E-06	0.001520501	Alpha 1,4-glycosyltransferase domain, Glycosyltransferase, DXD sugar-binding motif
Nitab4.5_0000429g0020.1	−38.97435897	3.41E-05	0.005758507	Unknown
Nitab4.5_0000010g0110.1	−37.52925237	1.34E-06	0.000423603	P-loop containing nucleoside triphosphate hydrolase
Nitab4.5_0001497g0130.1	−37.21194417	7.14E-10	7.80E-07	Thiolase-like, FAE1/Type III polyketide synthase-like protein, Thiolase-like, subgroup, Very-long-chain 3-ketoacyl-CoA synthase, 3-Oxoacyl-[acyl-carrier-protein (ACP)] synthase III C-terminal
Nitab4.5_0000661g0120.1	−37.14285714	5.03E-06	0.001229191	Unknown
Nitab4.5_0013753g0010.1	−36.95054945	7.82E-06	0.001752357	C1-like, DC1, Zinc finger, RING/FYVE/PHD-type
Nitab4.5_0002418g0160.1	−36.51024455	9.04E-07	0.000303683	Cytochrome c-type biogenesis protein CcmC, Ribosomal protein L23/L15e core domain, Nucleotide-binding, alpha-beta plait, Ribosomal protein L23/L25, conserved site, Ribosomal protein L25/L23, Cytochrome c assembly protein
Nitab4.5_0000201g0120.1	−35.13928493	2.12E-09	1.99E-06	Ribonuclease H domain, Ribonuclease H-like domain
Nitab4.5_0007174g001	−34.77390956	1.04E-07	5.12E-05	Unknown

0.1				
Nitab4.5_0000747g001 0.1	−34.67297084	5.10E-05	0.007901195	Photosystem I PsaA/PsaB
Nitab4.5_0000747g001 0.1	−34.44444444	6.11E-05	0.009148588	Photosystem I PsaA/PsaB
Nitab4.5_0008848g001 0.1	−34.12058905	4.29E-06	0.001086464	Serine/threonine- / dual specificity protein kinase, catalytic domain, Protein kinase domain, Protein kinase-like domain
Nitab4.5_0000027g012 0.1	−33.92857143	4.42E-07	0.000169745	Heat shock protein 70, conserved site, Heat shock protein 70 family
Nitab4.5_0000402g021 0.1	−33.92857143	4.51E-06	0.001129108	Unknown
Nitab4.5_0000135g003 0.1	−33.89830508	2.03E-08	1.32E-05	Zein-binding domain
Nitab4.5_0002418g016 0.1	−33.59044162	9.33E-06	0.002020612	Cytochrome c-type biogenesis protein CcmC, Ribosomal protein L23/L15e core domain, Nucleotide-binding, alpha-beta plait, Ribosomal protein L23/L25, conserved site, Ribosomal protein L25/L23, Cytochrome c assembly protein
Nitab4.5_0002418g016 0.1	−33.36439888	9.35E-06	0.002024921	Cytochrome c-type biogenesis protein CcmC, Ribosomal protein L23/L15e core domain, Nucleotide-binding, alpha-beta plait, Ribosomal protein L23/L25, conserved site, Ribosomal protein L25/L23, Cytochrome c assembly protein
Nitab4.5_0003726g002 0.1	−33.33333333	9.78E-08	4.90E-05	SGNH hydrolase-type esterase domain, Lipase, GDSL
Nitab4.5_0002418g022 0.1	−32.75862069	5.91E-06	0.00140174	Nucleic acid-binding, OB-fold, Ribosomal protein L2, Ribosomal Proteins L2, RNA binding domain
Nitab4.5_0007104g004 0.1	−32.14190876	1.78E-12	4.54E-09	RNA polymerase Rpb2, OB-fold, DNA-directed RNA polymerase, subunit 2, RNA polymerase Rpb2, domain 3, RNA polymerase Rpb2, domain 2, Photosystem antenna protein-like, DNA-directed RNA polymerase, subunit 2, domain 6
Nitab4.5_0000209g010 0.1	−31.98653199	6.52E-05	0.009639164	RNA-dependent RNA polymerase, eukaryotic-type
Nitab4.5_0001003g002 0.1	−31.10275689	7.78E-07	0.00026949	Ribonuclease III domain, Argonaute/Dicer protein, PAZ domain, P-loop containing nucleoside triphosphate hydrolase, Helicase, C-terminal, DNA/RNA helicase, DEAD/DEAH box type, N-terminal, Helicase, superfamily 1/2, ATP-binding domain, Dicer dimerisation domain
Nitab4.5_0000223g018 0.1	−30.88235294	9.63E-06	0.002074111	Unknown
Nitab4.5_0001107g014 0.1	−30.64516129	6.73E-05	0.009873736	Unknown
Nitab4.5_0014277g001 0.1	−30.6122449	5.27E-06	0.001274067	Armadillo, Armadillo-type fold, Armadillo-like helical
Nitab4.5_0001170g007 0.1	−30.28496927	2.44E-05	0.004376231	Uncharacterised protein family Ycf68

Nitab4.5_0001896g0010.1	−30	4.31E-05	0.006920437	NAC domain
Nitab4.5_0001432g0020.1	−29.80769231	2.51E-07	0.00010644	Transcription factor, TCP, Transcription factor TCP subgroup
Nitab4.5_0000194g0090.1	−29.48717949	1.29E-07	6.13E-05	Zinc finger, RING/FYVE/PHD-type, Zinc finger, RING-type
Nitab4.5_0008374g0010.1	−28.98351648	3.72E-06	0.000966931	P-loop containing nucleoside triphosphate hydrolase
Nitab4.5_0005357g0010.1	−28.57142857	3.38E-05	0.005715393	CrcB homologue
Nitab4.5_0005485g0010.1	−28.43406593	3.88E-08	2.26E-05	Mannose-binding lectin, NB-ARC, P-loop containing nucleoside triphosphate hydrolase, Disease resistance protein
Nitab4.5_0004080g0020.1	−28.40020844	6.65E-61	6.39E-55	Unknown
Nitab4.5_0006478g0040.1	−28.2481467	1.10E-12	3.02E-09	Photosystem I PsaA/PsaB
Nitab4.5_0009433g0010.1	−27.73109244	3.02E-11	4.96E-08	NB-ARC, Mannose-binding lectin, Disease resistance protein, P-loop containing nucleoside triphosphate hydrolase
Nitab4.5_0007979g0030.1	−26.88790175	4.30E-05	0.006914899	Photosystem II protein D1, Photosynthetic reaction centre, L/M, Maturase MatK, N-terminal domain, Domain X
Nitab4.5_0006926g0010.1	−26.23376623	1.62E-05	0.003156523	Exocyst complex component Sec10-like
Nitab4.5_0004529g0010.1	−25.80645161	6.45E-05	0.009568278	Hydroxymethylglutaryl-CoA reductase, class I/II, Hydroxymethylglutaryl-CoA reductase, class I/II, substrate-binding, Hydroxymethylglutaryl-CoA reductase, class I/II, conserved site, Hydroxymethylglutaryl-CoA reductase, N-terminal, Hydroxymethylglutaryl-CoA reductase, eukaryotic/arcaheal type, Hydroxymethylglutaryl-CoA reductase, class I/II, catalytic domain, Hydroxymethylglutaryl-CoA reductase, class I/II, NAD/NADP-binding
Nitab4.5_0000220g0160.1	−25.7020757	1.25E-09	1.26E-06	Photosynthetic reaction centre, L/M, Photosystem II PsbD/D2, reaction centre, Photosystem antenna protein-like
Nitab4.5_0001490g0030.1	−25.19936204	4.10E-05	0.006646046	Unknown
**CONTEXT CHG**				
**ID**	**meth.diff**	***p* value**	**q value**	**Note**
Nitab4.5_0003419g0090	−100	2.92641E-17	3.35805E-12	Zinc finger, RING/FYVE/PHD-type, Zinc finger, PHD-type, conserved site, Zinc finger, PHD-type, Zinc finger, PHD-finger, Zinc finger, FYVE/PHD-type
Nitab4.5_0008235g0020	−100	1.74979E-19	5.30653E-14	Protein of unknown function DUF1664
Nitab4.5_0008833g0010	−96.875	8.41519E-16	6.05571E-11	Protein-tyrosine phosphatase, active site, Dual specificity phosphatase, subgroup, catalytic domain, Dual specificity phosphatase, catalytic domain, Dual

				specificity phosphatase, Villin/Gelsolin, Protein-tyrosine/Dual specificity phosphatase
Nitab4.5_0016907g0010	−96.42857143	4.00926E-16	3.18964E-11	Suppressor of forked, Tetratricopeptide-like helical, RNA-processing protein, HAT helix
Nitab4.5_0000025g0620	−96.2962963	1.43126E-14	6.32994E-10	Serine endopeptidase DegP2, Peptidase S1C, Trypsin-like cysteine/serine peptidase domain, Peptidase S1
Nitab4.5_0000745g0010	−96.15384615	1.2401E-13	3.92923E-09	WD40-repeat-containing domain, WD40 repeat, WD40/YVTN repeat-like-containing domain, WD40 repeat, conserved site
Nitab4.5_0000335g0130	−96.15384615	1.26672E-14	5.84582E-10	HAD-like domain, Double-stranded RNA-binding domain, NLI interacting factor
Nitab4.5_0001898g0070	−96	4.98085E-14	1.80747E-09	Trihelix transcription factor GT3, Myb-like domain, C2H2- zinc finger protein family, Homeodomain-like, SANT/Myb domain
Nitab4.5_0002223g0030	−95.23809524	4.01371E-14	1.50807E-09	Helicase/SANT-associated, DNA binding, Homeodomain-like, Myb-like domain, HAS subgroup, SANT/Myb domain
Nitab4.5_0000020g0310	−95	5.75552E-12	9.29142E-08	Winged helix-turn-helix DNA-binding domain, Transcription factor E2F/dimerisation partner (TDP), E2F Family
Nitab4.5_0006574g0100	−95	8.83465E-13	2.01665E-08	WD40 repeat, conserved site, WD40/YVTN repeat-like-containing domain, WD40-repeat-containing domain, WD40 repeat, Small-subunit processome, Utp12, Quinonprotein alcohol dehydrogenase-like superfamily
Nitab4.5_0009504g0030	−95	8.83465E-13	2.01665E-08	Unknown
Nitab4.5_0002348g0020	−95	4.92254E-13	1.26666E-08	Unknown
Nitab4.5_0000377g0140	−94.28571429	2.39853E-16	2.07828E-11	Glycosyl transferase, family 43
Nitab4.5_0000029g0190	−93.75	2.4551E-21	1.15819E-15	PUA-like domain, Pseudouridine synthase/archaeosine transglycosylase, S-adenosylmethionine-dependent methyltransferase
Nitab4.5_0007100g0060	−93.71482176	2.61548E-17	3.161E-12	Pentapeptide repeat
Nitab4.5_0008965g0020	−93.5483871	4.32185E-20	1.52912E-14	Ubiquitin carboxyl-terminal hydrolases family 2, Zinc finger, UBP-type, Zinc finger, RING/FYVE/PHD-type
Nitab4.5_0000010g0010	−93.33333333	5.41209E-16	4.17788E-11	Zinc finger, RanBP2-type
Nitab4.5_0002853g0090	−92.94947121	2.72014E-15	1.53987E-10	Membrane bound O-acyl transferase, MBOAT
Nitab4.5_0002067g0020	−92.85714286	2.75463E-18	4.33164E-13	Nucleotide-binding, alpha-beta plait, RNA recognition motif domain
Nitab4.5_0000617g0060	−92.30769231	7.0736E-18	1.03561E-12	Zinc finger, PHD-finger, Zinc finger, PHD-type, conserved site, Homeobox

				domain, Homeodomain-like, Zinc finger, RING/FYVE/PHD-type, Zinc finger, FYVE/PHD-type, Zinc finger, PHD-type
Nitab4.5_0000006g0150	−92.09401709	6.07896E-19	1.22903E-13	Clathrin, heavy chain/VPS, 7-fold repeat, Vacuolar protein sorting-associated protein 8, central domain, WD40/YVTN repeat-like-containing domain, Zinc finger, RING-type, WD40-repeat-containing domain
Nitab4.5_0003845g0020	−92	8.3585E-13	1.94989E-08	Zinc finger, N-recognin, metazoa, Zinc finger, N-recognin
Nitab4.5_0000984g0080	−92	4.25823E-13	1.10916E-08	Telomere-length maintenance and DNA damage repair
Nitab4.5_0003764g0030	−92	4.25823E-13	1.10916E-08	Unknown
Nitab4.5_0000020g0310	−92	6.31219E-14	2.21487E-09	Winged helix-turn-helix DNA-binding domain, Transcription factor E2F/dimerisation partner (TDP), E2F Family
Nitab4.5_0002575g0020	−92	1.77707E-16	1.67218E-11	Acyl-CoA N-acyltransferase, GNAT domain
Nitab4.5_0003343g0030	−91.4893617	1.20555E-15	7.75523E-11	RNA recognition motif domain, Nucleotide-binding, alpha-beta plait
Nitab4.5_0000347g0030	−91.42857143	7.20991E-14	2.48873E-09	ATP-NAD kinase, PpnK-type, all-beta, Inorganic polyphosphate/ATP-NAD kinase, domain 1, ATP-NAD kinase-like domain, Inorganic polyphosphate/ATP-NAD kinase, predicted
Nitab4.5_0002211g0150	−90.90909091	9.32986E-16	6.5673E-11	Domain of unknown function DUF676, lipase-like
Nitab4.5_0005169g0040	−90.69264069	4.43727E-11	5.26025E-07	Protein kinase domain, Serine/threonine- / dual specificity protein kinase, catalytic domain, Serine/threonine-protein kinase, active site, Protein kinase, ATP binding site, Protein kinase-like domain
Nitab4.5_0002532g0050	−90	1.55572E-11	2.09689E-07	RNA recognition motif domain, Nucleotide-binding, alpha-beta plait
Nitab4.5_0009057g0010	−90	5.3136E-12	8.78809E-08	RNA recognition motif domain, WW domain, Paraneoplastic encephalomyelitis antigen, Nucleotide-binding, alpha-beta plait
Nitab4.5_0009617g0020	−89.40217391	5.1071E-13	1.29841E-08	Cobalamin (vitamin B12)-independent methionine synthase MetE, N-terminal, Cobalamin-independent methionine synthase, Methionine synthase, vitamin-B12 independent
Nitab4.5_0002747g0010	−88.88888889	4.95377E-12	8.37945E-08	Putative S-adenosyl-L-methionine-dependent methyltransferase
Nitab4.5_0001843g0090	−88.88888889	6.50755E-13	1.56098E-08	Heat shock protein 70, conserved site, Heat shock protein 70 family
Nitab4.5_0008661g0030	−88.88888889	3.13358E-14	1.2434E-09	Hydroxymethylglutaryl-CoA reductase, class I/II, catalytic domain, Hydroxymethylglutaryl-CoA reductase,

				class I/II, Hydroxymethylglutaryl-CoA reductase, class I/II, substrate-binding, Hydroxymethylglutaryl-CoA reductase, class I/II, NAD/NADP-binding
Nitab4.5_0010107g0010	−88.46153846	1.25537E-12	2.70557E-08	EF-hand domain pair, EF-Hand 1, calcium-binding site, EF-hand domain, Protein kinase domain, Serine/threonine-protein kinase, active site, Protein kinase, ATP binding site, Protein kinase-like domain, Serine/threonine- / dual specificity protein kinase, catalytic domain
Nitab4.5_0006942g0020	−88.37209302	3.69181E-14	1.41211E-09	Ribosome control protein 1, WD40/YVTN repeat-like-containing domain
Nitab4.5_0001287g0030	−88	1.89619E-15	1.15011E-10	Armadillo-type fold, Coatomer beta subunit (COPB1), Coatomer, beta subunit, C-terminal, Armadillo-like helical, Clathrin/coatomer adaptor, adaptin-like, N-terminal
Nitab4.5_0002944g0010	−87.87878788	6.44495E-13	1.56098E-08	Membrane insertase YidC/Oxa1, C-terminal, Membrane insertase OXA1/ALB3/YidC
Nitab4.5_0016907g0010	−87.73291925	5.44811E-12	8.93098E-08	Suppressor of forked, Tetratricopeptide-like helical, RNA-processing protein, HAT helix
Nitab4.5_0007223g0020	−87.5	4.95377E-12	8.37945E-08	Inhibitor of growth protein, N-terminal, Zinc finger, PHD-type, Zinc finger, FYVE/PHD-type, Zinc finger, PHD-finger, Zinc finger, RING/FYVE/PHD-type
Nitab4.5_0001436g0070	−87.5	1.61358E-16	1.55701E-11	Proteasome, beta-type subunit, conserved site, Proteasome, subunit alpha/beta, Proteasome B-type subunit
Nitab4.5_0005739g0010	−87.17948718	1.03381E-17	1.4631E-12	Pumilio RNA-binding repeat, Armadillo-type fold, Armadillo-like helical
Nitab4.5_0001215g0040	−86.95652174	3.25907E-11	4.04595E-07	Serine-threonine/tyrosine-protein kinase catalytic domain, Serine/threonine- / dual specificity protein kinase, catalytic domain, Protein kinase domain, ACT domain, Protein kinase-like domain, Serine/threonine-protein kinase, active site
Nitab4.5_0000438g0060	−86.9047619	1.91429E-10	1.76303E-06	Endonuclease/exonuclease/phosphatase, AP endonuclease 1, Zinc finger, GRF-type
Nitab4.5_0001701g0190	−85.69604087	2.91226E-12	5.23927E-08	N-acetylglucosaminyltransferase II
Nitab4.5_0001913g0040	−85.23573201	1.41723E-12	2.96031E-08	Unknown
Nitab4.5_0007806g0010	−85.18518519	1.62258E-12	3.26496E-08	Primosome PriB/single-strand DNA-binding, Nucleic acid-binding, OB-fold
Nitab4.5_0001019g0080	−85.18518519	2.04715E-16	1.84929E-11	Diacylglycerol kinase, accessory domain, Diacylglycerol kinase, catalytic domain, ATP-NAD kinase-like domain

Nitab4.5_0005247g003 0	−85	4.46928E-0 9	2.3868E-05	Ubiquitin carboxyl-terminal hydrolases family 2, ICP0-binding domain of Ubiquitin-specific protease 7, MATH, Peptidase C19, ubiquitin carboxyl-terminal hydrolase 2, conserved site, TRAF-like
Nitab4.5_0001315g028 0	−85	8.72218E-1 3	2.01261E-08	Concanavalin A-like lectin/glucanase, subgroup, Protein kinase, ATP binding site, Serine/threonine-protein kinase, active site, Protein kinase-like domain, Protein kinase domain, Serine/threonine- / dual specificity protein kinase, catalytic domain
Nitab4.5_0003065g003 0	−84.86842105	2.8415E-13	7.73352E-09	RNA polymerase Rpb1, domain 1
Nitab4.5_0001904g009 0	−84.61538462	2.29017E-1 1	2.95546E-07	NUDIX hydrolase domain, NUDIX hydrolase domain-like, NUDIX hydrolase, conserved site
Nitab4.5_0000170g022 0	−84.44444444	1.61551E-1 3	4.84098E-09	Protein kinase-like domain, Serine-threonine/tyrosine-protein kinase catalytic domain, Protein kinase domain
Nitab4.5_0000010g001 0	−84.375	1.3081E-13	4.11397E-09	Zinc finger, RanBP2-type
Nitab4.5_0001701g019 0	−84	8.02823E-1 1	8.67322E-07	N-acetylglucosaminyltransferase II
Nitab4.5_0004727g002 0	−84	2.03009E-1 1	2.6685E-07	Ninja
Nitab4.5_0000366g006 0	−84	3.26651E-1 3	8.83359E-09	Uncharacterised protein family UPF0172
Nitab4.5_0005011g004 0	−83.7962963	5.20641E-1 1	5.92707E-07	RNA-dependent RNA polymerase, eukaryotic-type, Nucleotide-binding, alpha-beta plait, RNA recognition motif domain
Nitab4.5_0001364g006 0	−83.66666667	3.07443E-1 2	5.5077E-08	Ribosomal protein L24e domain, Ribosomal protein L24e-related, Ribosomal protein L24e, conserved site, TRASH domain
Nitab4.5_0006677g003 0	−83.37209302	2.09734E-1 1	2.73152E-07	Zinc finger, N-recognin, Zinc finger, N-recognin, metazoa
Nitab4.5_0008661g003 0	−83.33333333	1.57878E-1 3	4.84098E-09	Hydroxymethylglutaryl-CoA reductase, class I/II, catalytic domain, Hydroxymethylglutaryl-CoA reductase, class I/II, Hydroxymethylglutaryl-CoA reductase, class I/II, substrate-binding, Hydroxymethylglutaryl-CoA reductase, class I/II, NAD/NADP-binding
Nitab4.5_0002795g011 0	−83.25062035	3.29565E-1 6	2.69086E-11	Ataxin 2, SM domain, LsmAD domain
Nitab4.5_0005808g003 0	−82.92682927	9.8881E-14	3.20476E-09	Zinc finger, RING/FYVE/PHD-type, Zinc finger, C3HC4 RING-type, Ankyrin repeat-containing domain, Ankyrin repeat, Zinc finger, RING-type, Protein kinase-like domain, Zinc finger, RING-type, conserved site
Nitab4.5_0001878g004 0	−82.92682927	2.41377E-1 4	1.02361E-09	Ribosomal protein S19, superfamily, Ribosomal protein S19/S15, Ribosomal protein S19 conserved site, Ribosomal

				protein S19A/S15e
Nitab4.5_0000029g0190	−82.85714286	6.13252E-18	9.29895E-13	PUA-like domain, Pseudouridine synthase/archaeosine transglycosylase, S-adenosylmethionine-dependent methyltransferase
Nitab4.5_0005058g0010	−82.75862069	1.68216E-12	3.30649E-08	Myb-like domain
Nitab4.5_0000735g0010	−82.60869565	1.43352E-10	1.39275E-06	Small GTPase superfamily, Small GTPase superfamily, Rho type, Small GTPase superfamily, Ras type, Small GTPase superfamily, Rab type, P-loop containing nucleoside triphosphate hydrolase, Small GTP-binding protein domain
Nitab4.5_0007244g0030	−82.60869565	1.61908E-13	4.84098E-09	WD40 repeat, WD40/YVTN repeat-like-containing domain, WD40-repeat-containing domain, RAVE complex protein Rav1 C-terminal
Nitab4.5_0000184g0070	−82.36363636	6.429E-10	4.80703E-06	ERCC4 domain
Nitab4.5_0001296g0080	−82.35599377	5.73507E-17	6.24349E-12	CID domain, RNA polymerase II-binding domain, ENTH/VHS
Nitab4.5_0002013g0020	−82.29166667	4.67668E-11	5.45493E-07	Synaptojanin, N-terminal
Nitab4.5_0001003g0120	−82.14285714	4.12182E-11	4.92963E-07	Nucleotide-sugar transporter, UDP/CMP-sugar transporter
Nitab4.5_0000184g0230	−81.81818182	2.94776E-10	2.4783E-06	Possible tRNA binding domain, Domain of unknown function DUF1726, GNAT domain, Helicase domain
Nitab4.5_0009962g0010	−81.81818182	2.94776E-10	2.4783E-06	Armadillo-type fold
Nitab4.5_0013571g0010	−81.81818182	6.88091E-12	1.08604E-07	NB-ARC, Disease resistance protein, Late blight resistance protein R1, P-loop containing nucleoside triphosphate hydrolase
Nitab4.5_0003077g0040	−81.81818182	4.52797E-12	7.75184E-08	Tetratricopeptide-like helical
Nitab4.5_0003329g0010	−81.81818182	1.48061E-12	3.06647E-08	Mitochodrial transcription termination factor-related
Nitab4.5_0000725g0100	−81.48148148	4.04863E-10	3.23109E-06	UDP-glucuronosyl/UDP-glucosyltransferase
Nitab4.5_0007590g0020	−81.48148148	2.43501E-14	1.02361E-09	Mitochodrial transcription termination factor-related
Nitab4.5_0008661g0030	−81.48148148	1.61863E-14	6.94172E-10	Hydroxymethylglutaryl-CoA reductase, class I/II, catalytic domain, Hydroxymethylglutaryl-CoA reductase, class I/II, Hydroxymethylglutaryl-CoA reductase, class I/II, substrate-binding, Hydroxymethylglutaryl-CoA reductase, class I/II, NAD/NADP-binding
Nitab4.5_0001816g0080	−81.38528139	9.33641E-09	4.36564E-05	Bile acid:sodium symporter
Nitab4.5_0005682g0010	−81.36645963	3.79828E-09	2.08893E-05	Unknown
Nitab4.5_0003911g0060	−81.14919355	1.54984E-12	3.16356E-08	Unknown
Nitab4.5_0001519g019	−81.01851852	3.1838E-10	2.6505E-06	Histone acetyltransferases subunit 3

0				
Nitab4.5_0000839g0100	−80.95238095	3.79305E-10	3.07921E-06	RNA polymerase Rpb7, N-terminal, Ribosomal protein S1, RNA-binding domain, Nucleic acid-binding, OB-fold
Nitab4.5_0012481g0010	−80.95238095	2.34919E-10	2.07793E-06	JmjC domain
Nitab4.5_0002229g0190	−80.95238095	9.37091E-11	9.84813E-07	AP-5 complex subunit zeta-1
Nitab4.5_0007283g0070	−80.95238095	7.77055E-12	1.1997E-07	Dual specificity phosphatase, subgroup, catalytic domain, Dual specificity phosphatase, Dual specificity phosphatase, catalytic domain, Protein-tyrosine phosphatase, active site, Protein-tyrosine/Dual specificity phosphatase
Nitab4.5_0010710g0030	−80.95238095	2.53637E-12	4.78612E-08	Origin recognition complex, subunit 3
Nitab4.5_0001155g0010	−80.76923077	2.1411E-10	1.93005E-06	Leucine-rich repeat, CAP Gly-rich domain
Nitab4.5_0000294g0080	−80.64516129	2.54442E-14	1.05011E-09	WD40/YVTN repeat-like-containing domain, WD40 repeat, SGT1, WD40-repeat-containing domain, WD40 repeat, conserved site, G-protein beta WD-40 repeat
Nitab4.5_0008661g0030	−80.3030303	1.93722E-10	1.78029E-06	Hydroxymethylglutaryl-CoA reductase, class I/II, catalytic domain, Hydroxymethylglutaryl-CoA reductase, class I/II, Hydroxymethylglutaryl-CoA reductase, class I/II, substrate-binding, Hydroxymethylglutaryl-CoA reductase, class I/II, NAD/NADP-binding
Nitab4.5_0006277g0010	−80.2962963	2.16257E-10	1.94528E-06	CID domain, PWWP domain
Nitab4.5_0006028g0020	−80	1.3046E-09	8.6818E-06	Sec7 domain, Sec7 domain, alpha orthogonal bundle, Domain of unknown function DUF1981, Sec7 associated, Armadillo-like helical, Armadillo-type fold
Nitab4.5_0001052g0090	−80	8.39423E-10	5.95538E-06	Domain of unknown function DUF4094, Glycosyl transferase, family 31
Nitab4.5_0005901g0020	−80	7.20408E-10	5.25544E-06	Helicase, C-terminal, P-loop containing nucleoside triphosphate hydrolase, Helicase, superfamily 1/2, ATP-binding domain, RNA helicase, ATP-dependent, DEAD-box, conserved site, RNA helicase, DEAD-box type, Q motif, DNA/RNA helicase, DEAD/DEAH box type, N-terminal
Nitab4.5_0006523g0030	−80	1.96586E-10	1.79882E-06	Cyclic nucleotide-binding-like, Cyclic nucleotide-binding domain, Potassium channel, voltage-dependent, EAG/ELK/ERG, IQ motif, EF-hand binding site, Ion transport domain, RmlC-like jelly roll fold
Nitab4.5_0001587g0080	−80	3.90167E-13	1.03534E-08	Armadillo-like helical, Armadillo, Armadillo-type fold
Nitab4.5_0004028g005	−79.7979798	2.54565E-0	1.49905E-05	Serine/threonine-protein kinase

0		9		CTR1/EDR1, Serine-threonine/tyrosine-protein kinase catalytic domain, Protein kinase domain, Serine/threonine-protein kinase, active site, Protein kinase-like domain, Peptidyl-prolyl cis-trans isomerase, FKBP-type, domain, Protein kinase, ATP binding site, Serine/threonine- / dual specificity protein kinase, catalytic domain
Nitab4.5_0002510g0050	−79.66666667	2.19363E-09	1.32108E-05	Armadillo-like helical, Armadillo-type fold
Nitab4.5_0004458g0020	−79.62962963	4.40783E-10	3.46708E-06	Cation efflux protein, Cation efflux protein transmembrane domain
Nitab4.5_0000446g0250	−79.54911433	1.50922E-09	9.72344E-06	Ribosomal protein S3, C-terminal
Nitab4.5_0008125g0020	−79.31034483	1.82954E-10	1.70346E-06	Protein kinase-like domain, Serine/threonine- / dual specificity protein kinase, catalytic domain, Protein kinase domain, Serine/threonine-protein kinase, active site
Nitab4.5_0000535g0110	−79.31034483	1.78132E-11	2.3783E-07	Protein phosphatase 2C (PP2C)-like domain, Protein phosphatase 2C
Nitab4.5_0005126g0020	−79.16666667	2.70063E-10	2.32109E-06	Putative 5-3 exonuclease, 5'-3' exoribonuclease
Nitab4.5_0003943g0020	−79.16666667	6.10392E-11	6.7842E-07	Oxysterol-binding protein, conserved site, Oxysterol-binding protein
Nitab4.5_0000095g0200	−79.16666667	9.93587E-12	1.45466E-07	Nucleic acid-binding, OB-fold, P-loop containing nucleoside triphosphate hydrolase, Helicase, C-terminal, Ribosomal protein S1, RNA-binding domain, DNA/RNA helicase, ATP-dependent, DEAH-box type, conserved site, Helicase-associated domain, DNA/RNA helicase, DEAD/DEAH box type, N-terminal, Helicase, superfamily 1/2, ATP-binding domain, Domain of unknown function DUF1605, RNA-binding domain, S1
Nitab4.5_0013404g0010	−78.97435897	6.17495E-12	9.8561E-08	Frigida-like
Nitab4.5_0000687g0090	−78.79417879	1.61497E-13	4.84098E-09	Phosphoesterase domain, Serine/threonine-specific protein phosphatase/bis(5-nucleosyl)-tetraphosphatase
Nitab4.5_0005411g0010	−78.62318841	7.56313E-09	3.66984E-05	Unknown
Nitab4.5_0000261g0010	−78.40112202	1.10427E-09	7.50455E-06	Selenoprotein, Rdx type, Thioredoxin-like fold, Selenoprotein T
Nitab4.5_0000352g0080	−78.37837838	5.09411E-11	5.86132E-07	V-type ATPase, V0 complex, subunit 116kDa, ATPase, V0 complex, subunit 116kDa, eukaryotic
Nitab4.5_0008893g0010	−78.32080201	5.80787E-11	6.54077E-07	Unknown
Nitab4.5_0000956g0200	−78.26086957	1.53761E-09	9.86144E-06	Nucleolar 27S pre-rRNA processing, Urb2/Npa2, C-terminal

Nitab4.5_0009312g0010	−78.26086957	3.40887E-10	2.82086E-06	HAD-like domain, Trehalose-phosphatase, Glycosyl transferase, family 20
Nitab4.5_0001296g0080	−78.22128852	9.50356E-14	3.12788E-09	CID domain, RNA polymerase II-binding domain, ENTH/VHS
Nitab4.5_0000358g0060	−78.04878049	1.27846E-16	1.27602E-11	Unknown
Nitab4.5_0001030g0110	−77.77777778	3.40893E-10	2.82086E-06	Histone deacetylase interacting, Paired amphipathic helix
Nitab4.5_0000617g0060	−77.77777778	4.5463E-14	1.67847E-09	Zinc finger, PHD-finger, Zinc finger, PHD-type, conserved site, Homeobox domain, Homeodomain-like, Zinc finger, RING/FYVE/PHD-type, Zinc finger, FYVE/PHD-type, Zinc finger, PHD-type
Nitab4.5_0009403g0010	−77.27272727	2.62459E-11	3.32637E-07	Tetratricopeptide-like helical, Pentatricopeptide repeat
Nitab4.5_0001286g0070	−76.96969697	9.35896E-11	9.84813E-07	DNA glycosylase, HhH-GPD domain, Helix-turn-helix, base-excision DNA repair, C-terminal
Nitab4.5_0001264g0010	−76.95238095	1.05856E-08	4.81195E-05	6-phosphogluconate dehydrogenase, C-terminal-like, Aldolase-type TIM barrel, Dehydrogenase, multihelical, Ketose-bisphosphate aldolase, class-II, 6-phosphogluconate dehydrogenase, NADP-binding, NAD(P)-binding domain, Hydroxy monocarboxylic acid anion dehydrogenase, HIBADH-type, Protein of unknown function, DUF1537
Nitab4.5_0000212g0130	−76.83982684	7.80762E-08	0.000249066	Serine/threonine- / dual specificity protein kinase, catalytic domain, Protein kinase-like domain, Kinase associated domain 1 (KA1), Ubiquitin-associated domain/translation elongation factor EF-Ts, N-terminal, Protein kinase, ATP binding site, Protein kinase domain, Serine/threonine-protein kinase, active site, Ubiquitin-associated/translation elongation factor EF1B, N-terminal, eukaryote, KA1 domain/Ssp2 C-terminal domain
Nitab4.5_0003294g0010	−76.66666667	3.53277E-10	2.90683E-06	Ankyrin repeat, IQ motif, EF-hand binding site, Ankyrin repeat-containing domain, Immunoglobulin E-set, CG-1 DNA-binding domain
Nitab4.5_0001271g0100	−76.38888889	6.05908E-10	4.57745E-06	Signal recognition particle, SRP54 subunit, Signal recognition particle, SRP54 subunit, helical bundle, Signal recognition particle, SRP54 subunit, GTPase domain, AAA+ ATPase domain, Signal recognition particle, SRP54 subunit, M-domain, P-loop containing nucleoside triphosphate hydrolase, Signal recognition particle, SRP54 subunit, eukaryotic
Nitab4.5_0002906g014	−76.31578947	3.00373E-1	1.67803E-10	Protein kinase-like domain, Protein

0		5		kinase domain, Serine/threonine-protein kinase, active site, Serine/threonine- / dual specificity protein kinase, catalytic domain
Nitab4.5_0012199g0010	−76.19047619	1.4199E-07	0.000401317	Heat shock transcription factor family, Heat shock transcription factor, plant, Heat shock factor (HSF)-type, DNA-binding, Winged helix-turn-helix DNA-binding domain
Nitab4.5_0005936g0010	−76.19047619	3.62596E-09	2.01768E-05	Calmodulin binding protein-like
Nitab4.5_0005682g0010	−76.19047619	1.61729E-09	1.02693E-05	Unknown
Nitab4.5_0001019g0080	−76.19047619	2.6578E-12	4.9402E-08	Diacylglycerol kinase, accessory domain, Diacylglycerol kinase, catalytic domain, ATP-NAD kinase-like domain
Nitab4.5_0000202g0100	−76.06837607	2.59653E-12	4.87797E-08	Zinc finger, RING/FYVE/PHD-type
Nitab4.5_0000628g0090	−76	1.96169E-11	2.58659E-07	NADPH-dependent FMN reductase-like, Flavoprotein WrbA, Flavodoxin/nitric oxide synthase
Nitab4.5_0001203g0040	−76	8.7221E-12	1.30854E-07	K Homology domain, K Homology domain, type 1
Nitab4.5_0001437g0020	−75.98814229	1.02799E-11	1.49294E-07	4'-phosphopantetheinyl transferase superfamily
Nitab4.5_0000135g0040	−75.45219638	2.50148E-12	4.74135E-08	Armadillo-like helical, STAG, Armadillo-type fold, Stromalin conservative domain, Domain of unknown function DUF4283
Nitab4.5_0000185g0360	−75.16666667	7.87714E-09	3.79617E-05	Protein of unknown function DUF408
Nitab4.5_0002882g0030	−75.11961722	2.47888E-09	1.46379E-05	EMSY N-terminal, Agenet-like domain, Tudor-like, plant
Nitab4.5_0002535g0010	−75	1.35268E-08	5.89039E-05	WD40 repeat, WD40 repeat, conserved site, Small-subunit processome, Utp12, WD40/YVTN repeat-like-containing domain, G-protein beta WD-40 repeat, WD40-repeat-containing domain, Quinonprotein alcohol dehydrogenase-like superfamily
Nitab4.5_0002097g0080	−75	8.64273E-09	4.09523E-05	Peptidase M16, C-terminal domain, Metalloenzyme, LuxS/M16 peptidase-like, Peptidase M16 domain, Peptidase M16C associated
Nitab4.5_0010206g0030	−75	4.72528E-09	2.49531E-05	Unknown
Nitab4.5_0002223g0030	−75	2.43173E-09	1.44399E-05	Helicase/SANT-associated, DNA binding, Homeodomain-like, Myb-like domain, HAS subgroup, SANT/Myb domain
Nitab4.5_0002973g0040	−75	2.43173E-09	1.44399E-05	Golgi SNAP receptor complex, subunit 1, Delta endotoxin, N-terminal
Nitab4.5_0003246g0010	−75	8.67751E-10	6.08966E-06	Reticulon, Nucleotide-diphospho-sugar transferase
Nitab4.5_0002073g0060	−75	1.11861E-10	1.14922E-06	Allergen V5/Tpx-1-related, conserved site, CAP domain, Cysteine-rich secretory protein, allergen

				V5/Tpx-1-related
Nitab4.5_0000556g0250	−74.63768116	4.92249E-08	0.000169227	DAD/Ost2
Nitab4.5_0002967g0040	−74.5	4.9737E-10	3.86052E-06	Poly A polymerase, head domain
Nitab4.5_0005478g0040	−74.41471572	9.82454E-09	4.54384E-05	Pre-mRNA-splicing factor Cwf15/Cwc15
Nitab4.5_0001913g0040	−74.31761787	1.45446E-09	9.42826E-06	Unknown
Nitab4.5_0007165g0020	−74.27055703	5.03524E-09	2.62956E-05	CCAAT-binding factor, conserved site, CCAAT-binding transcription factor, subunit B
Nitab4.5_0007062g0050	−74.26086957	1.57277E-08	6.64433E-05	Helicase, C-terminal, P-loop containing nucleoside triphosphate hydrolase
Nitab4.5_0000775g0100	−74.0942029	2.54999E-08	9.92357E-05	Serine/threonine-specific protein phosphatase/bis(5-nucleosyl)-tetraphosphatase, Phosphoesterase domain
Nitab4.5_0006523g0030	−74.07407407	2.73667E-09	1.58949E-05	Cyclic nucleotide-binding-like, Cyclic nucleotide-binding domain, Potassium channel, voltage-dependent, EAG/ELK/ERG, IQ motif, EF-hand binding site, Ion transport domain, RmlC-like jelly roll fold
Nitab4.5_0005196g0010	−73.94957983	1.40814E-08	6.09438E-05	Protein of unknown function DUF4336
Nitab4.5_0000051g0330	−73.91304348	8.04356E-09	3.86191E-05	Cytoplasmic FMR1-interacting, Cytoplasmic FMR1-interacting, subgroup
Nitab4.5_0000524g0040	−73.88663968	6.21771E-10	4.68063E-06	Unknown
Nitab4.5_0002050g0030	−73.77777778	5.95201E-09	3.00484E-05	Probable transposase, Ptta/En/Spm, plant
Nitab4.5_0001398g0020	−73.61111111	8.32773E-10	5.94242E-06	Unknown
Nitab4.5_0000202g0100	−73.29472329	2.77477E-13	7.62633E-09	Zinc finger, RING/FYVE/PHD-type
Nitab4.5_0000991g0110	−73.27272727	3.20728E-08	0.00011966	Myc-type, basic helix-loop-helix (bHLH) domain
Nitab4.5_0001398g0020	−73.22222222	1.97482E-10	1.80313E-06	Unknown
Nitab4.5_0000059g0520	−73.07692308	5.09221E-09	2.63983E-05	F-box domain, Galactose oxidase/kelch, beta-propeller, F-box associated interaction domain, F-box associated domain, type 1
Nitab4.5_0000691g0250	−73.07692308	5.09221E-09	2.63983E-05	Ubiquinol cytochrome reductase, transmembrane domain, Rieske [2Fe-2S] iron-sulphur domain, Rieske iron-sulphur protein, Rieske iron-sulphur protein, C-terminal, Ubiquinol-cytochrome c reductase, iron-sulphur subunit
Nitab4.5_0003943g0020	−73.07692308	4.5064E-10	3.53007E-06	Oxysterol-binding protein, conserved site, Oxysterol-binding protein
Nitab4.5_0004414g0010	−73.06763285	6.80569E-09	3.34822E-05	DNA ligase, ATP-dependent, C-terminal, DNA ligase, ATP-dependent, N-terminal, DNA ligase, ATP-dependent, central, DNA

				ligase, ATP-dependent, Nucleic acid-binding, OB-fold, DNA ligase, ATP-dependent, conserved site
Nitab4.5_0003229g0030	−72.95238095	1.22428E-07	0.000356514	CRAL-TRIO domain, CRAL/TRIO, N-terminal domain
Nitab4.5_0000410g0030	−72.9312763	1.5169E-08	6.45975E-05	Histone deacetylase superfamily, Zinc finger, RanBP2-type, Histone deacetylase domain
Nitab4.5_0000185g0290	−72.72727273	2.60263E-08	0.000100547	Transposase, MuDR, plant
Nitab4.5_0000378g0080	−72.61904762	1.96071E-07	0.000523564	Zinc finger, LSD1-type
Nitab4.5_0002788g0030	−72.4137931	1.15944E-11	1.64089E-07	Cysteine alpha-hairpin motif superfamily
Nitab4.5_0000168g0060	−72.22222222	2.79195E-11	3.51748E-07	GrpE nucleotide exchange factor, GrpE nucleotide exchange factor, head, GrpE nucleotide exchange factor, coiled-coil
Nitab4.5_0008235g0020	−72.02380952	1.13606E-09	7.66838E-06	Protein of unknown function DUF1664
Nitab4.5_0004147g0010	−72	2.48118E-07	0.000628546	Phospholipase-like
Nitab4.5_0000288g0080	−72	2.15463E-08	8.65467E-05	Unknown
Nitab4.5_0003848g0040	−72	2.67621E-10	2.30945E-06	ATPase, AAA-type, core, AAA+ ATPase domain, ATPase, AAA-type, conserved site, Vps4 oligomerisation, C-terminal, P-loop containing nucleoside triphosphate hydrolase
Nitab4.5_0003065g0030	−72	9.63674E-12	1.42066E-07	RNA polymerase Rpb1, domain 1
Nitab4.5_0000156g0190	−71.86147186	5.23939E-07	0.001134375	B30.2/SPRY domain, Ubiquitin conjugation factor E4, core, Concanavalin A-like lectin/glucanases superfamily, SPla/RYanodine receptor subgroup, SPla/RYanodine receptor SPRY
Nitab4.5_0004210g0020	−71.66666667	3.88615E-07	0.000908067	PWWP domain
Nitab4.5_0009964g0020	−71.63029525	7.90186E-12	1.21555E-07	Ubiquitin carboxyl-terminal hydrolases family 2, Peptidase C19, ubiquitin carboxyl-terminal hydrolase 2, conserved site, TRAF-like, MATH, ICP0-binding domain of Ubiquitin-specific protease 7
Nitab4.5_0000063g0280	−71.42857143	1.04116E-08	4.75322E-05	Protein notum homologue
Nitab4.5_0005230g0070	−71.42857143	6.30585E-09	3.14976E-05	Pentatricopeptide repeat, Tetratricopeptide-like helical
Nitab4.5_0008768g0060	−71.3900135	4.01093E-11	4.83454E-07	Unknown
Nitab4.5_0000667g0050	−71.28205128	2.03459E-08	8.25846E-05	Longin-like domain, Coatomer delta subunit, Clathrin adaptor, mu subunit, C-terminal
Nitab4.5_0000277g0020	−71.09181141	1.24011E-08	5.49029E-05	DnaJ domain, Protein of unknown function DUF3752
Nitab4.5_0000775g0150	−70.96774194	1.26401E-08	5.57864E-05	Zinc finger, RING-type, Zinc finger, RING-type, conserved site, Zinc finger,

				RING/FYVE/PHD-type
Nitab4.5_0000029g0220	−70.83333333	1.16873E-08	5.20968E-05	Cyclin-like, Cyclin, N-terminal, Cyclin, C-terminal domain
Nitab4.5_0003013g0030	−70.83333333	1.16873E-08	5.20968E-05	WD40/YVTN repeat-like-containing domain, Quinonprotein alcohol dehydrogenase-like superfamily
Nitab4.5_0000998g0040	−70.83333333	4.75438E-09	2.50445E-05	Pentatricopeptide repeat, Tetratricopeptide-like helical
Nitab4.5_0003419g0090	−70.83333333	4.75438E-09	2.50445E-05	Zinc finger, RING/FYVE/PHD-type, Zinc finger, PHD-type, conserved site, Zinc finger, PHD-type, Zinc finger, PHD-finger, Zinc finger, FYVE/PHD-type
Nitab4.5_0009806g0010	−70.83333333	3.08577E-09	1.75387E-05	Tetratricopeptide repeat, Tetratricopeptide-like helical
Nitab4.5_0000269g0100	−70.83333333	2.02461E-09	1.23505E-05	ABC transporter-like, P-loop containing nucleoside triphosphate hydrolase, AAA+ ATPase domain, ABC transporter A, ABCA, ABC transporter, conserved site
Nitab4.5_0000687g0090	−70.58823529	1.21383E-12	2.67025E-08	Phosphoesterase domain, Serine/threonine-specific protein phosphatase/bis(5-nucleosyl)-tetraphosphatase
Nitab4.5_0002112g0090	−70.47619048	1.61327E-07	0.000445352	CinA, C-terminal, Rossmann-like alpha/beta/alpha sandwich fold, Cytidyltransferase-like domain
Nitab4.5_0000036g0110	−70.37037037	1.28256E-10	1.28127E-06	Transmembrane protein 194
Nitab4.5_0006512g0050	−70.28985507	9.57406E-08	0.000294131	BING4, C-terminal domain, WD40-repeat-containing domain, WD40/YVTN repeat-like-containing domain, WD40 repeat
Nitab4.5_0002763g0040	−70.0265252	2.11578E-08	8.53091E-05	BTB/POZ fold, SKP1 component, SKP1 component, POZ domain, E3 ubiquitin ligase, SCF complex, Skp subunit, SKP1 component, dimerisation
Nitab4.5_0010518g0020	−70	4.71181E-08	0.000163976	Threonine dehydratase, biosynthetic, Tryptophan synthase beta subunit-like PLP-dependent enzymes superfamily, Serine/threonine dehydratase, pyridoxal-phosphate-binding site, Threonine dehydratase, C-terminal regulatory domain
Nitab4.5_0003911g0060	−70	7.60537E-11	8.32227E-07	Unknown
Nitab4.5_0008661g0030	−69.85111663	3.75714E-09	2.07436E-05	Hydroxymethylglutaryl-CoA reductase, class I/II, catalytic domain, Hydroxymethylglutaryl-CoA reductase, class I/II, Hydroxymethylglutaryl-CoA reductase, class I/II, substrate-binding, Hydroxymethylglutaryl-CoA reductase, class I/II, NAD/NADP-binding
Nitab4.5_0000826g0140	−69.79166667	3.2283E-08	0.000120233	WD40-repeat-containing domain, WD40 repeat, WD40 repeat, conserved site, WD40/YVTN repeat-like-containing domain, Pre-mRNA processing factor 4

				(PRP4)-like, U4/U6 small nuclear ribonucleoprotein Prp4, Splicing factor motif, G-protein beta WD-40 repeat
Nitab4.5_0000341g0060	−69.6969697	2.75173E-09	1.59605E-05	Protein kinase-like domain, UbiB domain
Nitab4.5_0000775g0100	−69.65811966	8.41741E-08	0.000265514	Serine/threonine-specific protein phosphatase/bis(5-nucleosyl)-tetraphosphatase, Phosphoesterase domain
Nitab4.5_0002182g0100	−69.60600375	1.42664E-09	9.33304E-06	Glycosyl transferase, family 14
Nitab4.5_0002592g0020	−69.56521739	1.52855E-08	6.49631E-05	BTB/POZ fold, MATH, TRAF-like, BTB/POZ-like, BTB/POZ
Nitab4.5_0005169g0050	−69.56521739	6.86426E-10	5.05679E-06	K Homology domain
Nitab4.5_0000519g0080	−69.56521739	1.77831E-10	1.66672E-06	Membrane-anchored ubiquitin-fold protein, HCG-1, Ubiquitin supergroup
Nitab4.5_0008996g0010	−69.23076923	1.36921E-10	1.34477E-06	Mediator complex, subunit Med14
Nitab4.5_0000175g0190	−69.04761905	1.24318E-06	0.002228036	Tetratricopeptide-like helical, Pentatricopeptide repeat
Nitab4.5_0000868g0040	−68.97233202	1.19714E-06	0.002164708	Tetratricopeptide repeat, Tetratricopeptide repeat-containing domain, Tetratricopeptide TPR1, Tetratricopeptide-like helical
Nitab4.5_0000104g0330	−68.96551724	2.8781E-09	1.6513E-05	Unknown
Nitab4.5_0000335g0080	−68.77470356	1.0708E-07	0.000320166	RNA-binding, CRM domain
Nitab4.5_0002021g0030	−68.75	6.4309E-10	4.80703E-06	Unknown
Nitab4.5_0000014g0310	−68.75	4.34545E-11	5.16797E-07	Pentatricopeptide repeat, Tetratricopeptide-like helical
Nitab4.5_0010244g0020	−68.58974359	2.33448E-07	0.000599812	NAD(P)-binding domain, Glucose/ribitol dehydrogenase
Nitab4.5_0000519g0320	−68.57142857	1.02301E-08	4.69562E-05	Zinc finger, RING/FYVE/PHD-type, Zinc finger, RING-type, Zinc finger, RING-type, conserved site
Nitab4.5_0007244g0030	−68.55172414	1.79935E-08	7.43873E-05	WD40 repeat, WD40/YVTN repeat-like-containing domain, WD40-repeat-containing domain, RAVE complex protein Rav1 C-terminal
Nitab4.5_0006523g0030	−68.22742475	3.91352E-07	0.000912455	Cyclic nucleotide-binding-like, Cyclic nucleotide-binding domain, Potassium channel, voltage-dependent, EAG/ELK/ERG, IQ motif, EF-hand binding site, Ion transport domain, RmlC-like jelly roll fold
Nitab4.5_0000052g0050	−68.18181818	2.08144E-06	0.00332227	AP-1, 2,4 complex subunit beta, Beta-adaptin appendage, C-terminal subdomain, Armadillo-like helical, Armadillo-type fold, Beta2-adaptin/TBP, C-terminal domain, AP complex subunit beta, Coatomer/calthrin adaptor appendage, C-terminal subdomain, Clathrin/coatomer adaptor, adaptin-like, N-terminal
Nitab4.5_0008923g001	−68.18181818	1.46236E-0	6.25887E-05	Leucine-rich repeat, Leucine-rich repeat,

0		8		typical subtype, Leucine rich repeat 4, Leucine-rich repeat-containing N-terminal, type 2
Nitab4.5_0002043g0080	−68.09302326	3.81261E-09	2.09409E-05	Partial AB-hydrolase lipase domain
Nitab4.5_0000366g0060	−68	3.02942E-09	1.72878E-05	Uncharacterised protein family UPF0172
Nitab4.5_0001622g0040	−67.98029557	7.78065E-08	0.000249066	UBA-like, Ubiquitin-associated domain/translation elongation factor EF-Ts, N-terminal, Aspartic peptidase, DDI1-type, Ubiquitin-associated/translation elongation factor EF1B, N-terminal, eukaryote, Aspartic peptidase domain
Nitab4.5_0002167g0040	−67.85714286	3.23061E-12	5.76317E-08	Cytochrome b-c1 complex subunit 8, plants, Cytochrome b-c1 complex subunit 8
Nitab4.5_0005161g0050	−67.85714286	1.86978E-13	5.43739E-09	Nuclear transport factor 2, eukaryote, Nuclear transport factor 2
Nitab4.5_0000496g0030	−67.85714286	8.4897E-07	0.001645894	WSTF/Acf1/Cbp146, DDT domain superfamily, DDT domain, DDT domain, subgroup
Nitab4.5_0000687g0130	−67.78378378	4.03614E-08	0.000144978	ICP0-binding domain of Ubiquitin-specific protease 7
Nitab4.5_0008454g0020	−67.65734266	6.89895E-09	3.38576E-05	Glycoside hydrolase, superfamily, Glycoside hydrolase, family 35, Beta-galactosidase 1-like, Glycoside hydrolase, catalytic domain, Galactose-binding domain-like, Glycoside hydrolase, family 35, conserved site
Nitab4.5_0005739g0010	−67.56756757	2.762E-12	5.03678E-08	Pumilio RNA-binding repeat, Armadillo-type fold, Armadillo-like helical
Nitab4.5_0000301g0070	−67.5	6.91187E-07	0.001408833	G-box binding, MFMR, Basic-leucine zipper domain
Nitab4.5_0003548g0040	−67.49482402	1.71421E-06	0.002863133	Oligopeptide transporter, OPT superfamily
Nitab4.5_0004656g0030	−67.44186047	1.02841E-10	1.07018E-06	PC-Esterase, PMR5 N-terminal domain
Nitab4.5_0001036g0110	−67.43589744	1.23133E-07	0.000357804	Cytochrome P450, conserved site, Cytochrome P450, Cytochrome P450, E-class, group II
Nitab4.5_0002064g0050	−67.37179487	5.4755E-11	6.21592E-07	Ribonucleoprotein LSM domain, Like-Sm (LSM) domain
Nitab4.5_0008962g0040	−67.34299517	5.1502E-10	3.97571E-06	Domain of unknown function DUF1995
Nitab4.5_0003194g0030	−67.27272727	3.06908E-07	0.00075234	Cytochrome b-c1 complex subunit 8, Cytochrome b-c1 complex subunit 8, plants
Nitab4.5_0007033g0060	−67.22408027	8.83724E-07	0.001696397	Nuclear cap-binding protein subunit 2, RNA recognition motif domain, Nucleotide-binding, alpha-beta plait
Nitab4.5_0002068g0060	−67.01298701	1.55621E-09	9.95072E-06	TATA-box binding protein, Beta2-adaptin/TBP, C-terminal domain
Nitab4.5_0005682g0010	−66.77115987	5.54039E-07	0.001181809	Unknown

Nitab4.5_0001296g0070	−66.70538134	4.17445E-15	2.2435E-10	5-AMP-activated protein kinase, beta subunit, interaction domain, Immunoglobulin E-set
Nitab4.5_0000834g0010	−66.66666667	2.33526E-07	0.000599812	Protein of unknown function DUF1664
Nitab4.5_0002029g0060	−66.66666667	1.0338E-07	0.000310983	Nicotinate phosphoribosyltransferase family, Quinolinate phosphoribosyl transferase, C-terminal, Nicotinate phosphoribosyltransferase pncB type
Nitab4.5_0005011g0040	−66.66666667	1.43877E-08	6.18284E-05	RNA-dependent RNA polymerase, eukaryotic-type, Nucleotide-binding, alpha-beta plait, RNA recognition motif domain
Nitab4.5_0003902g0080	−66.66666667	7.95303E-10	5.71345E-06	Mammalian cell entry-related
Nitab4.5_0001524g0060	−66.48550725	4.76697E-07	0.00105965	Serine/threonine-protein kinase, active site, Protein kinase, ATP binding site, Mitogen-activated protein (MAP) kinase, conserved site, Protein kinase-like domain, Protein kinase domain, Serine/threonine- / dual specificity protein kinase, catalytic domain
Nitab4.5_0000202g0100	−66.28571429	6.98448E-08	0.000227759	Zinc finger, RING/FYVE/PHD-type
Nitab4.5_0001025g0040	−66.14285714	5.18191E-07	0.001123075	AAA+ ATPase domain, ABC transporter type 1, transmembrane domain, ABC transporter, conserved site, ABC transporter-like, P-loop containing nucleoside triphosphate hydrolase, ABC transporter, transmembrane domain
Nitab4.5_0002114g0160	−65.90909091	1.26014E-07	0.000363635	Unknown
Nitab4.5_0000029g0250	−65.90909091	4.58188E-08	0.000161039	Lytic transglycosylase-like SLT domain, Lysozyme-like domain
Nitab4.5_0004656g0030	−65.85081585	2.43365E-07	0.000618721	PC-Esterase, PMR5 N-terminal domain
Nitab4.5_0000142g0470	−65.82491582	4.87016E-07	0.001074152	DNA/RNA helicase, DEAD/DEAH box type, N-terminal, Helicase, superfamily 1/2, ATP-binding domain, Helicase, C-terminal, Zinc finger, CCHC-type, RNA helicase, DEAD-box type, Q motif, P-loop containing nucleoside triphosphate hydrolase
Nitab4.5_0000895g0120	−65.76923077	3.89164E-06	0.00532996	Sugar transporter, conserved site, Major facilitator superfamily domain, Major facilitator superfamily domain, general substrate transporter, Sugar/inositol transporter, General substrate transporter
Nitab4.5_0001319g0010	−65.625	5.98027E-09	3.01194E-05	Pyridoxal phosphate-dependent transferase, major region, subdomain 2, EGF-like, alliinase, Pyridoxal phosphate-dependent transferase, major region, subdomain 1, Pyridoxal phosphate-dependent transferase, Allinase, C-terminal

Nitab4.5_0003294g0020	−65.54621849	1.43083E-07	0.000403115	Small GTPase superfamily, ARF type, Small GTPase superfamily, SAR1-type, Small GTPase superfamily, ARF/SAR type, P-loop containing nucleoside triphosphate hydrolase, Small GTP-binding protein domain
Nitab4.5_0000159g0030	−65.51724138	1.31725E-09	8.75227E-06	Phosphoesterase domain
Nitab4.5_0003436g0140	−65.38461538	7.79192E-08	0.000249066	Glycosyl transferase, family 48, Callose synthase, 1,3-beta-glucan synthase subunit FKS1-like, domain-1
Nitab4.5_0004665g0070	−65.38461538	4.99091E-08	0.000170551	Target SNARE coiled-coil domain, Sec20
Nitab4.5_0001339g0050	−65.38461538	3.23395E-08	0.000120337	Armadillo-type fold, Zinc finger, N-recognin, metazoa, Zinc finger, N-recognin, E3 ubiquitin ligase, UBR4
Nitab4.5_0001749g0020	−65.27777778	2.80457E-09	1.61567E-05	Isopenicillin N synthase-like
Nitab4.5_0000705g0060	−65.2173913	1.51815E-07	0.000424587	Tetratricopeptide repeat, Tetratricopeptide-like helical
Nitab4.5_0002085g0040	−65.2173913	2.30536E-08	9.14764E-05	DNA/RNA-binding protein Alba-like
Nitab4.5_0001642g0040	−65	7.24583E-07	0.001457315	DNA glycosylase, Helix-turn-helix, base-excision DNA repair, C-terminal, HhH-GPD domain
Nitab4.5_0005425g0040	−65	2.25075E-07	0.00058269	Protein kinase-like domain, UbiB domain
Nitab4.5_0012738g0010	−65	2.25075E-07	0.00058269	Zinc finger, C3HC4 RING-type, Zinc finger, RING-type, Zinc finger, RING/FYVE/PHD-type
Nitab4.5_0008768g0060	−64.94773519	2.65416E-09	1.55219E-05	Unknown
Nitab4.5_0006523g0030	−64.82334869	1.61203E-06	0.002728945	Cyclic nucleotide-binding-like, Cyclic nucleotide-binding domain, Potassium channel, voltage-dependent, EAG/ELK/ERG, IQ motif, EF-hand binding site, Ion transport domain, RmlC-like jelly roll fold
Nitab4.5_0005871g0010	−64.61538462	5.53159E-06	0.006928162	Leucine-rich repeat, cysteine-containing subtype
Nitab4.5_0007776g0020	−64.57489879	2.23493E-08	8.91817E-05	DNA mismatch repair protein MutS, core
Nitab4.5_0000072g0480	−64.56521739	2.28772E-08	9.08614E-05	Methylosome subunit pICln
Nitab4.5_0006523g0030	−64.51612903	4.48406E-08	0.000158124	Cyclic nucleotide-binding-like, Cyclic nucleotide-binding domain, Potassium channel, voltage-dependent, EAG/ELK/ERG, IQ motif, EF-hand binding site, Ion transport domain, RmlC-like jelly roll fold
Nitab4.5_0000464g0090	−64.51612903	6.20495E-11	6.87849E-07	Pentatricopeptide repeat, Tetratricopeptide-like helical
Nitab4.5_0009445g0010	−64.44444444	1.90877E-07	0.000512061	AMP-dependent synthetase/ligase, AMP-binding, conserved site, AMP-binding enzyme C-terminal domain
Nitab4.5_0007165g002	−64.42857143	1.24397E-0	0.000360517	CCAAT-binding factor, conserved site,

0		7		CCAAT-binding transcription factor, subunit B
Nitab4.5_0001904g0120	−64.42687747	7.66008E-07	0.001519042	Protein arginine N-methyltransferase, Protein arginine N-methyltransferase CARM1
Nitab4.5_0001778g0180	−64.28571429	8.18609E-12	1.2499E-07	Adipose-regulatory protein, Seipin
Nitab4.5_0003485g0090	−64.01515152	5.86363E-07	0.00123612	Unknown
Nitab4.5_0000317g0220	−64	9.69251E-08	0.000295763	Zinc finger, CW-type, SET domain, Post-SET domain, AWS
Nitab4.5_0009998g0020	−63.89189189	7.58044E-08	0.000243823	Replication factor C, C-terminal domain, P-loop containing nucleoside triphosphate hydrolase, DNA polymerase III, clamp loader complex, gamma/delta/delta subunit, C-terminal
Nitab4.5_0009755g0010	−63.80952381	4.72325E-07	0.001052134	Sas10/Utp3/C1D
Nitab4.5_0003470g0010	−63.63636364	1.42168E-07	0.000401336	DDT domain superfamily, Zinc finger, PHD-finger, DDT domain, subgroup, Zinc finger, PHD-type, conserved site, Zinc finger, PHD-type, Zinc finger, FYVE/PHD-type, Zinc finger, RING/FYVE/PHD-type, DDT domain
Nitab4.5_0000003g0100	−63.63636364	5.76344E-10	4.36966E-06	Galectin, carbohydrate recognition domain, Concanavalin A-like lectin/glucanase, subgroup, Concanavalin A-like lectin/glucanases superfamily, Glycosyl transferase, family 31
Nitab4.5_0002114g0160	−63.42857143	7.44526E-07	0.00148896	Unknown
Nitab4.5_0002485g0080	−63.10344828	1.69228E-06	0.002832084	Ribosomal protein L2 domain 2, Translation protein SH3-like domain, KOW, Ribosomal protein L24
Nitab4.5_0000189g0260	−62.98701299	1.10425E-06	0.002033845	CCAAT-binding transcription factor, subunit B, CCAAT-binding factor, conserved site
Nitab4.5_0002321g0070	−62.96296296	2.69706E-08	0.000103629	Pyridoxal phosphate-dependent transferase, major region, subdomain 1, Pyridoxal phosphate-dependent transferase, Aminotransferase, class V/Cysteine desulfurase, MOSC, N-terminal beta barrel, Pyridoxal phosphate-dependent transferase, major region, subdomain 2
Nitab4.5_0000090g0250	−62.83333333	2.07447E-06	0.003317388	Dymeclin
Nitab4.5_0000625g0060	−62.82590412	4.70647E-08	0.000163976	Ubiquitin supergroup, HECT, Ubiquitin domain
Nitab4.5_0004317g0030	−62.5	2.40755E-06	0.003713596	Armadillo, Armadillo-like helical, Importin-alpha, importin-beta-binding domain, Armadillo-type fold, Importin subunit alpha
Nitab4.5_0005269g0010	−62.5	1.29796E-07	0.000371848	Protein of unknown function DUF760
Nitab4.5_0007593g001	−62.5	3.07963E-0	0.000116122	DNA primase, UL52/UL70 type,

0		8		Herpesviridae, DNA primase, small subunit
Nitab4.5_0003727g0020	−62.36363636	8.22557E-06	0.009405775	Cytochrome P450, Cytochrome P450, E-class, group IV
Nitab4.5_0002643g0050	−62.31884058	3.53172E-06	0.004956945	Unknown
Nitab4.5_0000104g0050	−62.26086957	7.19777E-06	0.008517237	Annexin repeat, Annexin repeat, conserved site, Annexin, plant, Annexin
Nitab4.5_0005145g0010	−62.22222222	4.5432E-09	2.41417E-05	Uncharacterised protein family UPF0136, Transmembrane
Nitab4.5_0003234g0070	−62.05882353	2.1588E-07	0.000562313	Helicase, C-terminal, SNF2-related, P-loop containing nucleoside triphosphate hydrolase, Helicase, superfamily 1/2, ATP-binding domain
Nitab4.5_0000667g0050	−62.03605514	4.42811E-07	0.001001678	Longin-like domain, Coatomer delta subunit, Clathrin adaptor, mu subunit, C-terminal
Nitab4.5_0004307g0020	−61.9047619	2.68276E-06	0.004017749	Yip1 domain
Nitab4.5_0008542g0010	−61.9047619	1.3784E-06	0.002414322	Unknown
Nitab4.5_0003553g0120	−61.9047619	9.32728E-07	0.001772659	Protein of unknown function DUF761, plant
Nitab4.5_0000269g0100	−61.9047619	1.11571E-07	0.000331955	ABC transporter-like, P-loop containing nucleoside triphosphate hydrolase, AAA+ ATPase domain, ABC transporter A, ABCA, ABC transporter, conserved site
Nitab4.5_0000735g0010	−61.53846154	8.01463E-08	0.000254701	Small GTPase superfamily, Small GTPase superfamily, Rho type, Small GTPase superfamily, Ras type, Small GTPase superfamily, Rab type, P-loop containing nucleoside triphosphate hydrolase, Small GTP-binding protein domain
Nitab4.5_0004234g0130	−61.42857143	1.30011E-06	0.002308635	Nucleotide-binding, alpha-beta plait, RNA recognition motif domain, RNA recognition motif domain, eukaryote
Nitab4.5_0002026g0060	−61.40350877	3.89897E-08	0.000141246	Pentatricopeptide repeat, GYF, Tetratricopeptide-like helical
Nitab4.5_0000215g0090	−61.35338346	4.91309E-08	0.00016907	PHP, C-terminal, Polymerase/histidinol phosphatase-like
Nitab4.5_0002594g0070	−61.17936118	1.45879E-06	0.002524923	F-box domain, Leucine-rich repeat, cysteine-containing subtype
Nitab4.5_0009436g0040	−61.11111111	1.6698E-06	0.002804406	Unknown
Nitab4.5_0000822g0130	−61.09090909	3.93044E-08	0.000142265	Protein of unknown function DUF3531
Nitab4.5_0005254g0010	−61.05072464	2.39382E-06	0.003702559	Ribosomal protein S5 domain 2-type fold, subgroup, Histidine kinase-like ATPase, ATP-binding domain, DNA gyrase, subunit B, DNA topoisomerase, type IIA, conserved site, DNA topoisomerase, type IIA, central domain, Ribosomal protein S5 domain 2-type fold, DNA gyrase B subunit, C-terminal, DNA topoisomerase, type IIA-like

				domain, DNA topoisomerase, type IIA, subunit B, DNA topoisomerase, type IIA, subunit B, domain 2, DNA topoisomerase, type IIA, Toprim domain
Nitab4.5_0000170g0220	−60.86956522	8.44248E-07	0.001637487	Protein kinase-like domain, Serine-threonine/tyrosine-protein kinase catalytic domain, Protein kinase domain
Nitab4.5_0003943g0020	−60.86956522	1.87285E-07	0.000503906	Oxysterol-binding protein, conserved site, Oxysterol-binding protein
Nitab4.5_0015896g0010	−60.86956522	3.49641E-08	0.000128194	EGF-like calcium-binding domain, Protease-associated domain, PA, Complement Clr-like EGF domain, EGF-like calcium-binding, conserved site
Nitab4.5_0008833g0010	−60.86956522	1.39632E-08	6.05559E-05	Protein-tyrosine phosphatase, active site, Dual specificity phosphatase, subgroup, catalytic domain, Dual specificity phosphatase, catalytic domain, Dual specificity phosphatase, Villin/Gelsolin, Protein-tyrosine/Dual specificity phosphatase
Nitab4.5_0005154g0020	−60.77075099	2.64361E-11	3.3405E-07	Aconitase/3-isopropylmalate dehydratase large subunit, alpha/beta/alpha, Aconitase/3-isopropylmalate dehydratase large subunit, alpha/beta/alpha, subdomain 1/3, Aconitase B, iron-sulphur-binding, bacterial, Aconitase/3-isopropylmalate dehydratase large subunit, alpha/beta/alpha, subdomain 2, Homoaconitase/3-isopropylmalate dehydratase, large subunit, Aconitase/isopropylmalate dehydratase
Nitab4.5_0001296g0080	−60.55555556	4.70413E-09	2.48723E-05	CID domain, RNA polymerase II-binding domain, ENTH/VHS
Nitab4.5_0001427g0020	−60.52631579	4.6559E-08	0.000162831	Purine 5'-nucleotidase, HAD-superfamily hydrolase, subfamily IG, 5'-nucleotidase, HAD-like domain
Nitab4.5_0006489g0010	−60.52631579	8.41958E-10	5.95789E-06	Glycosyl transferase, family 2
Nitab4.5_0000170g0260	−60.46153846	4.94067E-06	0.006377863	Galactose oxidase, beta-propeller, Kelch repeat type 1
Nitab4.5_0000246g0160	−60.41055718	4.47844E-07	0.001010325	DJ-1, ThiJ/PfpI, Protein kinase, ATP binding site, Serine/threonine-protein kinase, active site, Protein kinase domain, Serine/threonine- / dual specificity protein kinase, catalytic domain, Protein kinase-like domain
Nitab4.5_0000019g0020	−60.38961039	2.05242E-07	0.000541918	Serine/threonine-protein kinase, active site, Protein kinase-like domain, Serine-threonine/tyrosine-protein kinase catalytic domain, Serine/threonine- / dual specificity protein kinase, catalytic domain, Protein kinase domain
Nitab4.5_0000072g0150	−60.32258065	5.15776E-06	0.00658163	Peptidase M48

Nitab4.5_0002526g0060	−60.27777778	1.49985E-07	0.000420052	Unknown
Nitab4.5_0008556g0020	−60.12725345	1.48974E-06	0.002563859	Pyridine nucleotide-disulphide oxidoreductase, FAD/NAD(P)-binding domain, Pyridine nucleotide-disulphide oxidoreductase, dimerisation domain, FAD-dependent pyridine nucleotide-disulphide oxidoreductase, Pyridine nucleotide-disulphide oxidoreductase, NAD-binding domain, FAD/NAD-linked reductase, dimerisation domain
Nitab4.5_0011089g0020	−60.08658009	2.6762E-12	4.9402E-08	Anaphase-promoting complex subunit 1
Nitab4.5_0009327g0010	−60.06944444	1.27019E-07	0.000365126	2-oxoglutarate dehydrogenase, E1 component, Transketolase-like, pyrimidine-binding domain, Dehydrogenase, E1 component
Nitab4.5_0000227g0300	−60	7.68312E-07	0.0015229	Unknown
Nitab4.5_0002788g0030	−60	2.00857E-07	0.000532964	Cysteine alpha-hairpin motif superfamily
Nitab4.5_0003984g0010	−60	6.64814E-08	0.000218825	Cation/H+ exchanger
Nitab4.5_0008197g0020	−60	1.7535E-08	7.27751E-05	WD40/YVTN repeat-like-containing domain, WD40-repeat-containing domain, Secretory pathway Sec39
Nitab4.5_0000483g0150	−59.9378882	1.96885E-06	0.00319298	Ran GTPase, Small GTPase superfamily, Small GTP-binding protein domain, Small GTPase superfamily, Rab type, Small GTPase superfamily, Ras type, P-loop containing nucleoside triphosphate hydrolase, Small GTPase superfamily, Rho type
Nitab4.5_0001364g0090	−59.86394558	3.13201E-09	1.77303E-05	RNA recognition motif domain, Nucleotide-binding, alpha-beta plait
Nitab4.5_0011195g0030	−59.70695971	1.93372E-07	0.000517008	Protein of unknown function DUF639
Nitab4.5_0006948g0020	−59.45736434	1.8271E-08	7.53146E-05	JmjC domain
Nitab4.5_0008893g0010	−59.44444444	1.10333E-06	0.002033845	Unknown
Nitab4.5_0005191g0050	−59.375	9.30691E-08	0.00028738	Homeobox domain, DDT domain superfamily, DNA-directed RNA polymerase delta subunit/Asxl, Homeodomain-like, DDT domain, subgroup, DDT domain
Nitab4.5_0011089g0020	−59.375	6.76165E-08	0.000221685	Anaphase-promoting complex subunit 1
Nitab4.5_0010206g0030	−59.25925926	1.07709E-06	0.001994349	Unknown
Nitab4.5_0000592g0260	−59.25925926	2.08749E-07	0.000548884	SRA-YDG, SET domain, PUA-like domain, Histone H3-K9 methyltransferase, plant, Pre-SET domain, Pre-SET zinc-binding sub-group
Nitab4.5_0001364g009	−59.25925926	4.72283E-0	0.000164091	RNA recognition motif domain,

0		8		Nucleotide-binding, alpha-beta plait
Nitab4.5_0000438g006 0	−59.09090909	1.15807E-0 6	0.002107529	Endonuclease/exonuclease/phosphatase, AP endonuclease 1, Zinc finger, GRF-type
Nitab4.5_0004207g001 0	−59.09090909	1.15807E-0 6	0.002107529	P-loop containing nucleoside triphosphate hydrolase, ABC transporter-like, AAA+ ATPase domain
Nitab4.5_0002295g003 0	−59.09090909	4.68838E-0 8	0.000163698	F-box domain, Leucine-rich repeat, cysteine-containing subtype
Nitab4.5_0009327g001 0	−59.05567301	2.2607E-08	8.99565E-05	2-oxoglutarate dehydrogenase, E1 component, Transketolase-like, pyrimidine-binding domain, Dehydrogenase, E1 component
Nitab4.5_0003189g001 0	−58.96296296	1.54431E-0 6	0.002637473	Ubiquitin domain, Ubiquitin-associated domain/translation elongation factor EF-Ts, N-terminal, Ubiquitin-associated/translation elongation factor EF1B, N-terminal, eukaryote, Ubiquitin supergroup, UV excision repair protein Rad23, UBA-like, Heat shock chaperonin-binding, XPC-binding domain
Nitab4.5_0010415g004 0	−58.85093168	7.68252E-0 6	0.008965908	Vacuolar protein sorting-associated protein 54, Vps54-like
Nitab4.5_0006794g005 0	−58.67895545	1.05031E-0 6	0.001954995	Enhancer of polycomb-like, N-terminal, Enhancer of polycomb protein
Nitab4.5_0009324g001 0	−58.67208672	1.61814E-0 9	1.02693E-05	EF-hand domain pair, EF-hand domain, EF-Hand 1, calcium-binding site
Nitab4.5_0003391g009 0	−58.42105263	3.27892E-0 8	0.000121691	Autophagy-related protein 27
Nitab4.5_0000486g021 0	−58.38870432	7.59262E-0 9	3.67994E-05	ATP-dependent helicase, C-terminal, P-loop containing nucleoside triphosphate hydrolase, Helicase, superfamily 1/2, ATP-binding domain, DinG/Rad3-type, Helicase-like, DEXD box c2 type, DEAD2
Nitab4.5_0001317g018 0	−58.37320574	3.33846E-0 8	0.000123355	Zinc finger, RING-type, Zinc finger, RING/FYVE/PHD-type
Nitab4.5_0000548g012 0	−58.35294118	4.20037E-0 6	0.005650716	JmjC domain, AT hook, DNA-binding motif
Nitab4.5_0001014g015 0	−58.33333333	6.59281E-0 6	0.007980522	Unknown
Nitab4.5_0000510g017 0	−58.33333333	4.78673E-0 7	0.001061265	Indole-3-glycerol phosphate synthase, Indole-3-glycerol phosphate synthase, conserved site, Aldolase-type TIM barrel, Ribulose-phosphate binding barrel
Nitab4.5_0002020g006 0	−58.33333333	1.13713E-0 8	5.10897E-05	WD40-repeat-containing domain, WD40 repeat, WD40/YVTN repeat-like-containing domain, Synaptobrevin
Nitab4.5_0000880g003 0	−58.33333333	6.65032E-0 9	3.27939E-05	RNA helicase, ATP-dependent, SK12/DOB1, DSH, C-terminal, Helicase, C-terminal, Helicase, superfamily 1/2, ATP-binding domain, rRNA-processing arch domain, P-loop containing nucleoside triphosphate hydrolase,

				DNA/RNA helicase, DEAD/DEAH box type, N-terminal
Nitab4.5_0010033g0140	−58.17524842	5.50813E-07	0.001177547	Unknown
Nitab4.5_0000785g0010	−58.06451613	1.1144E-07	0.000331799	ATM/Tel1, Phosphatidylinositol 3/4-kinase, conserved site, Phosphatidylinositol 3-/4-kinase, catalytic domain, PIK-related kinase, FAT, PIK-related kinase, Protein kinase-like domain, Armadillo-type fold
Nitab4.5_0000661g0020	−58.06451613	4.44046E-07	0.001003355	ATP-citrate lyase/succinyl-CoA ligase, Succinyl-CoA synthetase-like, CoA-binding, NAD(P)-binding domain, Succinyl-CoA ligase, alpha subunit
Nitab4.5_0000246g0160	−58.06451613	1.66989E-08	6.99205E-05	DJ-1, ThiJ/PfpI, Protein kinase, ATP binding site, Serine/threonine-protein kinase, active site, Protein kinase domain, Serine/threonine- / dual specificity protein kinase, catalytic domain, Protein kinase-like domain
Nitab4.5_0000104g0330	−57.81818182	6.11805E-06	0.007531359	Unknown
Nitab4.5_0005574g0040	−57.69230769	4.07051E-07	0.000942329	TAFII-230 TBP-binding, Ubiquitin supergroup, Ubiquitin domain, Bromodomain, Transcription initiation factor TFIID subunit 1, domain of unknown function, Bromodomain, conserved site
Nitab4.5_0001280g0050	−57.38095238	4.2918E-07	0.000977567	Pentatricopeptide repeat, Tetratricopeptide-like helical
Nitab4.5_0001003g0170	−57.35430157	6.28452E-07	0.001306682	Ribosomal protein S5 domain 2-type fold, Ribosomal protein S5 domain 2-type fold, subgroup, Ribosomal protein S5, N-terminal, Ribosomal protein S5, Ribosomal protein S5, C-terminal, Double-stranded RNA-binding domain
Nitab4.5_0000062g0620	−57.14285714	4.86465E-06	0.006304652	Major facilitator superfamily, Major facilitator superfamily domain, Major facilitator superfamily domain, general substrate transporter
Nitab4.5_0003429g0060	−57.14285714	4.86465E-06	0.006304652	RNA recognition motif domain, Nucleotide-binding, alpha-beta plait
Nitab4.5_0001519g0190	−57.14285714	5.15175E-07	0.001119968	Histone acetyltransferases subunit 3
Nitab4.5_0000667g0220	−57.14285714	3.65094E-07	0.000863082	Nonaspanin (TM9SF)
Nitab4.5_0000033g0080	−57.14285714	1.0342E-07	0.000310983	Unknown
Nitab4.5_0000395g0010	−57.14285714	1.57275E-08	6.64433E-05	Unknown
Nitab4.5_0003555g0070	−57.14285714	5.78921E-09	2.94104E-05	Post-SET domain, SET domain, AWS, Histone-lysine N-methyltransferase, SET2, plant
Nitab4.5_0001296g0110	−57.0494186	4.82543E-09	2.53873E-05	Armadillo-like helical, Armadillo-type fold
Nitab4.5_0000573g012	−56.94444444	2.4704E-15	1.45676E-10	LETM1-like

0				
Nitab4.5_0001878g0040	−56.92682927	2.24462E-08	8.94842E-05	Ribosomal protein S19, superfamily, Ribosomal protein S19/S15, Ribosomal protein S19 conserved site, Ribosomal protein S19A/S15e
Nitab4.5_0003555g0030	−56.91056911	3.09883E-06	0.004494992	Zinc finger, RING/FYVE/PHD-type, Zinc finger, SIAH-type, Seven-in-absentia protein, TRAF-like domain, E3 ubiquitin-protein ligase SINA like, Zinc finger, RING-type, TRAF-like, SIAH-type domain
Nitab4.5_0005761g0140	−56.70289855	8.76939E-06	0.009852501	Endonuclease/exonuclease/phosphatase, Reverse transcriptase zinc-binding domain
Nitab4.5_0009617g0020	−56.69856459	2.91711E-07	0.000721752	Cobalamin (vitamin B12)-independent methionine synthase MetE, N-terminal, Cobalamin-independent methionine synthase, Methionine synthase, vitamin-B12 independent
Nitab4.5_0004656g0030	−56.66666667	2.47619E-06	0.003781744	PC-Esterase, PMR5 N-terminal domain
Nitab4.5_0000170g0220	−56.62020906	1.90286E-06	0.003115224	Protein kinase-like domain, Serine-threonine/tyrosine-protein kinase catalytic domain, Protein kinase domain
Nitab4.5_0000573g0120	−56.53235653	1.59761E-07	0.000442736	LETM1-like
Nitab4.5_0000347g0180	−56.52173913	9.93683E-07	0.001866778	Unknown
Nitab4.5_0004479g0030	−56.52173913	3.7608E-07	0.000883639	Glutaredoxin, Glutaredoxin subgroup, Glutaredoxin active site, Thioredoxin-like fold, Glutaredoxin, eukaryotic/virial
Nitab4.5_0002565g0080	−56.52173913	2.04457E-07	0.000540182	SWIRM domain, Homeodomain-like, Amine oxidase, NAD(P)-binding domain, Winged helix-turn-helix DNA-binding domain
Nitab4.5_0005567g0020	−56.49717514	8.59401E-08	0.000269284	SWIRM domain, Amine oxidase, Winged helix-turn-helix DNA-binding domain, Homeodomain-like, Transcription factor IIS, N-terminal
Nitab4.5_0000391g0250	−56.38820639	3.99968E-07	0.000929556	Sec7 domain, Sec7 domain, alpha orthogonal bundle
Nitab4.5_0000625g0060	−56.23136553	1.60827E-07	0.000444839	Ubiquitin supergroup, HECT, Ubiquitin domain
Nitab4.5_0000126g0070	−56	4.09679E-07	0.000946648	Unknown
Nitab4.5_0000003g0490	−56	2.07546E-08	8.39227E-05	Unknown
Nitab4.5_0002526g0060	−55.97433841	1.17413E-06	0.002134002	Unknown
Nitab4.5_0000303g0060	−55.88235294	2.34402E-08	9.25778E-05	Dehydroquinase class I, Aldolase-type TIM barrel, NAD(P)-binding domain, Shikimate dehydrogenase, Quinate/shikimate 5-dehydrogenase/glutamyl-tRNA reductase, Shikimate dehydrogenase substrate binding, N-terminal,

				Shikimate, quinate/shikimate dehydrogenase
Nitab4.5_0001369g0070	−55.88235294	2.34402E-08	9.25778E-05	Unknown
Nitab4.5_0001797g0080	−55.86622807	1.38066E-09	9.07421E-06	Peptidase S54, rhomboid domain, Peptidase S54, rhomboid
Nitab4.5_0010703g0040	−55.72801183	4.71058E-08	0.000163976	Unknown
Nitab4.5_0007282g0070	−55.70175439	2.3043E-07	0.000593656	Domain of unknown function DUF629, Ubiquitin carboxyl-terminal hydrolases family 2, Zinc finger, C2H2
Nitab4.5_0000573g0160	−55.55555556	2.53194E-06	0.003846657	Tetratricopeptide TPR1, Tetratricopeptide repeat, Tetratricopeptide-like helical, Tetratricopeptide repeat-containing domain
Nitab4.5_0001114g0100	−55.55555556	2.35779E-07	0.000601988	Conserved oligomeric Golgi complex, subunit 3, Cullin repeat-like-containing domain
Nitab4.5_0001000g0120	−55.55555556	2.36724E-09	1.41161E-05	Pentatricopeptide repeat
Nitab4.5_0005039g0030	−55.33769063	2.71958E-08	0.000104306	Diaminopimelate epimerase, DapF, Diaminopimelate epimerase, active site
Nitab4.5_0002682g0030	−55.19230769	1.0225E-07	0.000308977	DNA/RNA helicase, DEAD/DEAH box type, N-terminal, Sec63 domain, P-loop containing nucleoside triphosphate hydrolase, Helicase, superfamily 1/2, ATP-binding domain, Helicase, C-terminal
Nitab4.5_0000010g0010	−55.17241379	1.21244E-06	0.002185858	Zinc finger, RanBP2-type
Nitab4.5_0006344g0030	−55.17241379	8.23992E-09	3.9397E-05	Nuclear transport factor 2, RNA recognition motif domain, Nucleotide-binding, alpha-beta plait, Nuclear transport factor 2, eukaryote
Nitab4.5_0001500g0030	−55.16483516	3.55597E-06	0.004981098	Pentatricopeptide repeat
Nitab4.5_0003566g0010	−55.15521064	1.65222E-07	0.000454626	Unknown
Nitab4.5_0004158g0020	−55	5.15776E-06	0.00658163	Immunoglobulin E-set
Nitab4.5_0006948g0020	−54.99108734	2.47277E-06	0.00378061	JmjC domain
Nitab4.5_0008522g0010	−54.9860205	5.06891E-06	0.006497541	WD40/YVTN repeat-like-containing domain, Glutamine cyclotransferase
Nitab4.5_0008042g0040	−54.9689441	1.63466E-08	6.89125E-05	PAP/25A-associated, D-isomer specific 2-hydroxyacid dehydrogenase, catalytic domain, NAD(P)-binding domain, D-isomer specific 2-hydroxyacid dehydrogenase, NAD-binding
Nitab4.5_0003970g0050	−54.83870968	1.05559E-06	0.001963106	Glutamine-Leucine-Glutamine, QLQ, WRC
Nitab4.5_0000807g0150	−54.83870968	7.09648E-07	0.001434753	RNA recognition motif domain, Nucleotide-binding, alpha-beta plait
Nitab4.5_0000822g0130	−54.80769231	3.10903E-07	0.000759065	Protein of unknown function DUF3531
Nitab4.5_0001107g016	−54.62573591	3.56419E-0	0.000130117	WD40 repeat, WD40 repeat, conserved

0		8		site, G-protein beta WD-40 repeat, WD40-repeat-containing domain, WD40/YVTN repeat-like-containing domain
Nitab4.5_0009429g0030	−54.54545455	5.65418E-06	0.007037951	Unknown
Nitab4.5_0009766g0010	−54.54545455	5.65418E-06	0.007037951	MCM N-terminal domain, Mini-chromosome maintenance, DNA-dependent ATPase, P-loop containing nucleoside triphosphate hydrolase, Nucleic acid-binding, OB-fold
Nitab4.5_0000102g0270	−54.54545455	1.14484E-06	0.002089074	Pumilio RNA-binding repeat, Nucleic acid binding NABP, Armadillo-like helical, Armadillo-type fold
Nitab4.5_0000650g0210	−54.54545455	2.15163E-07	0.000561824	AAA+ ATPase domain, TIP49, C-terminal, P-loop containing nucleoside triphosphate hydrolase, RuvB-like, Nucleic acid-binding, OB-fold
Nitab4.5_0001625g0010	−54.54545455	6.25944E-08	0.000207692	Sec-independent protein translocase protein TatA/E, Sec-independent protein translocase protein TatA/B/E
Nitab4.5_0005345g0040	−54.54545455	1.09323E-08	4.92734E-05	FMP27, GFWDK domain, FMP27, C-terminal
Nitab4.5_0000185g0290	−54.44444444	6.93886E-07	0.001412979	Transposase, MuDR, plant
Nitab4.5_0003827g0050	−54.32950192	1.90919E-07	0.000512061	Unknown
Nitab4.5_0003026g0030	−54.28571429	1.95195E-06	0.003171637	Ribosomal protein L23/L15e core domain, Ribosomal protein L25/L23, Nucleotide-binding, alpha-beta plait
Nitab4.5_0006591g0020	−54.28571429	1.68578E-06	0.002823418	CMP/dCMP deaminase, zinc-binding, Cytidine deaminase-like
Nitab4.5_0013567g0010	−54.28571429	8.24489E-07	0.001608715	Pentatricopeptide repeat, Tetratricopeptide-like helical
Nitab4.5_0002796g0080	−54.28571429	1.85378E-08	7.6266E-05	Protein of unknown function DUF2045
Nitab4.5_0013867g0020	−54.21245421	5.88005E-06	0.007284404	Ubiquitin-fold modifier 1
Nitab4.5_0006105g0010	−54.20168067	4.2664E-07	0.000973871	Zinc finger, RING/FYVE/PHD-type, Zinc finger, PHD-finger, Alfin, Zinc finger, PHD-type, Zinc finger, FYVE/PHD-type, Zinc finger, PHD-type, conserved site
Nitab4.5_0002408g0040	−54.16666667	8.81189E-08	0.00027475	Phox homologous domain
Nitab4.5_0000784g0140	−54.03508772	1.04174E-07	0.000312796	GTP1/OBG domain, GTP1/OBG, conserved site, GTP-binding protein GTP1/OBG, C-terminal, GTP-binding protein Obg/CgtA, GTP binding domain, P-loop containing nucleoside triphosphate hydrolase
Nitab4.5_0000573g0160	−54.004914	4.50962E-09	2.40234E-05	Tetratricopeptide TPR1, Tetratricopeptide repeat, Tetratricopeptide-like helical, Tetratricopeptide repeat-containing

				domain
Nitab4.5_0001746g0050	−53.71158392	1.66256E-08	6.98178E-05	Polyadenylate-binding protein/Hyperplastic disc protein, RNA recognition motif domain, Nucleotide-binding, alpha-beta plait
Nitab4.5_0005682g0010	−53.65418895	1.71431E-09	1.0783E-05	Unknown
Nitab4.5_0000129g0400	−53.63636364	8.35546E-06	0.009513297	Ankyrin repeat-containing domain, Ankyrin repeat
Nitab4.5_0000486g0210	−53.57142857	6.97337E-07	0.001418645	ATP-dependent helicase, C-terminal, P-loop containing nucleoside triphosphate hydrolase, Helicase, superfamily 1/2, ATP-binding domain, DinG/Rad3-type, Helicase-like, DEXD box c2 type, DEAD2
Nitab4.5_0000464g0030	−53.57142857	8.81108E-09	4.152E-05	Zinc finger, PMZ-type, MULE transposase domain, Zinc finger, SWIM-type, FAR1 DNA binding domain
Nitab4.5_0000586g0070	−53.43915344	1.59985E-06	0.002712686	Villin headpiece, Villin/Gelsolin, Gelsolin domain
Nitab4.5_0002027g0100	−53.33333333	8.79444E-07	0.00169007	Peptidase C13, legumain
Nitab4.5_0001364g0090	−53.33333333	6.13102E-07	0.001281798	RNA recognition motif domain, Nucleotide-binding, alpha-beta plait
Nitab4.5_0014337g0010	−53.33333333	1.56448E-07	0.000434711	Target SNARE coiled-coil domain, Syntaxin, N-terminal domain, t-SNARE, Syntaxin/epimorphin, conserved site
Nitab4.5_0001467g0130	−53.125	3.55792E-06	0.004982185	Protein kinase domain, KEN domain, Quinonprotein alcohol dehydrogenase-like domain, Quinonprotein alcohol dehydrogenase-like superfamily, PUG domain, Serine/threonine- / dual specificity protein kinase, catalytic domain, Protein kinase-like domain
Nitab4.5_0000954g0020	−52.94117647	6.25713E-07	0.001302262	Prenylcysteine lyase
Nitab4.5_0009327g0010	−52.77777778	1.06967E-06	0.001984064	2-oxoglutarate dehydrogenase, E1 component, Transketolase-like, pyrimidine-binding domain, Dehydrogenase, E1 component
Nitab4.5_0006772g0020	−52.63157895	4.75402E-08	0.000164905	CID domain, PWWP domain, ENTH/VHS
Nitab4.5_0001687g0080	−52.38095238	6.21458E-06	0.007621457	Importin-beta, N-terminal domain, Armadillo-like helical, Armadillo-type fold, Exportin/Importin, Cse1-like
Nitab4.5_0001296g0080	−52.32919255	3.42983E-06	0.004862156	CID domain, RNA polymerase II-binding domain, ENTH/VHS
Nitab4.5_0002167g0040	−52.32323232	1.95079E-08	7.94871E-05	Cytochrome b-c1 complex subunit 8, plants, Cytochrome b-c1 complex subunit 8
Nitab4.5_0000051g0030	−52.23880597	1.51797E-12	3.11348E-08	Unknown
Nitab4.5_0004532g0030	−52.17391304	4.68128E-06	0.006140069	Pentatricopeptide repeat, Tetratricopeptide-like helical
Nitab4.5_0000073g040	−52.17391304	2.88502E-0	0.000715064	Sucrose synthase, Sucrose synthase,

0		7		plant/cyanobacteria, Glycosyl transferase, family 1
Nitab4.5_0001907g005 0	−52	3.45249E-0 8	0.000127022	SWIB/MDM2 domain, Plus-3 domain, subgroup, Plus-3
Nitab4.5_0001362g004 0	−51.85185185	5.79037E-0 6	0.007190525	Protein of unknown function DUF1077, TMEM85
Nitab4.5_0000827g006 0	−51.85185185	1.34538E-0 7	0.000384396	AMP-dependent synthetase/ligase, AMP-binding, conserved site
Nitab4.5_0000688g009 0	−51.80533752	5.86467E-0 8	0.000196993	GDP-fucose protein O-fucosyltransferase, O-fucosyltransferase, plant
Nitab4.5_0002021g003 0	−51.76767677	2.37165E-0 6	0.003684386	Unknown
Nitab4.5_0001393g014 0	−51.61290323	6.59758E-0 7	0.001355838	Endoribonuclease L-PSP/chorismate mutase-like, YjgF/Yer057p/UK114 family
Nitab4.5_0001456g017 0	−51.51515152	5.33566E-0 7	0.00114994	Pentatricopeptide repeat
Nitab4.5_0000038g026 0	−51.35135135	3.93397E-0 6	0.005374071	FAR1 DNA binding domain
Nitab4.5_0019126g002 0	−51.3368984	1.25485E-0 7	0.00036268	WD40/YVTN repeat-like-containing domain
Nitab4.5_0008943g001 0	−51.28205128	6.68925E-0 7	0.001371358	Vacuolar protein sorting-associated protein 35, Armadillo-type fold
Nitab4.5_0001677g005 0	−51.10741971	1.30425E-0 8	5.71465E-05	Acetolactate synthase, small subunit, Acetolactate synthase, small subunit, C-terminal
Nitab4.5_0000136g025 0	−50.6993007	3.6595E-08	0.000133367	Pyruvate/Phosphoenolpyruvate kinase-like domain, Uncharacterised protein family UPF0261, TIM-barrel domain, IGPS-like, Aldolase-type TIM barrel
Nitab4.5_0001296g008 0	−50.65359477	1.0232E-07	0.000308977	CID domain, RNA polymerase II-binding domain, ENTH/VHS
Nitab4.5_0000827g013 0	−50.37537538	5.08339E-0 6	0.006506711	Peptidase C2, calpain, large subunit, domain III, Cysteine peptidase, cysteine active site, Concanavalin A-like lectin/glucanases superfamily, Peptidase C2, calpain, catalytic domain, Peptidase C2, calpain, domain III, Concanavalin A-like lectin/glucanase, subgroup, Peptidase C2, calpain family
Nitab4.5_0002499g002 0	−50.08403361	2.44059E-1 1	3.10242E-07	Pentatricopeptide repeat, Tetratricopeptide-like helical
Nitab4.5_0003679g002 0	−50	8.53473E-0 6	0.00964499	RNA polymerase II-associated protein 1, N-terminal, RNA polymerase II-associated protein 1, C-terminal, Armadillo-type fold
Nitab4.5_0001000g012 0	−50	6.14E-06	0.007547429	Pentatricopeptide repeat
Nitab4.5_0006741g001 0	−50	3.96511E-0 6	0.005398191	WRC, Glutamine-Leucine-Glutamine, QLQ
Nitab4.5_0005495g003 0	−50	7.25647E-0 7	0.001458763	U3 small nucleolar RNA-associated protein 10
Nitab4.5_0000404g019 0	−50	5.12451E-0 7	0.001116741	Pentatricopeptide repeat
Nitab4.5_0008235g002 0	−50	3.65664E-0 7	0.000863947	Protein of unknown function DUF1664

Nitab4.5_0002567g0020	−50	8.86201E-08	0.000276051	Short-chain dehydrogenase/reductase, conserved site, NAD(P)-binding domain, Short-chain dehydrogenase/reductase SDR, Glucose/ribitol dehydrogenase
Nitab4.5_0000428g0060	−50	7.57978E-08	0.000243823	Nuclear control of ATP synthase 2
Nitab4.5_0003065g0030	−50	5.73441E-08	0.000193229	RNA polymerase Rpb1, domain 1
Nitab4.5_0003566g0020	−50	5.12445E-08	0.000174615	RNA-binding S4 domain, Ribosomal protein S4/S9, eukaryotic/archaeal, Ribosomal protein S4, conserved site, Ribosomal protein S4/S9, Ribosomal protein S4/S9, N-terminal
Nitab4.5_0005567g0020	−50	2.93597E-10	2.4783E-06	SWIRM domain, Amine oxidase, Winged helix-turn-helix DNA-binding domain, Homeodomain-like, Transcription factor IIS, N-terminal
Nitab4.5_0000464g0090	−50	3.78035E-11	4.59897E-07	Pentatricopeptide repeat, Tetratricopeptide-like helical
Nitab4.5_0006136g0020	−49.80694981	5.91672E-06	0.007319591	Aminoacyl-tRNA synthetase, class I, conserved site, Rossmann-like alpha/beta/alpha sandwich fold, Tryptophan-tRNA ligase, Aminoacyl-tRNA synthetase, class Ic
Nitab4.5_0000429g0010	−49.47817837	2.95236E-06	0.004335852	Donson
Nitab4.5_0005682g0010	−49.36507937	9.64964E-08	0.000295385	Unknown
Nitab4.5_0024579g0010	−49.26739927	5.49967E-07	0.00117633	RNA recognition motif domain, Nucleotide-binding, alpha-beta plait
Nitab4.5_0001296g0080	−49.19148936	9.35547E-09	4.36973E-05	CID domain, RNA polymerase II-binding domain, ENTH/VHS
Nitab4.5_0000578g0060	−48.98989899	2.5135E-06	0.003824965	DNA/RNA-binding protein Kin17, conserved domain
Nitab4.5_0000907g0060	−48.875	7.60858E-06	0.008908998	Helicase, superfamily 1/2, ATP-binding domain, DNA helicase, ATP-dependent, RecQ type, P-loop containing nucleoside triphosphate hydrolase, DNA/RNA helicase, DEAD/DEAH box type, N-terminal, Helicase, C-terminal
Nitab4.5_0003343g0030	−48.7804878	4.25565E-07	0.00097194	RNA recognition motif domain, Nucleotide-binding, alpha-beta plait
Nitab4.5_0001863g0050	−48.7804878	1.54791E-08	6.55891E-05	F-box domain, 4Fe-4S ferredoxin, iron-sulphur binding, conserved site
Nitab4.5_0004656g0030	−48.71794872	1.0639E-06	0.001976427	PC-Esterase, PMR5 N-terminal domain
Nitab4.5_0004665g0030	−48.71794872	1.15056E-08	5.15838E-05	Protein kinase domain, Serine-threonine/tyrosine-protein kinase catalytic domain, Protein kinase-like domain, Serine/threonine-protein kinase, active site, Protein kinase, ATP binding site, Serine/threonine- / dual specificity protein kinase, catalytic domain
Nitab4.5_0000957g0040	−48.67401061	8.35018E-07	0.001624779	Protein of unknown function DUF872, transmembrane

Nitab4.5_0001364g0090	−48.46335697	1.25304E-07	0.000362402	RNA recognition motif domain, Nucleotide-binding, alpha-beta plait
Nitab4.5_0000098g0220	−48.27586207	9.2917E-08	0.000287119	HRDC domain, Ribonuclease H-like domain, 3'-5' exonuclease domain, Exosome-associated factor Rrp6, N-terminal, HRDC-like
Nitab4.5_0000573g0120	−48.27586207	3.03824E-09	1.73149E-05	LETM1-like
Nitab4.5_0002611g0040	−48.14814815	1.14361E-06	0.002089074	Pentatricopeptide repeat, Tetratricopeptide-like helical
Nitab4.5_0005079g0050	−48.14814815	2.31421E-07	0.000595487	RNA recognition motif domain, Nucleotide-binding, alpha-beta plait
Nitab4.5_0000573g0150	−48.14814815	2.57478E-08	9.98342E-05	Vacuolar protein sorting 55
Nitab4.5_0012553g0020	−48	2.61122E-06	0.003946742	Ribonuclease H2, subunit B
Nitab4.5_0000458g0070	−47.82608696	2.34214E-06	0.003647882	Armadillo-type fold, Armadillo, BTB/POZ-like, BTB/POZ, Armadillo-like helical, BTB/POZ fold
Nitab4.5_0003364g0080	−47.78554779	3.95897E-06	0.005398191	Tetratricopeptide-like helical, Tetratricopeptide repeat, Tetratricopeptide TPR1, Tetratricopeptide repeat-containing domain
Nitab4.5_0001668g0060	−47.63157895	1.05276E-07	0.00031566	Bromo adjacent homology (BAH) domain, Tudor-like, plant, Agenet-like domain
Nitab4.5_0002210g0050	−47.61904762	1.02653E-06	0.001916644	Aconitase/3-isopropylmalate dehydratase large subunit, alpha/beta/alpha, Aconitase B, iron-sulphur-binding, bacterial, Aconitase/3-isopropylmalate dehydratase large subunit, alpha/beta/alpha, subdomain 1/3, Aconitase/isopropylmalate dehydratase, Aconitase/3-isopropylmalate dehydratase large subunit, alpha/beta/alpha, subdomain 2
Nitab4.5_0000486g0030	−47.61904762	8.41757E-07	0.001634147	Aspartate/other aminotransferase, Aminotransferases, class-I, pyridoxal-phosphate-binding site, Pyridoxal phosphate-dependent transferase, major region, subdomain 1, Pyridoxal phosphate-dependent transferase, major region, subdomain 2, Aminotransferase, class I/classII, Pyridoxal phosphate-dependent transferase
Nitab4.5_0000350g0060	−47.5	4.4198E-06	0.005889933	Phosphatidyl serine synthase
Nitab4.5_0004234g0130	−47.5	3.04836E-06	0.004430864	Nucleotide-binding, alpha-beta plait, RNA recognition motif domain, RNA recognition motif domain, eukaryote
Nitab4.5_0005249g0060	−47.37762238	2.1606E-06	0.003430563	Vacuolar import/degradation, Vid27-related, WD40-repeat-containing domain, WD40/YVTN repeat-like-containing domain

Nitab4.5_0006772g0020	−47.37237237	2.39186E-06	0.003702559	CID domain, PWWP domain, ENTH/VHS
Nitab4.5_0007416g0050	−47.36842105	1.7901E-06	0.00296771	Unknown
Nitab4.5_0011089g0020	−47.36842105	9.14804E-10	6.39871E-06	Anaphase-promoting complex subunit 1
Nitab4.5_0006120g0070	−47.25274725	2.67708E-06	0.004013487	NB-ARC, P-loop containing nucleoside triphosphate hydrolase
Nitab4.5_0019126g0020	−47.14285714	1.92174E-06	0.003138162	WD40/YVTN repeat-like-containing domain
Nitab4.5_0000577g0100	−46.875	9.33001E-09	4.36564E-05	Zinc finger, CCCH-type
Nitab4.5_0001296g0080	−46.42857143	2.39094E-06	0.003702559	CID domain, RNA polymerase II-binding domain, ENTH/VHS
Nitab4.5_0005682g0010	−46.2962963	7.73211E-12	1.1997E-07	Unknown
Nitab4.5_0001296g0110	−46.15384615	5.63005E-07	0.001196383	Armadillo-like helical, Armadillo-type fold
Nitab4.5_0005011g0040	−46	3.61479E-07	0.000857094	RNA-dependent RNA polymerase, eukaryotic-type, Nucleotide-binding, alpha-beta plait, RNA recognition motif domain
Nitab4.5_0007523g0010	−46	2.08915E-07	0.000548884	Mediator complex, subunit Med17
Nitab4.5_0004849g0020	−45.74468085	6.19712E-08	0.000206525	Endonuclease/exonuclease/phosphatase
Nitab4.5_0005485g0010	−45.73205201	2.16997E-13	6.22509E-09	Mannose-binding lectin, NB-ARC, P-loop containing nucleoside triphosphate hydrolase, Disease resistance protein
Nitab4.5_0004234g0130	−45.65217391	3.3878E-08	0.000124858	Nucleotide-binding, alpha-beta plait, RNA recognition motif domain, RNA recognition motif domain, eukaryote
Nitab4.5_0000692g0180	−45.61403509	1.78384E-12	3.45831E-08	Pentatricopeptide repeat, Tetratricopeptide-like helical
Nitab4.5_0000573g0120	−45.58399423	5.42394E-10	4.14184E-06	LETM1-like
Nitab4.5_0002526g0040	−45.45454545	7.10337E-06	0.008436094	Tetratricopeptide repeat, RNA-processing protein, HAT helix, Tetratricopeptide-like helical, Tetratricopeptide repeat-containing domain, Pre-mRNA-processing factor 6/Prp1, PRP1 splicing factor, N-terminal
Nitab4.5_0000731g0050	−45.45454545	1.10271E-08	4.96481E-05	Mitochondrial fission ELM1-like
Nitab4.5_0000316g0260	−45.45454545	7.68035E-09	3.71754E-05	Pentatricopeptide repeat, Tetratricopeptide-like helical
Nitab4.5_0029014g0010	−45.41062802	2.64518E-10	2.29199E-06	Protein-tyrosine phosphatase, receptor/non-receptor type, Protein-tyrosine/Dual specificity phosphatase, Protein-tyrosine phosphatase, catalytic
Nitab4.5_0002465g0060	−45.29100529	8.77547E-06	0.009852501	Glycosyl transferase, family 43
Nitab4.5_0001181g0080	−45.06097561	3.80542E-07	0.000892379	Toprim domain, Twinkle protein
Nitab4.5_0001296g008	−45.04283966	3.83552E-0	0.000139066	CID domain, RNA polymerase

0		8		II-binding domain, ENTH/VHS
Nitab4.5_0007794g0010	−44.68085106	6.29622E-08	0.000208519	Unknown
Nitab4.5_0001802g0080	−44.68085106	4.02472E-10	3.23023E-06	Sas10 C-terminal domain, Sas10/Utp3/C1D
Nitab4.5_0006966g0020	−44.20168067	4.71439E-06	0.006172082	GDP-fucose protein O-fucosyltransferase
Nitab4.5_0005039g0030	−44.13177763	2.32456E-06	0.003626432	Diaminopimelate epimerase, DapF, Diaminopimelate epimerase, active site
Nitab4.5_0000162g0030	−44	6.63086E-06	0.008007081	Domain of unknown function DUF1981, Sec7 associated, Armadillo-type fold, Armadillo-like helical
Nitab4.5_0010831g0040	−44	6.63086E-06	0.008007081	RNA-binding protein Lupus La, Winged helix-turn-helix DNA-binding domain
Nitab4.5_0006686g0010	−44	1.73086E-06	0.002885276	CBS domain, Domain of unknown function DUF21
Nitab4.5_0003364g0020	−44	4.03357E-07	0.000935307	Tetratricopeptide-like helical
Nitab4.5_0002558g0020	−43.75	2.93434E-06	0.004319843	Transcription factor, SBP-box
Nitab4.5_0002964g0020	−43.47826087	6.76862E-06	0.008134108	Unknown
Nitab4.5_0000177g0050	−43.47826087	1.67455E-06	0.002809294	Prefoldin, subunit 3, Prefoldin, Prefoldin alpha-like
Nitab4.5_0009050g0010	−43.33333333	5.45877E-06	0.006879349	Spatacsin
Nitab4.5_0001023g0040	−43.33333333	3.24681E-06	0.004653417	Formin, FH2 domain, Formin-like family, viridiplantae, Tensin phosphatase, C2 domain, C2 domain
Nitab4.5_0007976g0030	−43.32688588	5.8205E-06	0.00721949	Unknown
Nitab4.5_0001554g0040	−42.85714286	5.39034E-06	0.00680118	Zinc finger, RING-type, Zinc finger, RING/FYVE/PHD-type
Nitab4.5_0005176g0070	−42.85714286	6.55557E-07	0.001350815	HD domain, HD/PDEase domain
Nitab4.5_0008957g0040	−42.85714286	5.3508E-07	0.001152387	MIF4-like, type 1/2/3, Armadillo-type fold, MIF4G-like, type 2, MIF4G-like, type 1, Nuclear cap-binding protein subunit 1
Nitab4.5_0000688g0090	−42.60249554	2.70615E-08	0.000103884	GDP-fucose protein O-fucosyltransferase, O-fucosyltransferase, plant
Nitab4.5_0003798g0070	−42.55319149	1.25343E-06	0.002240738	NB-ARC, P-loop containing nucleoside triphosphate hydrolase, Disease resistance protein
Nitab4.5_0000128g0170	−42.32758621	6.40813E-06	0.007811436	Serine hydrolase FSH
Nitab4.5_0013867g0020	−42	8.62102E-06	0.009717292	Ubiquitin-fold modifier 1
Nitab4.5_0001915g0140	−41.86046512	9.59873E-09	4.46861E-05	UV radiation resistance protein/autophagy-related protein 14
Nitab4.5_0002561g0080	−41.84343434	1.65208E-06	0.002779571	ER membrane protein complex subunit 6
Nitab4.5_0008996g0010	−41.66666667	4.36372E-06	0.005829833	Mediator complex, subunit Med14
Nitab4.5_0002341g005	−41.66666667	2.47944E-0	0.003784004	Pentatricopeptide repeat

0		6		
Nitab4.5_0003918g0040	−41.66666667	1.72996E-06	0.002884898	P-loop containing nucleoside triphosphate hydrolase, Sec63 domain, Helicase, C-terminal, Helicase, superfamily 1/2, ATP-binding domain, DNA/RNA helicase, DEAD/DEAH box type, N-terminal, Immunoglobulin E-set
Nitab4.5_0002344g0130	−41.66666667	1.03173E-06	0.001924634	Putative S-adenosyl-L-methionine-dependent methyltransferase
Nitab4.5_0001467g0040	−41.66666667	6.31042E-07	0.00130993	DNA mismatch repair protein, Histidine kinase-like ATPase, ATP-binding domain, Ribosomal protein S5 domain 2-type fold, subgroup
Nitab4.5_0000742g0190	−41.32104455	7.01778E-06	0.008357833	Helicase, C-terminal, Nucleic acid-binding, OB-fold, ATP-dependent DNA helicase RecG, Helicase, superfamily 1/2, ATP-binding domain, DNA/RNA helicase, DEAD/DEAH box type, N-terminal, P-loop containing nucleoside triphosphate hydrolase
Nitab4.5_0001988g0050	−41.1622276	6.10437E-09	3.06354E-05	Filament-like plant protein
Nitab4.5_0007261g0010	−40.90909091	1.53772E-06	0.002632589	Aminotransferase, class I/classII, Pyridoxal phosphate-dependent transferase, major region, subdomain 2, Pyridoxal phosphate-dependent transferase, major region, subdomain 1, Pyridoxal phosphate-dependent transferase, Histidinol-phosphate aminotransferase family
Nitab4.5_0000692g0180	−40.78557977	2.27598E-09	1.36431E-05	Pentatricopeptide repeat, Tetratricopeptide-like helical
Nitab4.5_0000428g0060	−40.74074074	1.35954E-06	0.002390173	Nuclear control of ATP synthase 2
Nitab4.5_0004198g0030	−40.625	2.9906E-06	0.004360333	Unknown
Nitab4.5_0002417g0010	−40.61771562	2.47498E-06	0.003781255	Peptidase M24A, methionine aminopeptidase, subfamily 2, Peptidase M24, structural domain, Peptidase M24, methionine aminopeptidase, Peptidase M24A, methionine aminopeptidase, subfamily 2, binding site, Winged helix-turn-helix DNA-binding domain
Nitab4.5_0006926g0010	−40.35087719	1.31037E-08	5.73557E-05	Exocyst complex component Sec10-like
Nitab4.5_0001833g0080	−40	3.57213E-06	0.004995493	Clathrin light chain
Nitab4.5_0000402g0120	−40	1.08923E-07	0.000325446	WD40-repeat-containing domain, WD40 repeat, conserved site, WD40/YVTN repeat-like-containing domain, WD40 repeat
Nitab4.5_0011375g0020	−40	7.4434E-08	0.000240142	NADH:ubiquinone oxidoreductase, iron-sulphur subunit 5
Nitab4.5_0000159g0050	−40	3.1952E-08	0.000119314	Plant organelle RNA recognition domain
Nitab4.5_0003956g002	−40	4.88497E-0	2.5637E-05	Bromo adjacent homology (BAH)

0		9		domain
Nitab4.5_0000688g0090	−40	6.98396E-10	5.13011E-06	GDP-fucose protein O-fucosyltransferase, O-fucosyltransferase, plant
Nitab4.5_0007944g0030	−38.7636612	6.92961E-06	0.008276036	Unknown
Nitab4.5_0001296g0080	−38.63636364	2.16999E-08	8.66717E-05	CID domain, RNA polymerase II-binding domain, ENTH/VHS
Nitab4.5_0002067g0020	−38.46153846	3.54409E-06	0.004969384	Nucleotide-binding, alpha-beta plait, RNA recognition motif domain
Nitab4.5_0000021g0400	−38.46153846	1.33156E-06	0.002349731	Unknown
Nitab4.5_0008519g0010	−38.46153846	9.80307E-07	0.001846553	Ubiquitin-conjugating enzyme, E2, Ubiquitin-conjugating enzyme/RWD-like, Ubiquitin-conjugating enzyme, active site
Nitab4.5_0018759g0010	−38.23529412	1.87866E-07	0.000505148	GRIM-19
Nitab4.5_0000051g0030	−38.0952381	5.3453E-10	4.09652E-06	Unknown
Nitab4.5_0002677g0020	−37.93103448	2.3513E-07	0.000601988	RNA recognition motif domain, Nucleotide-binding, alpha-beta plait, Nuclear cap-binding protein subunit 2
Nitab4.5_0000355g0030	−37.83783784	8.0982E-07	0.001586296	Nuclear transport factor 2, Nucleotide-binding, alpha-beta plait, RNA recognition motif domain, Nuclear transport factor 2, eukaryote
Nitab4.5_0003259g0030	−37.63864043	7.1721E-06	0.008496338	DNA-binding pseudobarrel domain, Protein of unknown function DUF295, B3 DNA binding domain
Nitab4.5_0000172g1010	−37.51263903	8.06871E-06	0.009275928	Unknown
Nitab4.5_0000931g0010	−37.5	6.80835E-06	0.008170289	FMN-binding split barrel
Nitab4.5_0011177g0030	−37.28617661	6.64864E-08	0.000218825	Unknown
Nitab4.5_0001875g0030	−37.25146199	5.09126E-06	0.006514812	Phox homologous domain, Vps5 C-terminal
Nitab4.5_0001925g0180	−37.14810282	7.6384E-06	0.008934064	EF-Hand 1, calcium-binding site, EF-hand domain pair, EF-hand domain
Nitab4.5_0004033g0090	−37.14810282	7.6384E-06	0.008934064	Unknown
Nitab4.5_0008073g0010	−37.12587413	5.7645E-09	2.9346E-05	Cytochrome P450, conserved site, Cytochrome P450, E-class, group I, Cytochrome P450
Nitab4.5_0005485g0010	−37.0726817	1.76558E-07	0.000478615	Mannose-binding lectin, NB-ARC, P-loop containing nucleoside triphosphate hydrolase, Disease resistance protein
Nitab4.5_0001114g0100	−36.66666667	1.30356E-06	0.002312814	Conserved oligomeric Golgi complex, subunit 3, Cullin repeat-like-containing domain
Nitab4.5_0005039g0030	−36.66666667	1.10931E-06	0.002040199	Diaminopimelate epimerase, DapF, Diaminopimelate epimerase, active site
Nitab4.5_0000573g0140	−36.66666667	8.55476E-08	0.00026845	Domain X, Reverse transcriptase

Nitab4.5_0000688g0090	−36.66666667	8.55476E-08	0.00026845	GDP-fucose protein O-fucosyltransferase, O-fucosyltransferase, plant
Nitab4.5_0001525g0030	−36.58536585	3.18163E-07	0.000772789	TIM-barrel domain, IGPS-like, Aldolase-type TIM barrel, Pyruvate/Phosphoenolpyruvate kinase-like domain, Uncharacterised protein family UPF0261
Nitab4.5_0000447g0040	−36.50458069	5.11045E-07	0.001114988	Zinc finger, RING-type, Cellulose synthase, RING-type zinc finger, Cellulose synthase, Zinc finger, RING/FYVE/PHD-type
Nitab4.5_0000098g0090	−36.11111111	2.49283E-07	0.000630369	Double-stranded RNA-binding domain
Nitab4.5_0000165g0090	−35.71428571	6.57588E-06	0.007967881	Unknown
Nitab4.5_0003364g0080	−35.71428571	2.66101E-06	0.003997862	Tetratricopeptide-like helical, Tetratricopeptide repeat, Tetratricopeptide TPR1, Tetratricopeptide repeat-containing domain
Nitab4.5_0005249g0060	−35.69503273	1.12086E-06	0.002056563	Vacuolar import/degradation, Vid27-related, WD40-repeat-containing domain, WD40/YVTN repeat-like-containing domain
Nitab4.5_0002167g0040	−35.48387097	4.83572E-06	0.00627865	Cytochrome b-c1 complex subunit 8, plants, Cytochrome b-c1 complex subunit 8
Nitab4.5_0002354g0130	−35.48387097	1.10287E-06	0.002033845	Tetratricopeptide repeat-containing domain, Tetratricopeptide repeat, Tetratricopeptide-like helical
Nitab4.5_0004886g0010	−35.41666667	2.43087E-06	0.003738086	Zinc finger, CCCH-type, K Homology domain, type 1, K Homology domain
Nitab4.5_0006120g0070	−35.29411765	6.52467E-07	0.001346066	NB-ARC, P-loop containing nucleoside triphosphate hydrolase
Nitab4.5_0005249g0060	−35.18518519	1.9231E-08	7.84843E-05	Vacuolar import/degradation, Vid27-related, WD40-repeat-containing domain, WD40/YVTN repeat-like-containing domain
Nitab4.5_0001296g0080	−34.375	6.36176E-06	0.007766065	CID domain, RNA polymerase II-binding domain, ENTH/VHS
Nitab4.5_0011177g0030	−34.34130333	2.70088E-06	0.004037314	Unknown
Nitab4.5_0007057g0010	−34.1503268	8.45787E-06	0.009588762	HIP116, Rad5p N-terminal, VRR-NUC domain, Zinc finger, Rad18-type putative
Nitab4.5_0000688g0090	−34.04255319	8.54034E-08	0.000268394	GDP-fucose protein O-fucosyltransferase, O-fucosyltransferase, plant
Nitab4.5_0010819g0010	−34	1.8892E-06	0.003098468	Mediator complex, subunit Med17
Nitab4.5_0000573g0090	−33.81808567	6.29016E-07	0.001307213	Dcp1-like decapping, Pleckstrin homology-like domain
Nitab4.5_0002941g0070	−33.33333333	6.72281E-06	0.008092803	Pentatricopeptide repeat, Tetratricopeptide-like helical
Nitab4.5_0002983g0010	−33.04738562	2.52132E-07	0.000636318	Mannose-binding lectin, P-loop containing nucleoside triphosphate

				hydrolase, NB-ARC, Disease resistance protein
Nitab4.5_0011177g0030	−32.69230769	1.70963E-06	0.00285661	Unknown
Nitab4.5_0000106g0390	−32.60930103	3.99005E-10	3.20847E-06	P-loop containing nucleoside triphosphate hydrolase, ABC transporter-like, AAA+ ATPase domain, ABC-2 type transporter
Nitab4.5_0019126g0020	−32.36363636	4.31596E-06	0.005782403	WD40/YVTN repeat-like-containing domain
Nitab4.5_0003314g0020	−32.35294118	7.57859E-06	0.008878787	Armadillo-type fold, Armadillo-like helical
Nitab4.5_0005485g0010	−31.95386703	3.29223E-06	0.004709551	Mannose-binding lectin, NB-ARC, P-loop containing nucleoside triphosphate hydrolase, Disease resistance protein
Nitab4.5_0001875g0130	−31.91489362	3.54304E-06	0.004969384	Kinesin, motor domain, Cytochrome P450, Kinesin-like protein, Kinesin, motor region, conserved site, P-loop containing nucleoside triphosphate hydrolase
Nitab4.5_0001802g0080	−31.91489362	4.78219E-07	0.001060811	Sas10 C-terminal domain, Sas10/Utp3/C1D
Nitab4.5_0001296g0080	−31.81818182	5.51071E-06	0.006916479	CID domain, RNA polymerase II-binding domain, ENTH/VHS
Nitab4.5_0000464g0090	−31.74603175	1.04812E-07	0.000314491	Pentatricopeptide repeat, Tetratricopeptide-like helical
Nitab4.5_0029014g0010	−31.64639242	4.61721E-06	0.006076712	Protein-tyrosine phosphatase, receptor/non-receptor type, Protein-tyrosine/Dual specificity phosphatase, Protein-tyrosine phosphatase, catalytic
Nitab4.5_0000464g0110	−31.57894737	3.53502E-06	0.004959934	Mitotic checkpoint serine/threonine protein kinase Bub1/Mitotic spindle checkpoint component Mad3, Mad3/BUB1 homology region 1
Nitab4.5_0000051g0030	−31.19958366	4.77907E-07	0.001060811	Unknown
Nitab4.5_0006124g0020	−31.14795918	4.11093E-06	0.005553278	Disease resistance protein, P-loop containing nucleoside triphosphate hydrolase, Phosphotyrosyl phosphatase activator, PTPA, NB-ARC
Nitab4.5_0000391g0190	−30.90909091	2.84136E-07	0.000704653	Pentatricopeptide repeat, Tetratricopeptide-like helical
Nitab4.5_0029014g0010	−30.576956	1.42336E-07	0.000401542	Protein-tyrosine phosphatase, receptor/non-receptor type, Protein-tyrosine/Dual specificity phosphatase, Protein-tyrosine phosphatase, catalytic
Nitab4.5_0000106g0390	−30.56182189	4.96505E-09	2.5993E-05	P-loop containing nucleoside triphosphate hydrolase, ABC transporter-like, AAA+ ATPase domain, ABC-2 type transporter
Nitab4.5_0000159g0010	−30.3030303	3.38451E-06	0.004822066	Magnesium transporter NIPA
Nitab4.5_0000464g0090	−30.15873016	5.10446E-07	0.001114253	Pentatricopeptide repeat, Tetratricopeptide-like helical

Nitab4.5_0007929g0030	−30	5.54492E-06	0.006936591	Pentatricopeptide repeat, Tetratricopeptide-like helical
Nitab4.5_0003956g0020	−30	1.39997E-06	0.002444814	Bromo adjacent homology (BAH) domain
Nitab4.5_0001490g0030	−29.96254682	4.24271E-07	0.000969506	Unknown
Nitab4.5_0012388g0010	−29.83870968	2.59935E-07	0.000653413	Protein kinase-like domain, Protein kinase domain, Serine/threonine- / dual specificity protein kinase, catalytic domain, Phosphoribulokinase, Phosphoribulokinase/uridine kinase, Protein kinase, ATP binding site, Serine/threonine-protein kinase, active site
Nitab4.5_0004389g0030	−29.78723404	9.61421E-08	0.000294822	Helicase, superfamily 1/2, ATP-binding domain, Helicase, C-terminal, P-loop containing nucleoside triphosphate hydrolase, RNA helicase, DEAD-box type, Q motif, DNA/RNA helicase, DEAD/DEAH box type, N-terminal
Nitab4.5_0007467g0010	−29.66269841	1.01453E-06	0.00190258	AAA+ ATPase domain, Helicase, superfamily 1/2, ATP-binding domain, P-loop containing nucleoside triphosphate hydrolase, Helicase, C-terminal, Sec63 domain, DNA/RNA helicase, DEAD/DEAH box type, N-terminal
Nitab4.5_0000106g0390	−28.96981068	3.38112E-07	0.000813795	P-loop containing nucleoside triphosphate hydrolase, ABC transporter-like, AAA+ ATPase domain, ABC-2 type transporter
Nitab4.5_0000051g0030	−27.08333333	1.54994E-06	0.002641761	Unknown
Nitab4.5_0001088g0090	−26.67004049	1.37948E-08	5.98864E-05	HEAT, type 2, Armadillo-like helical, Armadillo-type fold
Nitab4.5_0012631g0020	−26.63690476	2.47968E-07	0.000628542	COBRA, plant
Nitab4.5_0006926g0010	−25.71428571	3.8395E-06	0.005275566	Exocyst complex component Sec10-like
Nitab4.5_0009433g0010	−25.42938931	7.67016E-06	0.008956405	NB-ARC, Mannose-binding lectin, Disease resistance protein, P-loop containing nucleoside triphosphate hydrolase
**CONTEXT CHG**				
**ID**	**meth.diff**	***p* value**	**q value**	**Note**
Nitab4.5_0000725g0100	−75	1.50E-09	0.000330562	UDP-glucuronosyl/UDP-glucosyltransferase
Nitab4.5_0003013g0030	−74.07407407	2.74E-09	0.000527999	WD40/YVTN repeat-like-containing domain, Quinonprotein alcohol dehydrogenase-like superfamily
Nitab4.5_0009641g0020	−72.15384615	1.34E-08	0.001741662	SANT/Myb domain, Linker histone H1/H5, domain H15, Homeodomain-like, Myb domain, Winged helix-turn-helix DNA-binding domain
Nitab4.5_0003943g002	−69.56521739	1.53E-08	0.001877978	Oxysterol-binding protein,

0				conserved site, Oxysterol-binding protein
Nitab4.5_0000083g0210	−65.68544996	1.55E-08	0.001882851	Ubiquitin carboxyl-terminal hydrolases family 2, Peptidase C19, ubiquitin carboxyl-terminal hydrolase 2, conserved site
Nitab4.5_0001319g0010	−65.625	1.84E-07	0.00942114	Pyridoxal phosphate-dependent transferase, major region, subdomain 2, EGF-like, alliinase, Pyridoxal phosphate-dependent transferase, major region, subdomain 1, Pyridoxal phosphate-dependent transferase, Allinase, C-terminal
Nitab4.5_0000555g0110	−65.24064171	3.41E-08	0.003228529	DNA-binding pseudobarrel domain, B3 DNA binding domain
Nitab4.5_0008661g0030	−63.33333333	2.96E-08	0.002933379	Hydroxymethylglutaryl-CoA reductase, class I/II, catalytic domain, Hydroxymethylglutaryl-CoA reductase, class I/II, Hydroxymethylglutaryl-CoA reductase, class I/II, substrate-binding, Hydroxymethylglutaryl-CoA reductase, class I/II, NAD/NADP-binding
Nitab4.5_0007680g0020	−62.96296296	4.04E-08	0.003647358	Unknown
Nitab4.5_0001296g0080	−58.64516129	1.13E-07	0.007149499	CID domain, RNA polymerase II-binding domain, ENTH/VHS
Nitab4.5_0003943g0020	−51.42857143	7.80E-08	0.005730805	Oxysterol-binding protein, conserved site, Oxysterol-binding protein
Nitab4.5_0003566g0010	−51.38498595	5.50E-08	0.004589442	Unknown
Nitab4.5_0003943g0020	−51.28205128	7.09E-08	0.005333796	Oxysterol-binding protein, conserved site, Oxysterol-binding protein
Nitab4.5_0006489g0010	−50	2.46E-09	0.000488237	Glycosyl transferase, family 2
Nitab4.5_0001296g0080	−45.48440066	1.68E-07	0.008984461	CID domain, RNA polymerase II-binding domain, ENTH/VHS
Nitab4.5_0029014g0010	−44.01440144	3.21E-09	0.000595568	Protein-tyrosine phosphatase, receptor/non-receptor type, Protein-tyrosine/Dual specificity phosphatase, Protein-tyrosine phosphatase, catalytic
Nitab4.5_0000688g0090	−40	2.08E-08	0.002386401	GDP-fucose protein O-fucosyltransferase, O-fucosyltransferase, plant
Nitab4.5_0000688g0090	−37.28813559	3.02E-09	0.000566624	GDP-fucose protein O-fucosyltransferase, O-fucosyltransferase, plant
Nitab4.5_0029014g0010	−32.15789474	6.74E-08	0.005180803	Protein-tyrosine phosphatase, receptor/non-receptor type,

				Protein-tyrosine/Dual specificity phosphatase, Protein-tyrosine phosphatase, catalytic
Nitab4.5_0029014g0010	−31.04399047	1.92E-07	0.009616571	Protein-tyrosine phosphatase, receptor/non-receptor type, Protein-tyrosine/Dual specificity phosphatase, Protein-tyrosine phosphatase, catalytic
Nitab4.5_0019152g0020	−28.81152461	9.84E-09	0.001362595	Unknown
Nitab4.5_0029014g0010	−28.12971342	1.71E-07	0.009074246	Protein-tyrosine phosphatase, receptor/non-receptor type, Protein-tyrosine/Dual specificity phosphatase, Protein-tyrosine phosphatase, catalytic
Nitab4.5_0000106g0390	−27.08494208	8.67E-09	0.001259864	P-loop containing nucleoside triphosphate hydrolase, ABC transporter-like, AAA+ ATPase domain, ABC-2 type transporter
